# AAV‐mediated delivery of an anti‐BACE1 VHH alleviates pathology in an Alzheimer's disease model

**DOI:** 10.15252/emmm.201809824

**Published:** 2022-03-30

**Authors:** Marika Marino, Lujia Zhou, Melvin Y Rincon, Zsuzsanna Callaerts‐Vegh, Jens Verhaert, Jérôme Wahis, Eline Creemers, Lidia Yshii, Keimpe Wierda, Takashi Saito, Catherine Marneffe, Iryna Voytyuk, Yessica Wouters, Maarten Dewilde, Sandra I Duqué, Cécile Vincke, Yona Levites, Todd E Golde, Takaomi C Saido, Serge Muyldermans, Adrian Liston, Bart De Strooper, Matthew G Holt

**Affiliations:** ^1^ VIB‐KU Leuven Center for Brain and Disease Research Leuven Belgium; ^2^ Department of Neurosciences KU Leuven Leuven Belgium; ^3^ Faculty of Psychology Laboratory of Biological Psychology KU Leuven Leuven Belgium; ^4^ Electrophysiology Expertise Unit VIB‐KU Leuven Center for Brain and Disease Research Leuven Belgium; ^5^ Department of Microbiology, Immunology and Transplantation KU Leuven Leuven Belgium; ^6^ Department of Neurocognitive Science Institute of Brain Science Nagoya City University Graduate School of Medical Sciences Nagoya Japan; ^7^ Laboratory of Cellular and Molecular Immunology Vrije Universiteit Brussel Brussels Belgium; ^8^ Department of Neuroscience Center for Translational Research in Neurodegenerative Disease McKnight Brain Institute College of Medicine University of Florida Gainesville FL USA; ^9^ Laboratory for Proteolytic Neuroscience RIKEN Brain Science Institute Wako‐shi Japan; ^10^ Immunology Programme The Babraham Institute Cambridge UK; ^11^ UK Dementia Research institute at UCL London UK; ^12^ Leuven Brain Institute Leuven Belgium; ^13^ Instituto de Investigação e Inovação em Saúde (i3S) University of Porto Porto Portugal

**Keywords:** AAV, Alzheimer’s disease, anti‐BACE1, VHH, Genetics, Gene Therapy & Genetic Disease, Neuroscience

## Abstract

Single domain antibodies (VHHs) are potentially disruptive therapeutics, with important biological value for treatment of several diseases, including neurological disorders. However, VHHs have not been widely used in the central nervous system (CNS), largely because of their restricted blood–brain barrier (BBB) penetration. Here, we propose a gene transfer strategy based on BBB‐crossing adeno‐associated virus (AAV)‐based vectors to deliver VHH directly into the CNS. As a proof‐of‐concept, we explored the potential of AAV‐delivered VHH to inhibit BACE1, a well‐characterized target in Alzheimer’s disease. First, we generated a panel of VHHs targeting BACE1, one of which, VHH‐B9, shows high selectivity for BACE1 and efficacy in lowering BACE1 activity *in vitro*. We further demonstrate that a single systemic dose of AAV‐VHH‐B9 produces positive long‐term (12 months plus) effects on amyloid load, neuroinflammation, synaptic function, and cognitive performance, in the *App^NL‐G‐F^* Alzheimer’s mouse model. These results constitute a novel therapeutic approach for neurodegenerative diseases, which is applicable to a range of CNS disease targets.

## Graphical abstract

VHH and blood‐brain‐barrier (BBB)‐crossing AAV‐based vectors are combined to achieve highly‐specific, long‐term BACE1 inhibition in a mouse model of Alzheimer's disease (AD).

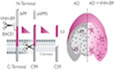

**The paper explained**

**Problem**

VHHs (also known as nanobodies) are antibody fragments, consisting of a single monomeric variable antibody domain, which retains the ability to bind selectively to a specific antigen. Their small size and unique mode of antigen binding allow them to access unique epitopes not available to conventional antibodies, such as enzyme active sites, which are often key drug targets, presenting unique therapeutic opportunities. Despite demonstrated efficacy in treating peripheral conditions, VHHs are still not widely used to treat central nervous system (CNS) disorders, as systemically injected VHH are prevented from entering the brain by the blood–brain barrier (BBB). One potential strategy to overcome this obstacle is to use AAV vectors with BBB crossing properties, such as AAV‐PHP.B, to transfer the VHH encoding gene into the CNS, allowing long‐term local production.

**Results**

As a proof‐of‐concept, we explored the potential of AAV‐delivered VHH to inhibit BACE1, a well‐characterized target in Alzheimer’s disease. One single systemic administration of AAV‐PHP.B allowed brain‐wide, long‐lasting production of an anti‐BACE1 VHH (B9) in a Alzheimer’s mouse model (*App^NL‐G‐F^*), which persisted for up to 12‐month post‐administration. Sustained VHH‐B9 expression produced positive long‐term (12 months plus) effects on amyloid load, neuroinflammation, synaptic function, and cognitive performance in treated animals.

**Impact**

AAV‐mediated delivery of VHH is a potentially disruptive technology to treat various CNS disorders—an area of large unmet clinical need. This utility largely derives from the unique properties of VHH, including their ease of manufacture and engineering, high target specificity and safety, which makes them an attractive alternative to conventional small molecule drugs. Ongoing improvements in AAV technology will likely produce serotypes with improved BBB penetration, which can be produced at scale and at low cost. This will facilitate clinical uptake, providing minimally invasive options for the sustained, cell‐specific expression of therapeutics within the CNS.

## Introduction

The high selectivity of monoclonal antibodies (mAbs) offers unique opportunities to target key proteins involved in the etiology of neurodegenerative conditions, such as Parkinson’s disease and Alzheimer’s disease (AD) (Zhou *et al*, 2011; Panza *et al*, 2014). However, their potential as central nervous system (CNS) therapeutics is largely limited by their inability to cross the blood–brain barrier (BBB) (Zafir‐Lavie *et al*, 2018), comparatively poor biodistribution through the parenchyma (Freskgård & Urich, 2017), and short half‐life (Wang *et al*, 2018). In addition, there is the potential for Fc receptor‐mediated immunogenicity, mediated by microglia, which can cause vasogenic edema and cerebral microhemorrhage (Panza *et al*, 2014).

Single variable domain antibodies (VHHs) are increasingly seen as an alternative to mAbs for therapeutic use (Steeland *et al*, 2016; Bannas *et al*, 2017; Gomes *et al*, 2018; Jovčevska & Muyldermans, 2020). In fact, the VHH‐based therapeutic Cablivi^®^ (caplacizumab‐yhdp) was recently approved for market by the US Food and Drug Administration (Scully *et al*, 2019) for peripheral treatment of adults with acquired Thrombotic Thrombocytopenic Purpura (aTTP). One key reason for their attractiveness over conventional mAbs is their small size and unique structure of their hypervariable complementarity determining regions (CDR), which allows them to access distinctive epitopes not available to conventional mAbs, such as enzyme active sites, which are often key drug targets (Hassanzadeh‐Ghassabeh *et al*, 2013). In addition, VHHs have a much lower immunogenic profile than traditional mAbs, largely due to the lack of an Fc‐region (Ackaert *et al*, 2021). Although there have been reports that VHH can pass through the BBB (Li *et al*, 2016), the degree of penetration is variable and linked to the intrinsic charge on the protein (Bélanger *et al*, 2019). Unfortunately, the amount reaching the CNS, following peripheral injection, is further compromised by their high rate of peripheral clearance, due to renal excretion (Gainkam *et al*, 2008; Bannas *et al*, 2015). Although VHHs which do actually reach the CNS (or are directly injected into the CNS) typically have a longer half‐life than in plasma, detectable levels are still reduced by up to 50% twenty‐four hours post‐injection (Dorresteijn *et al*, 2015). Genetic delivery of therapeutic VHHs to the CNS offers a potential solution to these issues, allowing long‐term local production (Zafir‐Lavie *et al*, 2018).

Adeno‐associated virus (AAV)‐based vectors are becoming the vehicle of choice for gene therapy applications, particularly for CNS applications, due to their high efficiency of gene transfer (including to post‐mitotic cells) and excellent safety profile (reviewed extensively in Hudry & Vandenberghe, 2019). Furthermore, the identification of BBB‐crossing AAV serotypes has opened up the opportunity of minimally invasive vector administration, with uniform gene delivery across the CNS (Deverman *et al*, 2018). AAV‐based vectors have one major drawback, however, in that their cargo capacity is very limited: transgenes larger than 5 kb in size are not efficiently packed into the vector (Trapani *et al*, 2014). Therefore, VHHs are ideal candidates for AAV‐mediated delivery, as they are comparatively small (typically 350 bp in size) and can be easily incorporated into AAV vectors (Verhelle *et al*, 2017) without extensive modifications that can adversely affect their binding properties (Pain *et al*, 2015). The latter is generally an issue with AAV‐mediated delivery of mAbs, or derivative formats such as single chain fragment variable (scFv) antibodies (Wu *et al*, 2010; Ahmad *et al*, 2012). In theory, their small size also allows for additional engineering, for example, directing the VHH into specific trafficking pathways to improve target engagement (Dmitriev *et al*, 2016), or incorporating specific proteolysis‐promoting sequences to stimulate intracellular degradation of toxic species (Baudisch *et al*, 2018).

In this work, we describe an efficient AAV‐based system for delivery of therapeutic VHH to the CNS. As a proof‐of‐concept strategy, we targeted the β‐site amyloid precursor protein‐cleaving enzyme 1 (BACE1), which is a key component in amyloid beta peptide (Aβ) production in AD (Vassar *et al*, 1999; Cai *et al*, 2001). BACE1 is an ideal target with which to explore the potential of AAV‐mediated VHH in the CNS. BACE1 is constitutively active (Colombo *et al*, 2013) and has been shown genetically to account for the majority of Aβ production *in vivo* (Atwal *et al*, 2011). Thus, we reasoned that measuring Aβ concentration in the CNS would provide a direct and robust readout of the efficacy of AAV‐mediated VHH delivery. Hence, we adopted a two‐stage approach in which we first generated a novel VHH, named B9, and tested this antibody for BACE1 binding and inhibition using a variety of *in vitro* methods (Zhou *et al*, 2011). In a second phase, we then used the recently developed AAV‐PHP.B to deliver B9 into the CNS of an AD mouse model, producing positive long‐term (12 months plus) effects on amyloid load, neuroinflammation, synaptic function, and cognitive performance, after a single systemic injection. Together, our results demonstrate that VHH against key CNS disease targets can be produced and delivered efficiently using systemic application of gene therapy vectors. This approach constitutes a unique therapeutic avenue, not only for AD but also for the broad spectrum of CNS diseases.

## Results

### Production and identification of BACE1 inhibiting VHH

**Figure 1:**
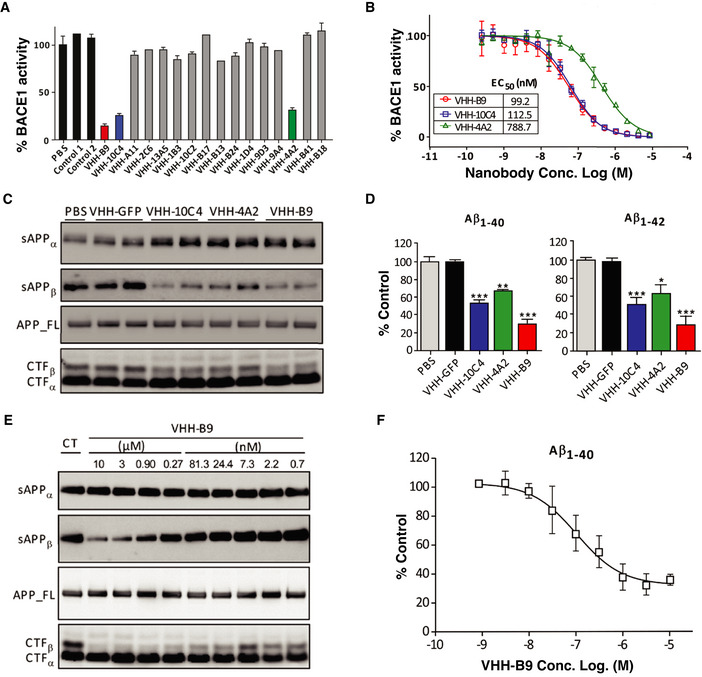
Characterization of anti‐BACE1 VHH AVHHs inhibiting BACE1 were identified using an *in vitro* APP cleavage assay. VHHs were recombinantly expressed in bacteria, purified and added to the assay at a final concentration of 5 μM. VHH‐B9, VHH‐10C4, and VHH‐4A2 consistently inhibited BACE1 activity, compared to PBS or control VHH (Aβ3 and BCIILP, raised against Aβ peptide and β‐lactamase BCII 659/H, respectively). Values are mean ± range, *n* = 2, technical replicates.BThe EC_50_ values (95% confidence interval) for VHH‐B9, VHH‐10C4, and VHH‐4A2 are 99.2 nM (83.8–117.5 nM), 112.5 nM (101.1–125.2 nM), and 788.7 nM (682.7–911.2 nM), respectively. Values are mean ± S.D., *n* = 3, technical replicates.C, DPrimary cultured neurons were transduced by Semliki Forest virus (SFV) expressing wild‐type human APP695 and treated with 3 µM of the indicated VHHs. PBS and anti‐GFP VHH were used as controls. (C) Western blot analysis of conditioned media for sAPP⍺ and sAPPβ, as well as cell extracts for full‐length APP, CTF⍺, and CTFβ. (D) ELISA measurements of Aβ_1‐40_ and Aβ_1‐42_ in conditioned media. Values are mean ± S.E.M., *n* = 3 cultures for each analysis. One‐way ANOVA, *P < 0.5, **P < 0.01, ***P < 0.0001.E, FDose‐dependent inhibition of BACE1 in primary cultured neurons by VHH‐B9. Cultured neurons were transduced by SFV expressing wild‐type human APP695 and treated with PBS (control: CT) or decreasing concentrations of VHH‐B9, ranging from 10 µM to 0.7 nM. (E) Western blot analysis of conditioned media for sAPP⍺ and sAPPβ, as well as cell extracts for full‐length APP, CTF⍺, and CTFβ. (F) Conditioned media was analyzed by ELISA to assess levels of Aβ_1‐40_. Values are mean ± S.D., *n* = 3 cultures for each analysis. The EC_50_ value (95% confidence interval) was estimated as 85.4 nM (52.5–125.6 nM).

**Figure EV1:**
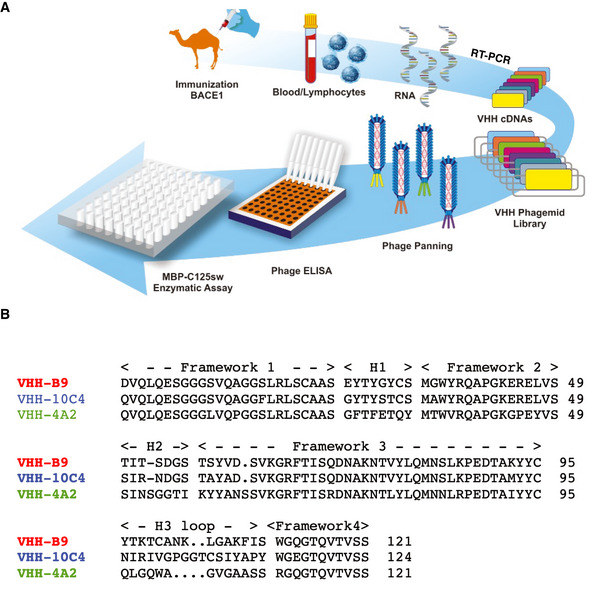
Anti‐BACE1 VHH production and selection Schematic summarizing the production procedure. A dromedary and a llama were immunized with recombinant human BACE1 ectodomain (amino acids 46–460). Blood lymphocytes from the immunized animals were collected for RNA extraction. cDNA was prepared and the variable fragments of heavy chain only IgGs were amplified by RT‐PCR and purified using agarose gel electrophoresis. cDNAs encoding VHH were cloned into the pHEN4 phagemid vector and phage libraries were prepared. Three rounds of consecutive phage panning were performed to enrich phage particles that bound recombinant BACE1 in an ELISA assay. Binding to BACE1 was confirmed in a phage ELISA. Finally, BACE1 binding VHHs were purified with initial testing of inhibitory activity using an *in vitro* APP cleavage assay (MBP‐C125sw enzymatic assay).Protein sequences for anti‐BACE1 VHH identified as having inhibitory activity in the *in vitro* APP cleavage assay. Sequences are aligned, with framework and CDR regions indicated.

To generate VHH, a dromedary and a llama were immunized with purified human BACE1 ectodomain (amino acids 46–460). In total, 16 different clones were identified that bind to BACE1. We screened for a possible effect of these VHH on BACE1 activity using an *in vitro* APP cleavage assay (Zhou *et al*, 2011). The 16 identified VHHs were recombinantly expressed in bacteria, purified and added to the assay at a final concentration of 5 μM. VHH inhibited BACE1 to varying degrees, with three VHH consistently giving the highest level of inhibition, with half‐maximal effective concentrations (EC_50_) (95% confidence interval) in the nanomolar range: 99.2 nM (83.8–117.5 nM) (VHH‐B9), 112.5 nM (101.1–125.2 nM) (VHH‐10C4), and 788.7 nM (682.7–911.2 nM) (VHH‐4A2) (Figs 1A and B, and EV1).

### Characterization of VHH‐BACE1 binding

**Figure EV2:**
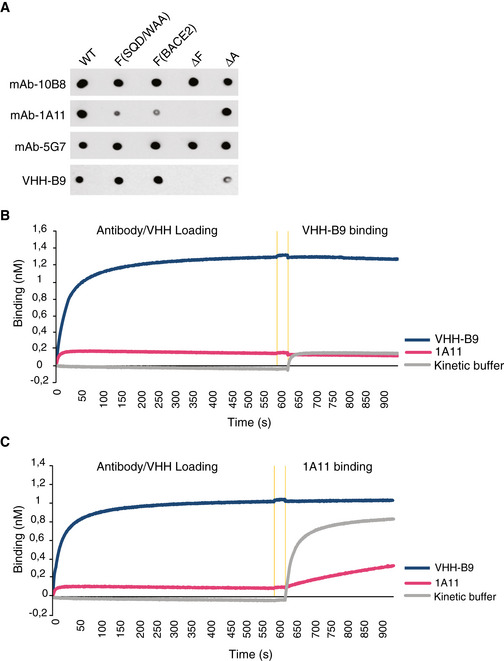
VHH‐B9 binding is specific to BACE1 with an epitope identical or overlapping the one targeted by the 1A11 anti‐BACE1 monoclonal antibody AVHH‐B9 binds to a unique exosite on BACE1. Wild‐type (WT) BACE1 ectodomain (1–460) or various mutants, including S376Q377D378/WAA (F(SQD/WAAA)), EDVATSQDD371‐379/MGAGLNYE (F(BACE2)), EVATSQD371‐378/EGS (ΔF), and GFPLNQSEVLASVG219‐232/GAG (ΔA), were purified from cultures of HEK293 cells. 200 ng purified protein was dotted onto a nitrocellulose membrane and probed with the indicated antibodies. Monoclonal antibodies 10B8, 5G7, and 1A11 were used as positive controls. 10B8 and 5G7 recognize all forms of BACE1 tested, indicating that the mutants were properly folded. 1A11 does not recognize BACE1 with mutations in loop F but recognizes Helix A mutants, as well as WT BACE1, as previously reported (Zhou *et al*, 2011). VHH‐B9 only weakly recognized ΔLoop F and ΔHelix A mutants, but recognized other Loop F mutants, S376Q377D378/WAA and EDVATSQDD371‐379/MGAGLNYE. Helix A is a structure adjacent to Loop F, flanking the active‐site cleft of BACE1. This suggests that VHH‐B9 binds via a conformational epitope engaging Helix A and Loop F, although direct binding to Loop F may not necessarily be needed. Binding to these structural elements, unique to BACE1, likely explains the specific inhibition seen with VHH‐B9.B, CVHH‐B9 and monoclonal antibody 1A11 compete for binding to BACE1. Epitope binning was done by biolayer interferometry (Octet RED96; Molecular Devices). Biotinylated BACE1 was bound to streptavidin sensor tips. Afterwards, BACE1‐loaded sensors were dipped in 1A11, VHH‐B9, or kinetic buffer for 600 s to allow binding. After a 30‐s equilibration in kinetic buffer (orange vertical lines), tips were dipped in VHH‐B9 or 1A11. (B) No additional binding of VHH‐B9 was observed. (C) A low level of 1A11 binding was observed. This residual binding is most probably due to the displacement of VHH‐B9 by 1A11, suggesting that these two antibodies bind to the same or adjacent epitopes.

More detailed examination of VHH‐BACE1 binding was obtained using the surface plasmon resonance (SPR) technique. The equilibrium dissociation constant (K_D_) was determined for VHH‐B9, VHH‐10C4, and VHH‐4A2 at both pH 7.0 (representing the pH of the extracellular environment) and pH 4.5 (pH of the endosomal compartment). In both cases, VHH‐B9 showed the highest affinity for BACE1. Interestingly, binding affinity seemed to be slightly stronger at pH 4.5, which is beneficial, as the majority of APP cleavage is reported to occur in the endosomal system (Sannerud *et al*, 2011; Ben Halima *et al*, 2016) (Table 1). Despite the high binding affinity of VHH‐B9 toward human and mouse BACE1, it did not show any binding to mouse BACE2 under the same measurement conditions, indicating a high degree of selectivity (Table 2), with epitope mapping suggesting VHH‐B9 binds to BACE1 at the structurally unique Helix A and Loop F (Zhou *et al*, 2011). These structural elements are close to the active site of the enzyme (Fig EV2), indicating that VHH‐B9 likely works by blocking effective substrate entry.

**Table 1:** Binding affinities of selected VHHs for BACE1. Measurements were made on a BIAcore instrument, using purified BACE1 ectodomain coupled to a CM5 chip. Binding constants were calculated at both pH 7.0 and pH 4.5 to confirm that the selected VHHs bind BACE1 under physiological conditions (extracellular space or trafficking endosome, respectively).

VHH	Binding affinities of selected VHH to human BACE1			
	pH	k_on_ (M^−1^ s^−1^)	k_off_ (s^−1^)	K_D_ (nM)
VHH‐B9	7.0	2.67 × 10^5^	9.80 × 10^‐4^	3.7
	4.5	6.62 × 10^5^	1.30 × 10^‐4^	1.9
VHH‐10C4	7.0	1.06 × 10^5^	7.92 × 10^‐3^	74.7
	4.5	4.51 × 10^5^	1.25 × 10^‐2^	27.7
VHH‐4A2	7.0	4.79 × 10^5^	2.31 × 10^‐2^	48.2
	4.5	3.97 × 10^5^	8.41 × 10^‐3^	21.2

**Table 2:** Binding affinities of VHH‐B9 for BACE1 and BACE2.

	Binding affinities of VHH‐B9 to BACE1 *vs*. BACE2			
	Conc. (nM)	k_on_ (M^−1^ s^−1^)	k_off_ (s^−1^)	K_D_ (nM)
mBACE1*	100	2.24 × 10^5^	7.20 × 10^‐4^	3.2
hBACE1*	100	1.53 × 10^5^	4.43 × 10^‐4^	2.9
mBACE2	100	NR	NR	NR*

h, human; m, mouse; NR, no response.

### VHH‐B9 effectively inhibits neuronal BACE1 in primary neuronal cultures

To test whether VHH‐B9 inhibits BACE1 in its native membrane environment, we turned to primary neuronal cultures. Cells were transduced by Semliki Forest Virus (SFV) expressing wild‐type human APP695 and then treated for 12 hours with recombinant VHH‐B9 at a final concentration of 3 μM. Treatment with VHH‐B9 decreased the levels of sAPPβ, CTFβ, Aβ_1–40_, and Aβ_1–42_ detected. Levels of full‐length APP, however, remained unchanged (Fig 1C and D) (Zhou *et al*, 2011; Tesseur *et al*, 2013). VHH‐B9 inhibits BACE1 in cultured neurons in a dose‐dependent manner (Fig 1E and F), with an EC_50_ (95% confidence interval) of 85.4 nM (52.5 nM to 125.6 nM). VHH‐10C4 and VHH‐4A2 were also tested in neuronal cultures. While both these VHHs inhibited BACE1 activity, VHH‐B9 was the most effective, as predicted from the *in vitro* assays. Together, these results indicate that VHH‐B9 efficiently inhibits BACE1 activity in its native neuronal environment.

### AAV serotype PHP.B allows safe, long‐term VHH‐B9 expression in the CNS following a single systemic dose

We next decided to determine whether AAV‐mediated B9 delivery (AAV‐VHH‐B9) could lower Aβ production and reduce Aβ‐related pathologies in a mouse model of Alzheimer’s disease. For these experiments, the cDNA for VHH‐B9 was modified to contain an N‐terminal BACE1 signal peptide to direct VHH to the BACE1 trafficking pathway to maximize the chance of productive interactions between the two proteins; a C‐terminal cMyc tag was also added to the VHH to aid detection. This cDNA sequence was then cloned into a standard AAV2‐based expression cassette, which also contained the ubiquitously active chimeric CAG promoter (cytomegalovirus early enhancer element/chicken β‐actin promoter/rabbit beta globin splice acceptor site), a woodchuck post‐transcriptional regulatory element (WPRE), and a bovine growth hormone poly(A) sequence (pA). This expression cassette was then packaged into an AAV capsid using standard protocols (Fripont *et al*, 2019). Given that Alzheimer’s disease shows brain‐wide pathology, we opted to use the BBB penetrant PHP.B serotype, which results in homogeneous CNS transduction, following a single, minimally invasive systemic injection (Rincon *et al*, 2018). In all AAV‐based experiments, a vector encoding GFP was used as a control.

**Figure 2:**
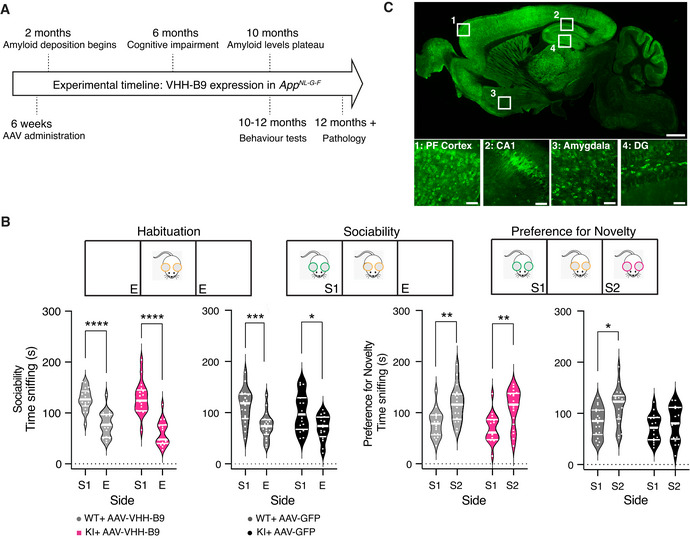
Long‐term BACE1 inhibition after AAV‐VHH‐B9 delivery reduces cognitive deficits in *App^NL‐G‐F^* mice Experimental timeline illustrating key points in the development of amyloidosis in the *App^NL‐G‐F^* mouse line along with key experimental manipulations. This longitudinal design allowed pathology to be performed on animals used in behavioral experiments (Figs 3, 4, 5).Sociability and social recognition memory in *App^NL‐G‐F^* (KI) mice and C57Bl/6J wild‐type (WT) controls were tested using a standard protocol (top). During sociability, control (grey plots) and *App^NL‐G‐F^* (colored plots) mice showed more interest in a strange mouse in an adjacent testing chamber [S1] than in an empty chamber [E]. This behavior was similar irrespective of VHH‐B9 or GFP treatment. Social recognition memory (preference for a novel mouse in S2 over S1) was observed in controls (gray plots) expressing VHH‐B9 or GFP. In *App^NL‐G‐F^* mice (magenta plots), VHH‐B9 prevented deficits in social recognition memory. Data are presented as violin plots. Median and quartiles (thick and thin white lines, respectively) indicate spread of data. Sociability *P = 0.01, ***P = 0.0005, ****P = 0.0001; Preference for social novelty *P = 0.04, **P = 0.006 (two‐way ANOVA with post‐hoc multiple comparisons using Šídák correction). Behavioral experiments with *App^NL‐G‐F^*: AAV‐VHH‐B9, *n* = 17; AAV‐GFP, *n* = 16. Experiments with C57Bl/6J mice: AAV‐VHH‐B9, *n* = 18; AAV‐GFP, *n* = 18, non‐injected, *n* = 18.Representative sagittal section from an *App^NL‐G‐F^* mouse injected at 6 weeks of age with AAV‐VHH‐B9. The brain was harvested 12‐month post‐injection and sagittal sections were immunostained for the cMyc tag fused to VHH‐B9. Note the widespread expression throughout the brain. Scale bar: 1 mm. Boxed sections are displayed at higher magnification in the images below. PF cortex, Prefrontal cortex: DG, Dentate gyrus. Scale bars: 50 µm.

Experiments were conducted in the *App^NL‐G‐F^* Alzheimer mouse model, in which the endogenous APP locus is modified to contain a humanized Aβ sequence containing three AD‐related mutations: Swedish, Iberian/Beyreuther, and Artic. The use of a knock‐in strategy to generate these mice ensures they accurately recapitulate endogenous APP expression, avoiding the overexpression artifacts typically observed in transgenic animals, making them a preferred platform for drug screening (Sasaguri *et al*, 2017). Due to the mutations in APP, these mice display increased total Aβ production, an increased Aβ_1–42/40_ ratio, and greater propensity to Aβ aggregation and plaque formation (Saito *et al*, 2014). The first detectable plaques occur between 2 and 4 months of age, with Aβ levels plateauing at approximately 9–10 months of age and plaques visible throughout the brain (Mehla *et al*, 2019), consistent with reported behavioral and cognitive deficits from 6 months of age in this model (Fig 2A) (Saito *et al*, 2014).

**Figure EV3:**
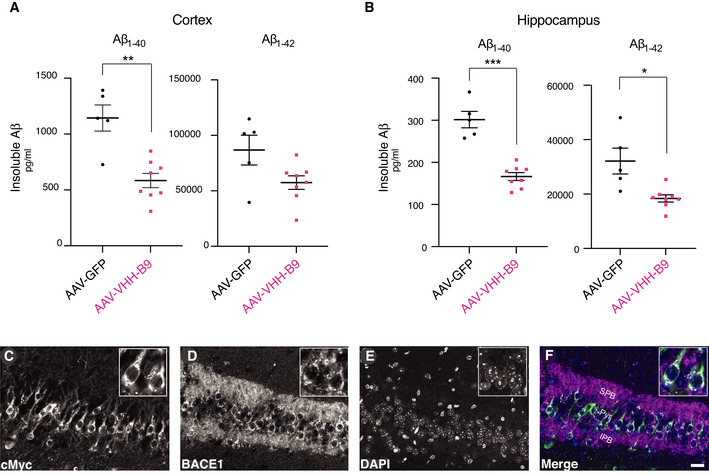
Proof‐of‐concept experiment for AAV‐mediated VHH‐B9 production and activity *in vivo* A, B
*App^NL‐G‐F^* mice were systemically injected with 1 × 10^12^ vg of AAV‐VHH‐B9 (*n* = 8) or the control vector AAV‐GFP (*n* = 5). Cortical samples (A) and hippocampi (B) were collected 12‐week post‐injection for analysis. Protein was extracted and used for quantification of insoluble Aβ_1−40_ and Aβ_1−42_ levels by ELISA. VHH‐B9 expression led to a significant decrease in both insoluble Aβ_1−40_ and Aβ_1−42_ levels. Each dot represents the mean between duplicate Aβ measurements in each individual mouse. Statistical analyses were performed with an unpaired *t*‐test with Welch’s correction. (A) **P = 0.004; (B) *P = 0.04, ***P = 0.0008.C–FVHH‐B9 co‐localized with BACE1 in hippocampal neurons following AAV‐mediated delivery. Representative images of coronal brain sections are shown for staining against BACE1 (magenta) and cMyc (green). Images show colocalization of VHH‐B9 with BACE1 in the pyramidal cell layer (Pyr), as well as in the suprapyramidal blade (SPB) and infrapyramidal blade (IPB) of the mossy fibers. The diffuse staining patterns suggest that VHH‐B9 is engaged with BACE1 in internal structures, such as early endosomes. Scale bar, 25 µm.

In a proof‐of‐concept study, designed to test VHH production and target engagement following systemic (tail vein) AAV injection, 6‐week‐old *App^NL‐G‐F^* mice were administered a dose of 1 × 10^12^ vector genomes (vg) per mouse of either AAV‐VHH‐B9 or AAV‐GFP (Appendix Table S1). Twelve‐week post‐injection, ELISA measurements showed that VHH‐B9 decreased Aβ_1–40_ and Aβ_1–42_ in the cortex and hippocampus of treated animals, in comparison with the control cohort (Fig EV3), consistent with colocalization of VHH‐B9 with BACE1 at the immunohistochemical level (Fig EV3). No apparent toxicity, derived from either the VHH or the vector, was observed in these small animal cohorts, based on overall survival rates. Hence, AAV‐mediated delivery is a feasible strategy for delivery of therapeutic nanobodies across the BBB into the CNS.

### Long‐term, systemic VHH‐B9 delivery reduces cognitive deficits in APP^NL‐G‐F^ mice

We decided to build on these proof‐of‐concept data by designing a longitudinal study to assess the possible beneficial effects of extended VHH expression/activity in an AD mouse model (Fig 2A). Experiments were designed based on large cohort sizes, allowing both reliable behavioral testing and detailed pathological examination post‐euthanasia. A description of all animal cohorts and the experiments performed is given in Appendix Table S1.

**Figure EV4:**
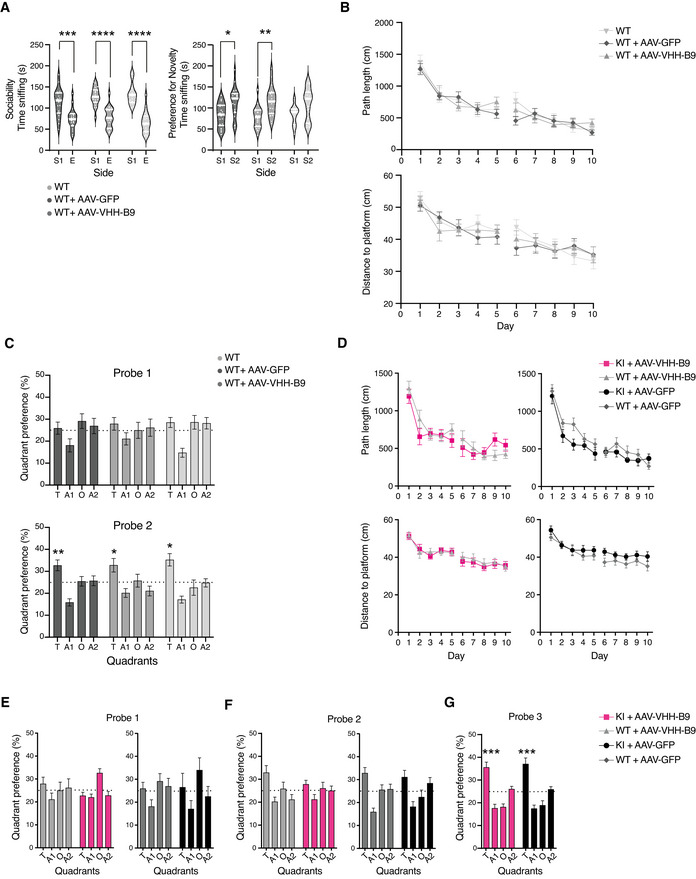
Long‐term BACE1 inhibition does not influence sociability and social recognition memory in C57Bl/6J cohorts and spatial learning in the Morris Water Maze in *App^NL‐G‐F^* mice ASociability and social recognition memory in C57Bl/6J cohorts. In a standard test for sociability (see also Fig 2), C57Bl/6J cohorts show normal sociability, preferring to interact with a novel mouse [S1] rather than an empty chamber [E]. Social recognition memory (preference for a novel mouse in S2 over S1) was observed in controls. Data are presented as violin plots. Median and quartiles (thick and thin white lines, respectively) indicate spread of data (*n* = 18 per group). Sociability ***P = 0.0005, ****P < 0.0001; Preference for social novelty *P = 0.004, **P = 0.006 (two‐way ANOVA with post‐hoc multiple comparison using Šídák correction).B, CSpatial learning in the Morris water maze in control groups. Mice were trained in a Morris Water Maze apparatus for 10 days (one trial per day) over two working weeks; the break in plots between days 5 and 6 indicates a pause in testing over a weekend. (B) C57BL/6J animals learned to rapidly locate a submerged platform in the pool over successive trials as indicated by a reduction in the total distance swum by the animal (path length) and directionality of the swim pattern (distance to platform from initial start point). No difference was observed between non‐injected (wild type: WT) animals and those receiving 1 × 10^12^ vg of either AAV‐VHH‐B9 or AAV‐GFP (C) Reference memory was assessed in ‘probe’ trials, by removing the submerged platform from the tank. No quadrant preference was observed after the first 5 days of training. However, all animals showed a robust preference for the quadrant previously containing the platform (T: target) during a second ‘probe’ trial, conducted after 10 days training. (A1/A2: adjacent quadrants; O: opposite quadrant). Data are presented as means ± SEM (WT+AAV‐B9 *n* = 18; WT+AAV‐GFP *n* = 17; WT *n* = 17). Statistical analyses were performed using a one sample *t*‐test. As all C57BL/6J cohorts learned equally well, only AAV‐injected wild types were used in comparisons with *App^NL‐G‐F^* (KI) mice. *P = 0.01–0.02; **P = 0.006 versus chance (One sample *T*‐Test).DSpatial learning in the Morris Water Maze for *App^NL‐G‐F^* (colored symbols) and C57BL/6J control mice (gray symbols). *App^NL‐G‐F^* performed similar to their respective C57Bl/6J controls. No difference was observed between genotypes after AAV‐VHH‐B9 (left) or AAV‐GFP (right) administration. Data are presented as means ± SEMs (KI+AAV‐VHH B9 *n* = 13; KI+AAV‐GFP *n* = 12; WT+AAV‐VHH‐B9 *n* = 16; WT+AAV‐GFP *n* = 14).E–GReference memory in *App^NL‐G‐F^* mice was assessed in interspersed probe trials. Time spent in the various pool quadrants was recorded as a measure for reference memory. (E) During the first probe trial (after 5 days trainings), both genotypes showed a similar quadrant preference, irrespective of AAV treatment paradigm. Preference for “T” was not above that dictated by chance (25%) (dotted line in the graph). (F) During the second probe trial (day 11), both C57Bl/6J cohorts (AAV‐VHH‐B9 or AAV‐GFP) showed a statistically significant preference for “T,” which was not detected in *App^NL‐G‐F^* mice. (G) *App^NL‐G‐F^* mice (injected with either AAV‐VHH‐B9 or AAV‐GFP) showed a clear preference for “T” following an additional week of swim training, indicating that long‐term expression of VHH‐B9 does not significantly impact spatial learning and reference memory. Data are presented as means ± SEMs (Probe 1 and 2: KI+AAV‐VHH‐B9 *n* = 17; KI+AAV‐GFP *n* = 16; WT+AAV‐VHH‐B9 *n* = 18; WT+AAV‐GFP *n* = 17. Probe 3: KI+AAV‐VHH‐B9 *n* = 16; KI+AAV‐GFP *n* = 12). ***P = 0.0004–0.0008 versus chance (One sample *T*‐Test)

*App^NL‐G‐F^* mice received a dose of 1 × 10^12^ vg/mouse of either AAV‐VHH‐B9 or AAV‐GFP via tail vein injection at 6 weeks of age. Three cohorts of age‐matched C57Bl/6J mice (AAV‐VHH‐B9 injected, AAV‐GFP injected, and wild type) were also included as controls for vector effect on normal learning and behavior. On reaching 10 months of age, we performed two behavioral tests associated with clinical signs of Alzheimer’s disease, including social preference and novelty memory, and spatial learning and reference memory (Latif‐Hernandez *et al*, 2019). During social preference and novelty testing, our *App^NL‐G‐F^* and C57Bl/6J cohorts showed normal social interactions, defined as a preference for interaction with a “novel” mouse (measured by the degree to which the mouse sniffed a compartment (S1) containing a novel mouse, in preference to an empty compartment (E) (Fig 2B, left). To test social memory, following this initial interaction, an additional “novel” mouse was placed into the opposing compartment (S2). As expected, preference for social novelty was observed in C57Bl/6J controls (Fig EV4A). *App^NL‐G‐F^* mice administered with AAV‐GFP, on the other hand, did not show any preference for interacting with a “new” mouse, indicating impaired social recognition memory (Fig 2B, right). In direct contrast, *App^NL‐G‐F^* mice injected with AAV‐VHH‐B9 did show significant interactions (Fig 2B, right). This result suggests that VHH‐B9 administration prevents, or at least slows, the decline in cognitive performance and impairment in social memory observed in *App^NL‐G‐F^* mice. AAV‐VHH‐B9 administration to *App^NL‐G‐F^* mice did not, however, improve performance in spatial learning and reference memory, tested using a standard Morris Water Maze (MWM) (Fig EV4B–G).

Following the conclusion of behavioral testing (roughly 12‐month post‐injection), animals were euthanized. Tissue sections were produced from a small number of mice and the persistence of VHH‐B9 expression and biodistribution checked using antibody staining for cMyc. Brain‐wide cell transduction and VHH‐B9 expression were observed, with particularly high levels observed in brain regions previously implicated in aspects of social behavior, specifically Prefrontal Cortex, Dentate gyrus, CA1, and Amygdala (Fig 2C) (Kogan *et al*, 2000; Broadbent *et al*, 2004; Huang *et al*, 2020; Botterill *et al*, 2021). To try and link improvements in behavior to pathology, we concentrated on these brain areas for our further analysis.

### Improved cognitive performance is related to decreased amyloid burden

**Figure 3:**
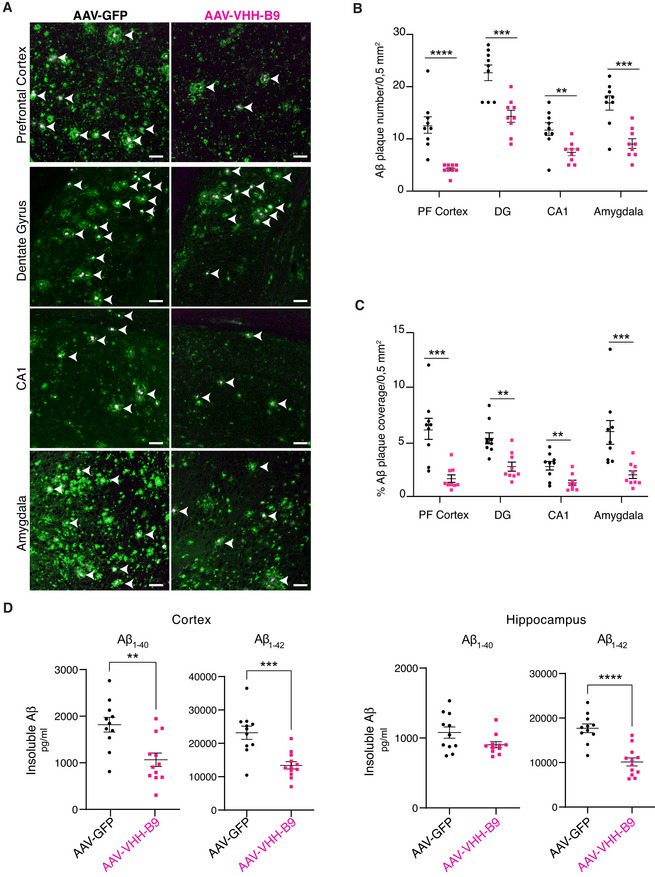
BACE1 inhibition in *App^NL‐G‐F^* mice by AAV‐VHH‐B9 delivery significantly reduces amyloid beta load AAV‐VHH‐B9 or AAV‐GFP (1 × 10^12^ vector genomes per mouse) were systemically injected into 6‐week‐old *App^NL‐G‐F^* mice. Brains were recovered approximately 12‐month post‐injection, following the completion of behavioral testing. ACo‐staining with anti‐N‐Terminal Aβ 82E1 (green) and Thioflavin‐S (magenta) reveals Aβ plaques (arrow heads) in regions of interest. Representative images are shown. Scale bar: 50 μm.B, CThe number of Aβ plaques in regions of interest was significantly reduced by treatment AAV‐VHH‐B9 (B), as was total plaque area (C).DQuantification of insoluble Aβ_1−40_ and Aβ_1−42_ in the cortex and hippocampus of *App^NL‐G‐F^* mice measured using ELISA. Each dot represents the mean between duplicate Aβ measurements in each individual mouse. Long‐term VHH‐B9 expression resulted in a significant reduction in the amyloid load. Data information: (B) and (C) show plaque load measured in three slices from each of three independent animals (selected based on their performance in the sociability and preference for social novelty test); each dot represents plaque load in an individual slice. Bars represent mean + SEM. Sample group size is shown in the graph. Statistical analyses for PFC in Fig 3B, and PFC and Amygdala in Fig 3C were performed with a Mann–Whitney test, due to a non‐normal data distribution. The remaining analyses were performed with an unpaired *t*‐test with Welch’s correction. (B) **P = 0.007, ***P = 0.0004–0.0006, ****P < 0.0001; (C) **P = 0.001–0.004, ***P = 0.0003–0.0005; (D) **P = 0.002, ***P = 0.0005, ****P < 0.0001.

**Figure EV5:**
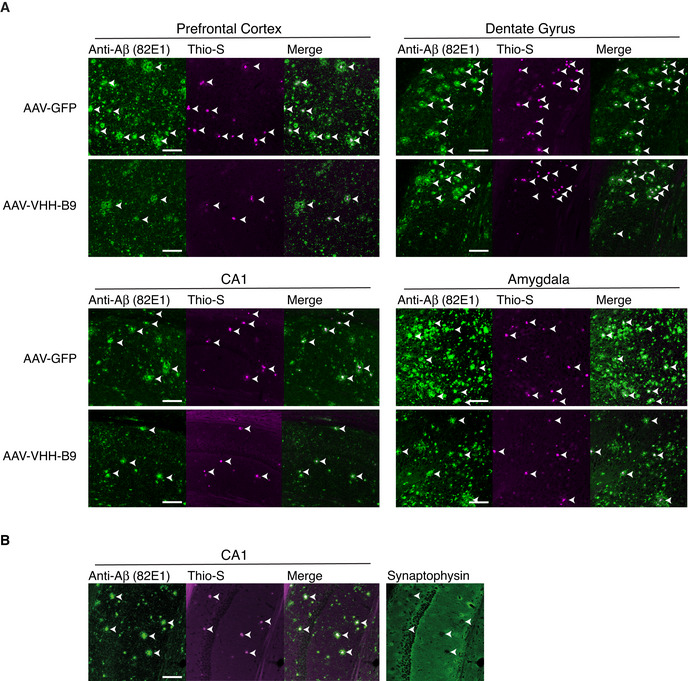
Amyloid plaque deposition in *App^NL‐G‐F^* mice correlates with synaptic loss and is reduced by AAV‐VHH‐B9 AAV‐VHH‐B9 or AAV‐GFP were systemically injected into *App^NL‐G‐F^* mice at a dose of 1 × 10^12^ vg, when mice were 6 weeks of age. Brains were recovered approximately 12‐month post‐injection, following behavioral testing. Immunostaining with anti‐N‐Terminal Aβ (82E1) (green) and co‐staining with Thioflavin‐S (magenta) reveal Aβ plaques (arrow heads) in the regions indicated. Individual channels were used to produce the merge images, which also appear in Fig 3A.Antibody staining against the synaptic vesicle marker synaptophysin (green) reveals loss of synaptic terminals in the immediate proximity of amyloid plaques (arrow heads, marked by 82E1 and Thioflavin S staining) in the hippocampal CA1 region. Data information: Scale bars, 100 μm.

A further set of tissue samples were used to determine Aβ content in AAV‐treated *App^NL‐G‐F^* mice. Wild‐type mice were excluded from this analysis as plaque deposition is not typically detected even at 24 months of age (Saito *et al*, 2014). Plaques were visualized using co‐staining with an anti‐Aβ antibody (green) and Thioflavin‐S (for beta sheet structure typical of amyloids) (magenta) (Figs 3A and EV5A). Aβ plaques were quantified and their surface areas were measured. We observed both a decrease in total plaque number and plaque size in all brain regions assessed (Fig 3B and C). Finally, ELISA experiments showed a significant decrease in the levels of both insoluble Aβ_1−40_ and insoluble Aβ_1−42_ in mice treated with AAV‐VHH‐B9 (Fig 3D).

### Reduced astrogliosis and microgliosis concomitant to reduced amyloid load

**Figure 4:**
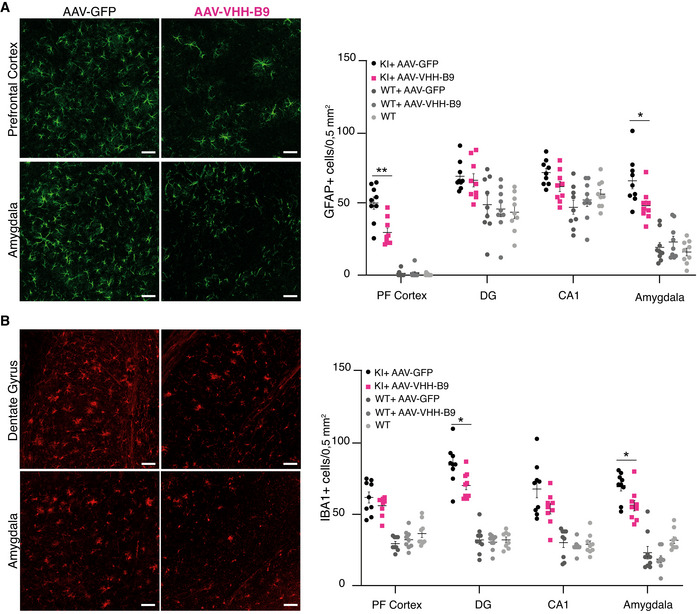
BACE1 inhibition in *App^NL‐G‐F^* mice by AAV‐VHH‐B9 delivery significantly reduces neuroinflammation AAV‐VHH‐B9 or AVV‐GFP were systemically injected into *App^NL‐G‐F^* mice at a dose of 1 × 10^12^ vg when mice were 6 weeks of age. Brains were recovered approximately 12‐month post‐injection following behavioral testing. (left) Representative images of anti‐GFAP immunostaining in Prefrontal Cortex and Amygdala from *App^NL‐G‐F^* mice receiving either AAV‐VHH‐B9 or AAV‐GFP; (right) quantification of the GFAP^+^ cell number in the indicated brain regions across experimental cohorts.(left) Representative images of anti‐IBA1 immunostaining in dentate gyrus and amygdala from *App^NL‐G‐F^* mice receiving either AAV‐VHH‐B9 or AAV‐GFP; (right) quantification of the IBA1^+^ cell number in the indicated brain regions across experimental cohorts. Data information: For both (A) and (B), cell numbers were measured in three slices from each of three independent animals (selected based on their performance in the sociability and preference for social novelty test); each dot represents cells counted in an individual slice. Bars represent mean + SEM. Sample group size is shown in the graph. Statistical analyses were performed with an Unpaired *t*‐test with Welch’s correction. (A) *P = 0.02, **P = 0.001; (B) *P = 0.01. Scale bars, 50 µm.

We next assessed whether AAV‐VHH‐B9 could reduce the level of neuroinflammation associated with amyloid deposition in *App^NL‐G‐F^* mice. Neuroinflammation is often associated with an increase in the number of both GFAP+ astrocytes and IBA1+ microglia surrounding plaques. Unfortunately, both Dentate Gyrus and CA1 contain GFAP+ astrocytes in wild‐type animals. However, AAV‐VHH‐B9 administration resulted in a significant difference in the number of reactive astrocytes in both Prefrontal Cortex and Amygdala (Fig 4A) in *App^NL‐G‐F^* mice. Likewise, microgliosis was also significantly reduced in both Dentate Gyrus and Amygdala (Fig 4B), with a trend toward reduced microgliosis in Prefrontal Cortex and CA1. Crucially, vector administration (VHH‐B9 or GFP) did not induce gliosis in wild‐type animals, indicating that the vector itself does not induce an inflammatory response, consistent with the overall lack of toxicity observed. Likewise, ELISAs for anti‐VHH indicated lack of a significant immune response against B9 (Appendix Fig S2).

### AAV‐VHH‐B9 treatment prevents loss of LTP in App^NL‐G‐F^ mice

**Figure 5:**
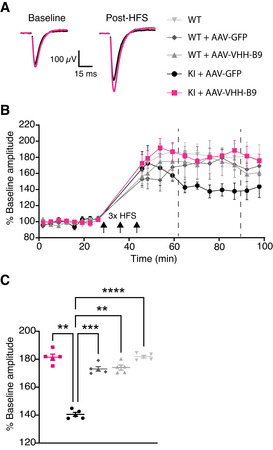
AAV‐VHH‐B9 prevents synaptic impairment in *App^NL‐G‐F^* mice Examples of the fEPSP traces recorded under baseline conditions (left) or post‐high‐frequency simulation (HFS) (right) in acute hippocampal slices obtained from *App^NL‐G‐F^* mice injected with either AAV‐VHH‐B9 (magenta) or AAV‐GFP (black). The break in the traces is due to removal of a stimulus artifact.Time course of averaged fEPSP amplitudes relative to baseline average for each experimental group. Three periods of HFS were applied at time points indicated with black arrows. Vertical dashed lines define the boundaries of the temporal window used to produce the plots in (C). Results are the average fEPSP amplitude ± SEM determined across all recorded slices (2 biological replicates per mouse, 5 independent mice per experimental group) for each time point measured.Average fEPSP amplitude values at individual acquisition points between min 75 and 90 of the recording (vertical dashed lines in B) across experimental groups. Individual symbols represent the averaged fEPSP amplitude measured in one animal (2 slices), with bars representing average ± SEM across all slices. Data information: Statistical analyses were performed with a Repeated Measures One‐way ANOVA followed by post‐hoc Tukey’s tests. **P = 0.001; ***P = 0.0006; ****P < 0.0001.

*App^NL‐G‐F^* mice typically show loss of hippocampal long‐term potentiation (LTP) from 6 months of age onwards (Latif‐Hernandez *et al*, 2020), which is thought to underlie, at least in part, the cognitive impairments that develop in this mouse strain (Saito *et al*, 2014; Mehla *et al*, 2019). To test whether we could correlate the observed improvement in cognitive performance to improved circuit function, we decided to test LTP induction in treated and control mice. We used acute hippocampal slices on a multi‐electrode array (MEA), allowing us to electrically stimulate the Schaffer collaterals while recording the evoked field excitatory post‐synaptic potentials (fEPSPs) in CA1. As expected, in wild‐type mice (including those treated with vector), application of a high‐frequency stimulation (HFS) protocol induced a potentiation in the recorded fEPSP amplitude (Fig 5A–C). In contrast, in *App^NL‐G‐F^* mice treated with AAV‐GFP, LTP was reduced (Fig 5A–C), consistent with previously published data showing synaptic impairment and reduced long‐term plasticity in this mouse model (Latif‐Hernandez *et al*, 2019). These defects in synaptic function are consistent with the reduction in (functional) synaptic connections in the immediate vicinity of Aβ plaques (Fig EV5B). Treatment with AAV‐VHH‐B9 prevented typical decline in LTP reported in *App^NL‐G‐F^* mice (Fig 5A–C).

Taken together, our data unequivocally demonstrate that a single systemic dose of AAV‐PHP.B allows long‐term, non‐toxic expression of VHH‐B9 in the CNS, producing significant effects on amyloid load, neuroinflammation, synaptic function, and cognitive performance in *App^NL‐G‐F^* mice.

## Discussion

To date, CNS diseases have been largely untreatable. A large class of CNS diseases are comprised of the so‐called “proteinopathies,” which are thought to be caused by mutant proteins with toxic effects. Therapies based on RNA interference (RNAi) and anti‐sense oligonucleotides (ASOs) are currently being trialed, but both approaches suffer from the fact that they typically target the whole protein, without any ability to distinguish between mutant and wild‐type protein conformations (Cao & Heng, 2005). CRISPR/Cas9 gene editing/correction remains an interesting possibility, but concerns remain about off‐target effects and long‐term toxicity (Zhang *et al*, 2015; Morgens *et al*, 2017).

In contrast, monoclonal antibodies have been widely adopted for clinical use because of their high specificity (including the ability to target independent functional domains and/or toxic epitopes/conformations) and good safety profile (Berger, 2002; Lu *et al*, 2020). Single variable domain antibodies (VHHs) are increasingly seen as an alternative to conventional monoclonal antibodies for therapeutic purposes (Steeland *et al*, 2016; Bannas *et al*, 2017; Gomes *et al*, 2018; Jovčevska & Muyldermans, 2020), as their small size and the unique structure of their antigen binding domain allows them to access unique epitopes not available to conventional antibodies. As such, VHHs have great potential to stabilize desired protein conformations (Steyaert & Kobilka, 2011), target toxic protein epitopes/conformations (Schut *et al*, 2015; Gettemans & De Dobbelaer, 2021), and/or block protein function (Lauwereys *et al*, 1998).

Despite the obvious attractiveness of antibody‐based therapies for CNS disease, there are three major issues which complicate their use. The first is the chronic, multi‐system nature of these diseases, the second is the shielding effect of the BBB which limits antibody access to the CNS from the systemic circulation, and the third is the restricted diffusion of antibodies through the parenchyma and uptake into cells (Levites *et al*, 2006; Wolak *et al*, 2015; Pardridge & Chou, 2021). Combined these three issues mean that constant dosing with high levels of antibody will be necessary to achieve any meaningful disease‐modifying effect (Ovacik & Lin, 2018), even those modified to allow transcytosis across the BBB (Pardridge & Chou, 2021). One possible strategy to generate long‐term antibody production and disease protection is antibody gene transfer with viral vectors, particularly AAV‐based vectors (Brady *et al*, 2017; Robert *et al*, 2017; Martinez‐Navio *et al*, 2020). Historically, however, this approach has suffered from a number of drawbacks. The most notable is that the vector requires direct injection of the AAV into the brain parenchyma which, despite the risks associated with such a surgical procedure (infection, hemorrhage, edema, etc.), only leads to transduction of the tissue immediately surrounding the injection site. This issue has been largely offset by the recent identification of BBB‐crossing AAVs, which combine minimally invasive vector administration with uniform gene delivery across the CNS (Deverman *et al*, 2018). The other drawback is that the limited transgene capacity of AAV means that extensive antibody reformatting is often necessary in order not to exceed the packaging limit. The format most commonly used is the so‐called single chain variable fragment (scFv), which is a fusion of the variable regions of the antibody heavy and light chains, separated by a short linker. Unfortunately, extensive optimization is generally needed to optimize both the length and hydrophilicity of the linker sequence, to ensure correct protein folding allowing efficient antigen binding (Ewert *et al*, 2004; Ahmad *et al*, 2012). This is further compounded by the fact that the highly reducing environment of the cell typically prevents the formation of disulfide bridges, which may be needed for assembly and stability (Soetens *et al*, 2020). In contrast, the smaller size, single binding domain, and stability of VHH make them ideal candidates for incorporation into AAVs. The excellent safety profile of these vectors, coupled with the long‐term transgene expression possible (up to 15 years in non‐human primates (Sehara *et al*, 2017) and 6 years in humans (Marks *et al*, 2016)), has made them the vector of choice for long‐term treatment of CNS conditions, with the new opportunities for homogeneous, brain‐wide gene transfer afforded by BBB crossing AAVs opening up the possibility of efficient treatment of multi‐system disorders (Deverman *et al*, 2018). In this respect, the recent approval of Zolgensma has revolutionized the treatment of pediatric spinal muscular atrophy (SMA) (Mendell *et al*, 2017), with significant increases in survival and motor skills reported in patients, following a single systemic injection of vector.

In this work, we set out to establish proof‐of‐principle for safe and efficient delivery of therapeutic VHH into the CNS using next‐generation BBB‐crossing AAVs, by targeting BACE1 an important CNS target central to AD pathology (Vassar *et al*, 1999; Barão *et al*, 2016; Voytyuk *et al*, 2018).

Through our VHH discovery platform, we identified several BACE1 inhibitors, of which VHH‐B9 emerged as the strongest candidate, based on specificity and degree of inhibition. Exploiting the intrinsic stability of VHHs and ease of engineering (Vincke *et al*, 2009), we further refined the system by incorporating a signal peptide into the VHH sequence, allowing intracellular expression and directed trafficking to the endosome, where the majority of BACE1‐mediated APP cleavage is thought to occur (Sannerud *et al*, 2011; Ben Halima *et al*, 2016). This was done with the aim of promoting VHH‐BACE1 interactions early in the secretory pathway, which may be beneficial as some mutations in APP, such as the *Swedish* mutation (*Swe*), are known to promote processing of APP in the secretory pathway itself (Sasaguri *et al*, 2017).

Exploiting the suitability of the VHH platform for use with AAV‐delivery, we then incorporated the cDNA for VHH‐B9 into the BBB crossing AAV serotype PHP.B, and performed a longitudinal study in the *App^NL‐G‐F^* mouse line. One single systemic administration of AAV‐PHP.B at 6 weeks of age allowed CNS‐wide transduction and sustained production of VHH‐B9 for in excess of 12‐month post‐vector administration, with no overt signs of toxicity. Consistent with a central role of BACE1 in Aβ production, long‐term VHH expression resulted in a significant reduction in insoluble Aβ accumulation and the number and size of Aβ plaques, significantly reduced the levels of astro‐ and microgliosis, and protected against loss of synaptic function. Although reports of cognitive deficits in the *App^NL‐G‐F^* mouse line have varied between laboratories (Saito *et al*, 2014; Whyte *et al*, 2018; Latif‐Hernandez *et al*, 2019), we could detect impairments in sociability and preference for social novelty, as well as spatial learning and reference memory, in untreated mice at 12 months of age. Crucially, treatment with VHH‐B9 appears to prevent, or at least slow, impairment in social memory in this mouse line, consistent with improved pathology in key brain areas associated with this behavior (Bicks *et al*, 2015; Okuyama *et al*, 2016; Ko, 2017; Hainmueller & Bartos, 2020; Huang *et al*, 2020). We did not, however, see any effect of VHH‐B9 on spatial learning and reference memory in the Morris Water Maze (MWM). Given the overall positive effects of VHH‐B9 expression, the most parsimonious explanation at this moment is that spatial learning and reference memory are more prone to disruption by Aβ accumulation and/or associated neuroinflammation, meaning a greater reduction in Aβ would be required to see any effect (see below).

In direct contrast, a traditional passive immunization, albeit with a different anti‐BACE1 VHH (VHH‐B3a; 75 µg), showed no significant decrease in the level of soluble Aβ_1–40_ in the APP/PS1 mouse line 24‐hour post‐injection (Dorresteijn *et al*, 2015). This presumably reflects the relative high dissociation constant of this VHH for BACE1 (0.3 µM), a gradual decrease in available VHH (possibly as a result of clearance), or a combination of both factors. A dose‐dependent inhibition of BACE1 activity was seen following direct parenchymal injections of conventional mAbs, or systemic administration of mAbs engineered to cross the BBB by receptor‐mediated transcytosis (Zhou *et al*, 2011; Cheng *et al*, 2013; Ye *et al*, 2017). In these examples, however, effective inhibition was typically limited to a period of up to 48‐hour post‐injection. Taken together, we believe these results largely validate our decision to use AAV‐mediated delivery of VHH.

The data we present here support the hypothesis that BACE1 inhibition will likely be beneficial for AD patients. Although there have been a number of clinical trials using BACE1 inhibitors that were terminated prematurely due to adverse side effects, there is reason to believe that these failures result from poor drug specificity, an incomplete understanding of BACE1 biology and incorrect trial design, leading to off‐target toxicity and/or issues of dosing (McDade *et al*, 2021). However, there are reasons to believe that a sustained steady state reduction, similar to that which we report, may be worthwhile pursuing. First, a reduction of 50% in the levels of BACE1 activity is sufficient to significantly reduce Aβ plaques, neuritic burden, and synaptic deficits in mouse models of AD (McConlogue *et al*, 2007), similar to the effects seen with a reduction of 30% in the levels of gamma secretase activity (Li *et al*, 2007). Second, a mutation in human APP, which reduces cleavage by BACE1 (Jonsson *et al*, 2012), leads to a reduction in cerebral Aβ of approximately 20%, with carriers showing lifelong protection against AD and cognitive decline (although issues with the mutation affecting aggregation of Aβ peptides cannot be fully excluded) (Benilova *et al*, 2014; Maloney *et al*, 2014). These observations provide strong support for continued efforts to target BACE1 therapeutically in AD (Hampel *et al*, 2021; McDade *et al*, 2021), although dosing and timing of treatment relative to disease progression will be critical, with reports suggesting that treatment will be more efficient early in disease before heavy plaque deposition (McDade *et al*, 2021). As such, effective treatment will also depend on improvements in related disciplines, including genetic testing, biomarker identification, and cognitive tests, to identify patients who can benefit from treatment.

Critically, partial long‐term inhibition of BACE1 may not only result in a positive suppression of plaque load, but may also reduce (or completely avoid) the known toxicity resulting from complete loss of BACE1 function, including issues of hypomyelination (Willem *et al*, 2006), aberrant synaptic homeostasis and plasticity (Filser *et al*, 2015), axon guidance abnormalities (Rajapaksha *et al*, 2011; Cao *et al*, 2012; Ou‐Yang *et al*, 2018), impairments in spatial and working memory (Egan *et al*, 2019; Henley *et al*, 2019; Knopman, 2019), and retinal pathology (Cai *et al*, 2012). In this respect, our observation that AAV‐mediated delivery of VHH‐B9 did not substantially affect cleavage of the alternative BACE1 substrate seizure protein 6 (SEZ6), which is important for maintenance of dendritic spines and synaptic plasticity (Gunnersen *et al*, 2007; Pigoni *et al*, 2016; Zhu *et al*, 2018), is interesting, as it implies that VHH‐B9 may be an APP‐selective BACE1 inhibitor, a finding consistent with the results we obtained on synaptic function and LTP induction. Such a tool would be enormously valuable for avoiding substrate‐specific side effects, and this will be followed up in future studies. The specificity of VHH‐B9 for BACE1 also avoids issues of impaired glucose homeostasis, hypopigmentation, seizures, and blindness (among others), which are associated with cross‐reactivity with BACE2, Cathepsin D, and Cathepsin E (Voytyuk *et al*, 2018).

Moving forward the system needs to be optimized to maximize reductions in Aβ with the minimal possible vector dose, to ensure safety. In this respect, adjustments can be made in both the vector system and VHH cargo.

As a proof‐of‐concept study, we were concerned with maximizing transduction of the CNS, following systemic delivery. As such, we used the recently developed AAV9 variant, PHP.B, which shows enhanced blood–brain barrier crossing and CNS transduction following intravenous dosing (Deverman *et al*, 2016; Rincon *et al*, 2018). However, the BBB crossing appears dependent on the presence of LY6A protein on vascular endothelial cells (Huang *et al*, 2019), the absence of which is thought to account for the lack of efficient CNS transduction in some primate studies (Hordeaux *et al*, 2018). Development of new capsids is a major ongoing research area (Li & Samulski, 2020). Identification of new AAVs, which combine enhanced BBB crossing and CNS transduction with lower off‐target transduction of peripheral tissues following systemic delivery, would allow lower vector doses to be used (Deverman *et al*, 2016). This would not only be beneficial from a safety perspective, reducing the possibility of potential immune responses to the vector and/or transgene (Verdera *et al*, 2020), but it would reduce manufacturing costs, which remain high and are currently a major hurdle to widespread adoption of vector‐based therapeutics (Deverman *et al*, 2018). In addition, engineering of novel capsids would likely circumvent issues with pre‐existing antibodies against naturally occurring AAV serotypes, which negatively impact vector efficiency. As these neutralizing antibodies are prevalent in human populations, they effectively limit the potential cohort of patients suitable for treatment with a given vector type (Louis Jeune *et al*, 2013).

Expression levels of a given VHH from a vector‐based system could be boosted, if necessary, by use of a self‐complementary genome configuration (Hudry & Vandenberghe, 2019). The use of specific promoter and or miRNA‐based systems, in combination with cell type specific transcriptional enhancers and inducible elements (Hudry & Vandenberghe, 2019), will also allow temporally controllable and graded levels of VHH expression in cell types of interest. An “on/off” switch would not only contribute greatly to the safety of a vector‐based system, but may also still produce sufficient therapeutic effect, as previous studies on BACE1 suggest that transient inhibition of the enzyme is sufficient to produce sustained long‐term reductions in Aβ accumulation (Karlnoski *et al*, 2009; Das *et al*, 2012). VHH themselves are also easily engineered and improvements in binding affinity (down to the low picomolar range) can be achieved using standard affinity maturation techniques (Mahajan *et al*, 2018), or alternatively via production of bivalent VHH constructs (Beirnaert *et al*, 2017) that bind distinct epitopes on a given target. Finally, as we demonstrate, VHH can be modified to add unique functions, such as signal peptide sequences (Vincke *et al*, 2009). This can be further exploited to include, for example, sequences targeting the VHH–antigen complex for degradation (Caussinus *et al*, 2011), while secretion signals could be added to direct VHH to the extracellular space (Goodwin *et al*, 2021). In this respect, the small size of VHH presents intriguing possibilities, whereby multiple independent VHH could be expressed against different targets; for example, intracellular (endosomal) targeting of BACE1 activity to lower Aβ production and extracellular targeting of Aβ to reduce existing plaques and prevent seeding and spreading of pathology (Gomez‐Gutierrez & Morales, 2020). Alternatively, VHH expression could be combined with delivery of a trophic factor, such as GDNF, to support cell survival (Gash *et al*, 2020). In reality, it is likely to be a combination of technological developments in the aforementioned areas that finally makes AAV‐mediated delivery of VHH a clinically relevant option for CNS disorders.

In summary, our data demonstrate for the first time the therapeutic (disease‐modifying) potential of combining VHH and next‐generation AAV‐based vectors. Moving forward, we propose that vector‐mediated VHH delivery can be successfully exploited for the treatment of a variety of CNS conditions with defined targets, such as Parkinson’s disease and amyotrophic lateral sclerosis, which are at present untreatable. As such, we think additional studies to optimize both VHH for these targets and the design of delivery vectors are warranted.

## Materials and Methods

### Animals

The *App^NL‐G‐F^* line (official strain name *App^tm3.1Tcs^/App^tm3.1Tcs^*) is a knock‐in model of Alzheimer’s type amyloidosis, in which the murine *App* gene has been replaced with a humanized version, containing mutations linked to familial forms of AD. The Swedish (NL), Iberian (F), and Arctic (G) mutations increase total Aβ production, Aβ_1–42_/Aβ_1–40_ ratio, and facilitate Aβ aggregation, respectively. The use of gene knock‐in ensures that issues associated with APP overproduction and mis‐localized expression are avoided. This mouse line recapitulates several symptoms associated with Alzheimer’s disease, the most prominent being synapse loss, microgliosis, astrogliosis, and age‐dependent cognitive decline.

All animal procedures were performed in accordance with the regulations of the Institutional Animal Care and Use Committee of KU Leuven (ethical permission document P209/2015). Animals were initially bred and housed in a specific pathogen free (SPF) facility. Following AAV injection, animals were moved to a conventional facility with controlled humidity, temperature, and light conditions. All the animals were housed in groups (max 5 animals/cage) within individually ventilated (IVC) cages. When necessary, animals were transferred to the behavioral testing facility, where they were housed in standard filter top cages (max 4 animals/cage). All cages contained wood chip bedding and were enriched with materials allowing nest building and concealment. Food and water were provided *ad libitum*.

### Immunization and VHH library construction

Two VHH libraries were generated using a protocol described previously, with minor modifications (Vincke *et al,* 2012). The first library was produced by immunization of a dromedary with recombinant non‐glycosylated human BACE1 ectodomain (amino acids 46–460), purified from insect cell cultures (Bruinzeel *et al,* 2002). The second library was produced by immunization of a llama with human BACE1 ectodomain purified from HEK293 cell cultures (Zhou *et al,* 2011). Briefly, the dromedary or llama received six subcutaneous immunizations at weekly intervals with 150 μg BACE1. Immune responses were evaluated using ELISAs, after purification of conventional IgG1 molecules, or the heavy‐chain‐only subclasses IgG2 and IgG3, from serum. In all, three different subclasses of IgGs immunoreactive against BACE1 were detected. The blood of the immunized animal was collected for lymphocyte preparation, followed by RNA isolation and RT‐PCR amplification. The cDNAs of the variable fragments of heavy‐chain‐only IgGs were cloned into the pHEN4 phagemid and transformed into electrocompetent TG1 *E. coli* cells to generate a library of 6 × 10^7^ transformants with 90% correct inserts from the dromedary, and another library of 5 × 10^7^ transformants with more than 78% correct inserts from the llama.

### Panning of VHH libraries

Panning of VHH libraries was performed according to a previously described protocol (Vincke *et al,* 2012), with two different strategies used to enrich BACE1‐binding VHHs. The first strategy used an ELISA‐based technique combined with high pH elution. Briefly, libraries were rescued with M13K07 helper phage and phage particles were then prepared and applied to ELISA plates pre‐coated with purified BACE1 ectodomain. After incubation and washing, the bound phage particles were eluted with 100 mM triethylamine (pH 10.0) and immediately neutralized with 1 M Tris‐HCl (pH 7.5). Alternatively, purified BACE1 ectodomain protein was labeled with Sulfo‐NHS‐SS‐Bio (Pierce), according to the manufacturer’s protocol. Phage particles were pre‐blocked with 1% (w/v) BSA in panning buffer (50 mM Tris‐HCl (pH 7.5), 150 mM NaCl and 0.05% (v/v) Tween‐20), and incubated with 200 nM biotin‐labeled BACE1. Streptavidin‐coated paramagnetic beads (Pierce), pre‐blocked in 1% BSA, were used to capture biotin‐labeled BACE1. After extensive washes, the bound protein and phage particles were eluted by 50 mM DTT. Eluates from both approaches were used to re‐infect exponentially growing TG1 cells for a further round of phage panning. After three consecutive rounds of panning, phages recovered from the second and third rounds were used to infect exponentially growing TG1 cells, which were plated out at dilutions of 10^−4^, 10^−5^, and 10^−6^. Single colonies were picked for further analysis.

### Phage ELISA Screening

Single colonies retrieved from different rounds of phage panning were grown with shaking at 220 rpm in 2 ml 2× YT medium (1.6% (w/v) bacto tryptone, 0.5% (w/v) NaCl, and 1% (w/v) yeast extract in water) supplemented with 100 μg/ml ampicillin and 1% (w/v) glucose in 24‐well plates for 8 h at 37°C. Bacteria in the exponential growth phase were infected using M13K07 helper phages (5 × 10^8^ plaque forming units [pfu] per well) for 20 min at room temperature. After addition of 70 μg/ml of kanamycin to the cells, they were further grown overnight at 37°C, with constant shaking at 220 rpm. The next morning, bacteria were harvested by centrifugation at 1,200g_Av_ for 20 min. Supernatants containing phage particles were tested for binding to BACE1 in an ELISA. Essentially, 96‐well plates (Nunc) were coated with BACE1 ectodomain protein at 100 ng/well overnight at 4°C. Non‐coated wells were used as a control. The plates were blocked with 3% milk (w/v) in PBS for 1 h at RT. After blocking, 100 μl of each phage particle containing supernatant was added to a coated and a non‐coated well and incubated for 2 h at room temperature. Plates were then washed using washing buffer (0.05% (v/v) Tween‐20, PBS) and further incubated with HRP‐conjugated anti‐M13 antibody (GE Healthcare, #27942101) at a 1:3,000 dilution in 3% (w/v) milk in PBS for 1 h at room temperature. Plates were then extensively washed and developed using 0.2 mg/ml ABTS (2,2’‐azino‐bis(3‐ethylbenzothiazoline‐6‐sulfonic acid; Sigma) in 50 mM citric acid, pH 4.0 supplemented with 0.2% (v/v) H_2_O_2_ (Sigma) as a substrate. Signals were read at 405 nm with an ELISA plate reader (2103 EnVision, Perkin Elmer).

### ELISA screening of periplasmic extracts

The expression vector pHEN4 encodes a PelB (pectate lysase) signal sequence at the N‐terminus of the multiple cloning site. Hence, the VHHs encoded by the plasmid are exported to the periplasmic space when expressed in bacterial cultures. To generate periplasmic extract for VHH screening, single colonies, identified by phage ELISA, were inoculated in individual wells of a 24‐well plate, each containing 1 ml Terrific Broth (TB) medium (2.4% (w/v) yeast extract, 2% (w/v) tryptone, 0.4% (v/v) glycerol, 17 mM KH_2_PO_4_, and 72 mM K_2_HPO_4_) supplemented with 100 μg/ml ampicillin. Plates were left at 37°C with shaking at 220 rpm. When the OD600 reached 0.6 in a well, 1 mM IPTG (isopropyl β‐D‐1‐thiogalactopyranoside) was added to the culture to induce protein expression. Bacteria were grown for approximately 16 h at 28°C and were then harvested by centrifugation at 1,200 *g*_Av_ for 20 min. Cell pellets were resuspended in TES solution (200 mM Tris‐HCl (pH 8), 0.5 mM EDTA, 500 mM sucrose) and incubated on ice for 30 min. To release the VHH from the periplasmic space, a mild osmotic shock was given by adding 1.5× volume of TES buffer diluted four times in H_2_O. After incubation on ice for 45 min, the supernatants were cleared by centrifugation at 1,000 *g*_Av_ for 20 min at 4°C and tested by ELISA. As the VHHs were engineered to contain a C‐terminal hemagglutinin (HA) tag, a mouse monoclonal anti‐HA antibody (Covance, MMS‐101P) was used as the detection antibody. An alkaline phosphatase conjugated goat anti‐mouse antibody (Sigma) was used as the secondary antibody. ELISA plates were developed using PNPP (p‐Nitrophenylphosphate; Sigma) as a substrate. Signals were read at 405 nm with an ELISA reader (2103 EnVision, Perkin Elmer).

### Sequence analysis and subcloning of VHHs into a bacterial expression vector

Positive colonies identified by phage ELISA and periplasmic extract ELISA were analyzed by PCR and restriction enzyme digestion (using HinfI and TaqI enzymes), in order to group different sequence patterns. Primers used for colony PCR were forward 5′‐GGACTAGTGCGGCCGCTGGAGACGGTGACCTGGGT‐3′ and reverse 5′‐ TCACACAGGAAACAGCTATGAC‐3′. VHH cDNAs with different restriction enzyme digestion patterns were randomly chosen for sequencing and subcloned into the pHEN6 expression vector, which contains the coding sequence for a C‐terminal hexa‐histidine tag to facilitate purification.

### VHH expression and purification

pHEN6 expression vectors were transformed into WK6 *E. coli* bacteria. Bacteria were inoculated in 1 liter of TB medium supplemented with 100 μg/ml ampicillin and 0.1% (v/v) glucose. Flasks were incubated at 37°C with continuous agitation at 220 rpm. When the OD600 reached 0.75–1.0 in a flask, 1 mM IPTG was added to induce VHH expression. Bacteria were then cultured overnight at 28°C with shaking. The next morning, bacteria were harvested by centrifugation and the periplasmic extract was prepared following the protocol described above. VHHs were purified by affinity chromatography using Ni‐NTA beads (Qiagen) and size exclusion chromatography on Superdex‐75 (GE Healthcare). Protein concentrations were determined by UV absorption at 280 nm, using the theoretical extinction coefficient of the individual VHHs (calculated based on their amino acid content).

### Affinity measurements

The binding affinities of selected VHHs for BACE1 were analyzed by surface plasmon resonance (SPR) spectroscopy, using a BIAcore instrument. For immobilization, 2,500 resonance units (RU) purified BACE1 were coupled to a CM5 chip using amine coupling chemistry (EDC/NHS), according to the manufacturer’s instructions. Binding/regeneration cycles were performed by injection of the VHHs (in a concentration range of 0–500 nM) at 25°C in HBS buffer (10 mM Hepes (pH 7.5), 150 mM NaCl, 3.5 mM EDTA, and 0.005% (v/v) Tween‐20) at a constant flow rate of 30 μl/min. Regeneration of the surface was achieved by injection of 10 mM glycine/HCl (pH 1.5). For affinity measurements at acidic pH, running buffer was replaced by a citrate buffer containing 150 mM NaCl, 3.5 mM EDTA, 0.005% (v/v) Tween‐20 at pH 4.5.

### Bio‐layer interferometry

Biotinylated human or mouse BACE1 was immobilized at 1 μg/ml for 900 s on streptavidin‐coated biosensors (Molecular Devices). The biosensors were washed for 120 s in Kinetic Buffer (Molecular Devices) and dipped into wells containing serial dilutions of VHH‐B9 (1,000 s duration). This association step was followed by dipping the biosensors in Kinetic Buffer to allow dissociation (1,000 s). All steps were performed at 25°C with constant agitation at 1,000 rpm on an OctetRED96 system (Molecular Devices). Binding parameters were calculated using Molecular Devices Analysis 9.0 software, with a 1:1 homogeneous fitting model.

### Production and purification of BACE1:Fc

The construct expressing human BACE1 (amino acids 1–460) fused to the constant region of human IgG1 (hBACE1:Fc) was described previously (Zhou *et al*, 2011). Mutations, including SQD376‐378/WAA (F(SQD/WAA)), EDVATSQDD371‐379/MGAGLNYE (F(BACE2)), EVATSQD371‐378/EGS (ΔF), and GFPLNQSEVLASVG219‐232/GAG (ΔA), were introduced into the original construct. Purification of wild‐type or mutant hBACE1:Fc followed a protocol described previously (Zhou *et al*, 2011). Proteins folded correctly and were active in the MBPC125APPswe cleavage assay. Protein purity was assessed by SDS‐PAGE using a 4–12% Bis‐Tris gel, followed by Coomassie staining. Protein concentration was measured by Bradford assay (Bio‐Rad). Aliquots of the purified protein were snap‐frozen and stored at −80°C.

### *In* *vitro* APP cleavage assay

The assay was performed essentially as described (Zhou *et al*, 2011), using materials provided by Eli Lilly. Briefly, hBACE1:Fc was diluted to 10 nM in reaction buffer (50 mM ammonium acetate [pH 4.6], 1 mg/ml BSA and 1 mM Triton X‐100). The substrate was a fusion protein consisting of maltose binding protein (MBP) and the 125 amino acids from the carboxy terminus of human APP695 containing the Swedish (Sw) mutation (K670M/N671L). Substrate was diluted to 50 nM in reaction buffer. 10 μl test VHHs (5 μM) were incubated with 25 μl substrate and 15 μl hBACE1:Fc at 25°C for 3 h. At the end of the incubation, the reaction mixture was diluted fivefold in “stop” buffer (200 mM Tris (pH 8.0), 6 mg/ml BSA, and 1 mM Triton X‐100). 50 μl of the diluted reaction mixture was then loaded on an ELISA plate precoated with an anti‐MBP capture antibody. The cleavage product MBP‐C26sw was detected using an antibody against a neo‐epitope generated by BACE1‐mediated cleavage. Purified MBP‐C26sw was used to generate a standard curve. The amount of cleavage product reflects the relative activity of BACE1.

### Dot blot analysis of VHH binding to wild‐type and mutant BACE1

200 ng purified wild‐type or mutant hBACE11‐460:Fc (in a final volume of 10 μl) was dotted onto strips of nitrocellulose membrane. After drying, the membrane strips were incubated with blocking buffer (5% (w/v) milk, 0.05% (v/v) Tween‐20, Tris‐buffered saline (TBS: 50 mM Tris‐HCl, 150 mM NaCl; pH 7.6)) for 1 h at room temperature. After blocking, the membrane strips were incubated with primary antibodies, including the mouse monoclonal antibodies 1A11, 5G7, 10B8 (Zhou *et al*, 2011), and VHH‐B9 (all diluted to 1 ug/ml in blocking buffer) for 1 h at room temperature. The membranes were then washed three times in washing buffer (0.05% (v/v) Tween‐20, TBS) (5 min per wash). After washing, the membrane strip incubated with VHH‐B9 was further incubated with an anti‐His antibody (1:2,000 dilution, Biolegend, J099B12) for 1 h at room temperature, followed by three washes, each of 5‐min duration. All membranes were subsequently incubated with HRP‐conjugated goat‐anti‐mouse IgG antibody diluted in blocking buffer (1:10,000 dilution, Novus, NB7539) for 1 h at room temperature. After incubation, the membranes were washed three times in washing buffer and two times in TBS (5 min per wash), before signal was developed using enhanced chemiluminescence (ECL) methods. Signals were captured with a Fuji LAS‐1000 Luminescent Image Analyzer.

### Cell‐based assays (cultured neurons and glia)

Mixed primary brain neurons were derived from C57Bl/6J mice at embryonic day 14 (E14), following a previously described protocol (Zhou *et al*, 2011). Glial cultures were established from C57Bl/6J mice at post‐natal day 3 (P3), according to a previously described protocol (Voytyuk *et al*, 2018). Once confluent, glia from each dish were frozen in 1 ml freezing medium (90% (v/v) FBS and 10% (v/v) DMSO) to establish a frozen stock. Cultures were reactivated when needed, using standard culturing protocols.

#### Experiments with neurons using addition of purified nanobodies

Neurons were cultured in 6 cm dishes (Nunc) and maintained in neurobasal medium (Gibco) supplemented with B27 (Gibco). Neurons were transduced with SFV expressing WT human APP695 (Annaert *et al*, 1999) after 3 days in culture. Two hours after transduction, neurons were treated with test VHH, diluted in fresh media for 12 h.

#### Experiments with neurons or glia using application of AAV‐VHH

AAV1 vectors encoding VHH‐B9 or GFP were generated by the tri‐transfection method using HEK293T cells (as described in Fripont *et al*, 2019) and applied to cells in culture. The pan‐BACE inhibitor, Compound J (CpJ) (Esterházy *et al*, 2011), was used as a positive control at a final concentration of 10 nM. DMSO alone was used as the vehicle control. The final amount of vector applied was approximately 3.75 × 10^11^ vector genomes (vg).

AAV vectors were applied to neurons in culture at day 3 in vitro (DIV). 3 days post‐AAV application, CpJ or DMSO were added according to the following treatment protocol: (i) AAV‐VHH‐B9 + Vehicle, AAV‐GFP + Vehicle (negative control) and AAV‐GFP + Compound J (positive control). Media was collected 36 hours later and concentrated using centrifugal concentrators (30 kDa molecular weight cut‐off, Millipore). Cells were collected and homogenized in 5 volumes of ice‐cold PBS, supplemented with protease (Roche) and phosphatase (Sigma) inhibitors. Lysates were centrifuged in an Ultra Optima TLX (Beckman) at 14,000 *g*_Av_ at 4°C for 15 min. Supernatants were collected, snap‐frozen using liquid N_2_, and stored at −80°C.

AAV vectors were added to glia when the cells had reached confluency. 3 days post‐AAV application, CpJ or DMSO were added as described above. Media and cells were collected 36 h later and processed as described.

### Western blot

The amount of total protein in both culture media and cell extracts was measured using the BCA Method (Thermo Fisher Scientific). Samples were prepared by mixing with Laemmli loading buffer and heating at 75°C for 10 min. Samples were then loaded onto 10% Bis‐Tris SDS‐PAGE gels (20 μg/lane) and separated by electrophoresis in NuPAGE MES SDS running buffer, except for samples obtained from glial cultures, which were loaded onto 4–12% Bis‐Tris SDS‐PAGE gels (20 μg/lane) and separated by electrophoresis in NuPAGE MOPS SDS running buffer. Immunoblotting was carried out using standard tank blotting techniques. The membranes were incubated with blocking buffer (5% (w/v) milk, 0.05% (v/v) Tween‐20, TBS) for 1 h at room temperature. Primary antibodies in blocking buffer were added overnight at 4°C. Primary antibodies used were WO2 for total Aβ (1:1,000 dilution, The Genetics Company, AB4‐10); 6E10 for sAPPα (1:1,000 dilution, Signet Laboratories, #9320‐02); anti‐APPβ (1:1,000 dilution, Covance, #SIG‐39138); custom B63 antibody for full‐length and carboxy terminal fragments of APP (1:5,000 dilution) (Annaert *et al*, 2001); anti‐β‐actin (1:3,000 dilution, Sigma‐Aldrich, A5441), anti‐DNER (1:1,000 dilution, R&D Systems, AF2254), anti‐llama IgG H&L (1:1,000 dilution, HRP, AB112786), anti‐VCAM (1:1,000 dilution, Thermo Fisher Scientific, PA5‐47029), anti‐SEZ6 (1:1,000, R&D Systems, AF5598), anti‐cMyc (9E10) (1:1,000 dilution, Abcam, ab32), and anti‐GFP (1:1,000 dilution, Sigma, 11814460001). The membranes were then washed three times in 0.05% (v/v) Tween‐20, TBS (7 min per wash) and subsequently incubated with corresponding secondary antibody in blocking buffer for 1 h at room temperature. Secondary antibodies used were HRP‐conjugated goat anti‐mouse IgG antibody (1:10,000 dilution, Novus, NB7539), HRP‐conjugated goat‐anti‐rabbit IgG antibody (1:10,000 dilution, Novus, NBP2‐30348H), goat polyclonal anti‐mouse IgG‐HRP conjugate (1:3,000 dilution, Biorad, 170‐6516), goat polyclonal anti‐rabbit IgG‐HRP conjugate (1:3,000 dilution, Biorad, 170‐6515), or rabbit polyclonal anti‐goat IgG‐HRP conjugate (1:2,000 dilution, DAKO, P0449). After the incubation, the membranes were washed three times in 0.05% (v/v) Tween‐20, TBS and two times in TBS (5 min per wash) before signal was developed with ECL reagents. Signals were captured and analyzed with a Fuji LAS‐1000 Luminescent Image Analyzer.

### AAV vector production (for systemic administration)

AAV‐based vectors were designed around a standard single stranded (ss) AAV2 expression cassette. The cDNA for the anti‐BACE1 VHH‐B9 was modified to contain an N‐terminal BACE1 signal peptide and a C‐terminal cMyc tag. The cDNA sequence encoding VHH‐B9 (or GFP for control) was then cloned into the expression cassette, which also contained a ubiquitously active CAG promoter, a woodchuck post‐transcriptional regulatory element (WPRE), and a bovine growth hormone poly(A) sequence (pA). The plasmid pAAV‐Rep2/PHP.B capsid was cloned in house, using online data as a guide (Deverman *et al*, 2016). Final production and quality control of vectors were performed by Vigene Biosciences. Further details about packaging and purification strategies can be found on the company’s website (http://www.vigenebio.com).

### Systemic administration of AAV vectors

A dose of 1 × 10^12^ vector genomes (vg)/mouse of AAV was administered to animals using tail vein injection. To perform the injection safely and avoid injury and stress, each mouse was placed inside a restraining device. Dilation of the tail vein was stimulated by friction with a cotton pad and a 70% ethanol solution. A standard vector volume of 100 μl per animal was administered using an insulin syringe with ultra‐fine needle (29 Gauge, Becton Dickinson). In the event of bleeding, a clean cotton pad was applied at the end of the procedure. Each animal was then returned to its original cage and monitored for 15 min for adverse effects. Allocation of the animals to treatment groups was performed randomly.

### Sociability and preference for social novelty task

Social approach and memory were tested in a three‐chamber setup constructed from plexiglass (Nadler *et al*, 2004; Naert *et al*, 2011). The setup consisted of a central chamber (38 × 11 × 30 cm, length × width × height) with two adjacent chambers (11 × 11 × 30 cm, length × width × height) connected by a wall with fifty‐two 8 mm holes to allow exchange of olfactory, visual, and auditory signals. The test mouse was placed in the central chamber. Following a 5‐min habituation phase, a strange (novel) mouse (S1) was placed in one of the adjacent chambers and the preference of the habituated mouse to approach S1 over the empty chamber was recorded for 6 min. After 6 min, a second stranger (S2) was placed in the opposing empty chamber, and social memory for S2 was analyzed for a further 6 min. Animals were tracked using an overhead webcam and ANY‐maze tracking software (Stölting). Time sniffing (head within 5 cm of the separation wall) was compared between groups.

### Morris water maze

Spatial learning and reference memory were tested in a hidden platform protocol in the Morris water maze (Callaerts‐Vegh *et al*, 2006). Animals were trained for 10 days to locate a hidden platform using distal spatial cues. Reference memory was assessed in interspersed probe trials, where the platform was removed and time spent in virtual zones close to the target platform was assessed. Animals were tracked in a round pool (150 cm diameter) filled with opacified water (26 ± 1°C) using the Ethovision system. Non‐performers (animals not actively engaged in swimming activities) were excluded from subsequent analysis using pre‐established criteria (non‐performance: swim velocity < 5 cm/s for longer than 35% of total time).

### Transcardial perfusion and brain extraction for post‐mortem analysis

Animals were anesthetized via intraperitoneal injection of 100 μl Pentobarbital sodium (Dolethal) solution. Upon lack of response to a pain stimulus (toe pinch‐response), transcardial perfusion was performed with 10 ml of ice‐cold PBS. The brain was then extracted and dissected along the interhemispheric fissure. Each right hemisphere was post‐fixed for 24 h in 4% (w/v) PFA solution and then stored in 0.02% (w/v) NaN_3_‐PBS solution at 4°C before use in immunohistochemistry‐based experiments. Each left hemisphere was dissected at the stereoscopic microscope, to isolate the hippocampus and cortex, which were then snap‐frozen in liquid nitrogen and stored at −80°C prior to Aβ extraction and measurement using ELISA.

### Immunohistochemistry

Post‐fixed brain hemispheres were sliced at the LEICA VT1000S vibrating microtome to obtain 50 µm thick sagittal sections. The brain sections were put into separate wells of a 24‐well plate and rinsed in a solution of 50 mM Tris‐Cl, 150 mM NaCl (TBS, pH 7.6) for 10 min. Permeabilization and blocking of non‐specific binding sites was achieved by incubation in 500 μl of blocking solution (10% [v/v] Normal Donkey Serum, Abcam; 1% [v/v] Triton‐X100, Sigma‐Aldrich; TBS) for 2 h at room temperature. The brain sections were then incubated in 500 μl of primary antibody solution overnight at 4°C. On the following day, the brain sections were rinsed six times in TBS. Each washing step was carried out for 10 min. The sections were then incubated for 2 h, at room temperature, in 500 μl of secondary antibody solution. Finally, the sections were rinsed six times with TBS and mounted on SuperFrost Plus Adhesion glass slides (Thermo Fisher Scientific) with Fluromount‐G without addition of DAPI (Thermo Fisher Scientific), unless otherwise specified. The glass slides were stored at 4°C, protected from light to prevent photobleaching.

#### Staining of VHH‐B9

Brain distribution of VHH‐B9 was assessed by staining against the cMyc tag fused to the VHH. Primary and secondary antibody solutions were prepared by adding rabbit anti‐cMyc (1:500 dilution, Abcam, #ab9106) and donkey anti‐rabbit Alexa 488 (1:200 dilution, Thermo Fisher Scientific, #A21206) to blocking solution, respectively.

#### Staining of Aβ plaques, GFAP, and Iba1

Primary antibody solution was prepared by adding mouse anti‐human Aβ 82E1 (1:300 dilution, Tecan, #JP10323), guinea pig anti‐GFAP (1:1,000 dilution, Synaptic Systems, #173 004), or goat anti‐Iba1 (1:1,000 dilution, Abcam, #ab5076) to blocking solution. Secondary antibody solution was prepared by adding donkey anti‐mouse Cy3 (1:400 dilution, Jackson Immuno, #715‐165‐150), donkey anti‐guinea pig Alexa 488 (1:400 dilution, Jackson Immuno, #706‐545‐148), or donkey anti‐goat Alexa 647 (1:400 dilution, Jackson Immuno, #705‐605‐147) to blocking solution.

Aβ plaques were co‐stained with Thioflavin‐S to facilitate counting and analysis. The brain sections were incubated in a 0.05% (w/v) Thioflavin‐S/50% ethanol solution for 5 min at room temperature. The sections were then rinsed in 70% ethanol and 95% ethanol, with each rinse lasting 5 min. To ensure thorough ethanol removal, the sections were then immersed three times in sterile H_2_0 (B. Braun Medical Inc.) (3 × 5 min).

#### Co‐staining of Aβ plaques and Synaptophysin

Aβ plaques were stained as previously described. The presynaptic marker synaptophysin was stained by the addition of rabbit anti‐synaptophysin 1 (1:1,000, Synaptic Systems, #101 203) and donkey anti‐rabbit AlexaFluor 488 (1:200, Thermo Fisher Scientific, #A21206) to primary and secondary antibody solutions, respectively.

#### Co‐staining of VHH and BACE1

For BACE1 staining, antigen retrieval was performed, prior to blocking, by boiling sections in citrate buffer (10 mM citric acid, 0.05% (v/v) Tween‐20; pH 6.0 NaOH) followed by three washes in PBS, each of 5‐min duration. Sections were then processed using our standard protocol. Primary antibodies used for staining were rat anti‐cMyc (1:400 dilution, Bio‐Rad, #MCA1929) and rabbit anti‐BACE1 (1:150 dilution, Cell Signaling, D10E5). Secondary antibody solution contained donkey anti‐rat Alexa 488 (1:200 dilution, Jackson Immuno, #712‐545‐150) and donkey anti‐rabbit Cy3 (1:200 dilution, Jackson Immuno, #711‐165‐152). The brain sections were mounted onto microscopy slides, using Fluoromount G containing DAPI (SouthernBiotech).

### Imaging and analysis

Brain sections for counting of Aβ plaques, and reactive astrocytes and microglia, were imaged using a Leica SP8 confocal laser scanning microscope, equipped with a HC PL APO 20x/NA0.75 objective (Leica, #15506517). A Leica DM5500 epifluorescence microscope with a HC PL APO 10x/NA0.45 objective (Leica, #506411) was used to take the mosaic image in Fig 2C and the single fields of view in Fig EV3C–F. Quantification of Aβ plaque number and reactive astro‐ and microgliosis was carried out in a semi‐automatized fashion with the aid of ImageJ/Fiji software.

### ELISA

Sandwich ELISAs were performed using a standard protocol, with minor modifications. Nunc maxisorp flat‐bottom 96‐well plates (VWR, #735‐0079) were used. Wells were incubated with 100 μl of coating buffer (10 mM Tris‐HCl, 10 mM NaCl, 10 mM NaN_3_; pH 8.5), containing either capture antibody or purified recombinant VHH‐B9. This incubation was performed overnight at 4°C on a tilting table. The next day, coating buffer was discarded and each well rinsed five times with 150 μl of DPBS (Gibco), supplemented with 0.05% (v/v) Tween20 (Sigma‐Aldrich). Each washing step was carried out for 10 min. Non‐specific binding was prevented by blocking with 100 μl DPBS supplemented with 0.1% (w/v) casein from bovine milk (PBS‐casein, Sigma‐Aldrich) for 4 h at room temperature.

#### Detection of anti‐VHH‐B9 antibodies

Blood collection was performed pre‐mortem via cardiac puncture. Serum was then separated from the whole blood by centrifugation at 2,000 *g*_Av_ for 10 min at 4°C. For detection of VHH‐B9‐specific antibodies, Nunc MaxiSorp plates (Thermo Fisher Scientific) were coated with purified VHH‐B9 (75 ng total protein) and stored overnight at 4°C. Mouse anti‐camelid IgG2/3 (heavy chain) antibody (50 μl, 50 μg/ml; Merck Millipore #MAC131) was used as a positive control; PBS was used as a negative control. Test (serum) samples (50 µl final volume) were obtained from three individual mice per experimental cohort (1:10,000 serum dilution in PBS) (Appendix Table S1). Samples were incubated overnight at 4°C. Horse anti‐mouse IgG HRP‐conjugated (50 µl final volume, 1:5,000 dilution, Cell Signaling Technology, #7076S) was used as the detection antibody. Absorbance was measured at 450 nm in a Perkin Elmer EnVision 2103 Multilabel reader.

#### Quantification of Aβ_1‐40_ and Aβ_1‐42_

Soluble and insoluble fractions of Aβ were extracted from cortex and hippocampus, as previously described (Latif‐Hernandez *et al*, 2020). The total protein content of the samples containing the soluble and insoluble Aβ fractions was determined using a modified Lowry–Peterson assay (Lowry *et al*, 1951; Peterson, 1977). Monoclonal antibodies JRFcAβ40/28 and JRFcAβ42/26 (1.5 μg/ml in 100 μl of coating buffer) were used for capture. These antibodies recognize the C‐terminus of Aβ species terminating at amino acids 40 or 42, respectively. HRP‐conjugated JRFAβN/25 antibody (1:6,000 in PBS‐casein), which recognizes the first seven N‐terminal amino acids of human Aβ, was used for detection. In these experiments, detection antibody was mixed with sample prior to adding to the capture plate. The plate was then left overnight at 4°C degrees on a tilting table. The following day, the plate was rinsed five times with 0.05% (v/v) Tween 20/PBS, before colorimetric‐based detection (as described above). Synthetic human Aβ_1‐40_ and Aβ_1‐42_ peptides were used to generate standard curves. Aβ quantification was performed using 30 μl of test sample, at a protein concentration of 3 μg/μl (cortical samples) or 1.5 μg/μl (hippocampal samples), respectively. If necessary, samples were further diluted to ensure measured absorbance values fell within the linear range of the standard curve. Antibodies were kindly provided by Johnson and Johnson.

### Multielectrode array (MEA) LTP recordings

LTP recordings were done in 13‐ to 16‐month old mice. For tissue preparation, mice were anesthetized with isoflurane and decapitated. Brains were rapidly removed and 300 μm thick parasagittal brain slices prepared using a Leica VT1200 vibratome. Slicing was performed in a sucrose‐based cutting solution (ACSF) containing (in mM) 87 NaCl, 2.5 KCl, 1.25 NaH_2_PO_4_, 10 glucose, 25 NaHCO_3_, 0.5 CaCl_2_, 7 MgCl_2_, 75 sucrose, 1 kynurenic acid, 5 ascorbic acid, 3 pyruvic acid (pH 7.4 (HCl); 95% O_2_/5% CO_2_ gas). Slices were allowed to recover at 34°C for 35 min, and then maintained at room temperature in the same solution for at least 30 min before using. For recordings, slices were placed onto a multielectrode array (MEA 2100, Multi Channel Systems) and continuously perfused with artificial cerebrospinal fluid (aCSF) solution containing (in mM): 119 NaCl, 2.5 KCl, 1 NaH_2_PO_4_, 11 glucose, 26 NaHCO_3_, 4 MgCl_2_ and 4 CaCl_2_ (pH 7.4 (HCl); 95% O_2_/5% CO_2_ gas; 34°C). Field excitatory post‐synaptic potentials (fEPSPs) were recorded from Schaffer collateral‐CA1 synapses by stimulating and recording from the appropriate (visually identified) electrodes. Input–output curves were recorded for each slice by applying single‐stimuli ranging from 500 to 2,750 mV with 250 mV increments. A stimulus strength that corresponds to 35% of the maximal response in the input–output curve was used for recordings. For long‐term potentiation (LTP) experiments, stable fEPSPs were recorded for 30 min to establish a baseline. Next, we applied three high‐frequency trains (100 stimuli; 100 Hz) with 5‐min intervals. Subsequently, post‐LTP fEPSPs were measured every 5 min (average of three consecutive stimuli at 15 sec intervals) for 55 min. Recordings were processed and analyzed using Multi Channel Experimenter software (Multi Channel Systems).

### Data collection and statistical analysis

In the initial proof‐of‐concept study, the investigator performing the experiments was aware of which treatment groups animals had been assigned to. In the longitudinal study, all experiments were performed by an investigator blinded to treatment group allocation. Normality in sample distribution was verified with the Shapiro–Wilk test. Statistical analysis was chosen accordingly and is indicated in the corresponding figure legend.

## Author contributions

**Marika Marino:** Formal analysis; Investigation; Visualization; Writing ‐ original draft; Project administration; Writing ‐ review & editing. **Lujia Zhou:** Formal analysis; Investigation; Visualization. **Melvin Y Rincon:** Formal analysis; Investigation; Visualization. **Zsuzsanna Callaerts‐Vegh:** Formal analysis; Supervision. **Jens Verhaert:** Investigation. **Jérôme Wahis:** Formal analysis; Visualization. **Eline Creemers:** Investigation. **Lidia Yshii:** Investigation. **Keimpe Wierda:** Formal analysis; Investigation; Visualization. **Takashi Saito:** Resources. **Catherine Marneffe:** Investigation. **Iryna Voytyuk:** Investigation. **Yessica Wouters:** Investigation. **Maarten Dewilde:** Formal analysis; Investigation; Visualization. **Sandra I Duqué:** Formal analysis; Investigation; Visualization. **Cécile Vincke:** Formal analysis; Investigation; Methodology. **Yona Levites:** Resources; Methodology. **Todd Golde:** Resources; Supervision; Methodology. **Takaomi C Saido:** Resources. **Serge Muyldermans:** Resources; Supervision; Investigation; Methodology. **Adrian Liston:** Formal analysis; Supervision; Investigation; Methodology. **Bart De Strooper:** Resources; Formal analysis; Supervision; Visualization; Methodology. **Matthew G Holt:** Conceptualization; Resources; Data curation; Formal analysis; Supervision; Funding acquisition; Visualization; Writing ‐ original draft; Project administration; Writing ‐ review & editing.

In addition to the CRediT author contributions listed above, the contributions in detail are:

MGH conceived the project. LZ, CV, SM, YW, MD, and BDS generated and characterized the anti‐BACE1 VHH. TS generated and characterized the *App^NL‐G‐F^* mouse line, with input on data analysis and interpretation by TCS. MM performed all the long‐term mouse experiments, including behavioral testing under the supervision of ZCV. JV provided technical support to MM at all stages of the experiments. EC and KW performed the electrophysiology and analyzed data, with subsequent data processing by JW. MYR, SID, YL, and TEG were involved in the initial pilot experiments testing AAV‐mediated VHH‐B9 expression and activity in AD mouse models. LZ, CM, and IV performed the tissue culture experiments. LY and AL devised and performed the ELISA measurements for anti‐VHH. MM, ZCV, LZ, BDS, and MGH analyzed the data. MM and MGH wrote the manuscript with input from all authors.

## Disclosure and competing interests statement

Johnson and Johnson provided antibodies used in this study. Eli Lilly provided reagents used in the peptide cleavage experiments. However, neither company played a role in the design and execution of the study, or the interpretation of results. BDS has acted as a consultant for Janssen Pharmaceutica and Remynd NV. AL and MGH are co‐founders of Aila Biotech, whose main products are built around AAV technology described in this manuscript. The remaining authors declare no conflict of interest.

## For more information

https://www.alzforum.org is an information resource to disseminate relevant news related to the treatment of Alzheimer’s disease and related disorders, and promote networking between researchers.

https://www.alz.org is a link to the Alzheimer's Association website. The mission of the Alzheimer’s Association is to accelerate global research and provide treatment options for Alzheimer's and other dementias, prioritizing quality care and patient support.

http://www.genetherapynet.com is an information resource for patients and professionals interested in basic and clinical research in gene and cell therapy. Gene Therapy Net provides an overview of the different international regulations and guidelines associated with clinical gene therapy trials.

https://www.fda.gov/vaccines‐blood‐biologics/cellular‐gene‐therapy‐products/what‐gene‐therapy is a link to the gene therapy section of the US Food and Drug Administration (FDA) website, which provides basic information on gene therapy, in a manner accessible to the general public.

## Supporting information



Appendix

Expanded View Figures PDF

## Data Availability

This study includes no data deposited in external repositories.
