# Supplementary material for: AAV‐mediated delivery of an anti‐BACE1 VHH alleviates pathology in an Alzheimer's disease model
Source: EMBO Mol Med. 2022 Mar 30;14(4):e09824. doi: 10.15252/emmm.201809824 (PMC8988209; doi:10.15252/emmm.201809824)
Supplement: Supplementary file 1 — Appendix [file EMMM-14-e09824-s001.pdf]

AAV-mediated delivery of an anti-BACE1 VHH alleviates pathology in an Alzheimer's disease model

Marika Marino, Lujia Zhou, Melvin Y. Rincon, Zsuzsanna Callaerts-Vegh, Jens Verhaert, Jérôme Wahis, Eline Creemers, Lidia Yshii, Keimpe Wierda, Takashi Saito, Catherine Marneffe, Iryna Voytyuk, Yessica Wouters, Maarten Dewilde, Sandra I. Duqué, Cécile Vincke, Yona Levites, Todd E. Golde, Takaomi C Saido, Serge Muyldermans, Adrian Liston, Bart De Strooper, Matthew G. Holt.

Appendix Table of Contents:

- Appendix Figure S1
- Appendix Figure S2
- Appendix Table S1

APPENDIX FIGURE LEGENDS

Appendix Figure S1: Inhibition of BACE1 activity by VHH-B9 appears to show substrate specificity.

(A) Primary cultured neurons were transduced with an AAV vector driving the expression of VHH-B9 (or GFP as a control). The non-selective BACE1 and BACE2 inhibitor Compound J (CpJ) was used as a positive control. VHH-B9 inhibited APP cleavage, as seen by a decrease in sAPPβ production. However, no decrease in SEZ6 shedding was observed. In contrast CpJ successfully inhibited both APP and SEZ6 shedding. Anti-cMyc tag and anti-llama IgG were used for VHH detection.

(B) Primary cultured glia were transduced with an AAV vector driving the expression of VHH-B9, or treated with CpJ. As expected from its binding profile, VHH-B9 had no effect on the cleavage of the BACE2 substrates DNER and VCAM1. CpJ effectively blocked shedding of both substrates. n=3 cultures for each analysis. Actin was used as a loading control in all experiments. Representative blot images are shown.

Appendix Figure S2: Detection of VHH-B9 specific IgG in serum.

Detection of anti-VHH-B9 IgG was performed via ELISA. No immune response was detectable in either *App*$^{NL\text{-}G\text{-}F}$ (KI) or C57BL/6J (WT) mice that received AVV-VHH-B9. Uninjected C57BL/6J mice and *App*$^{NL\text{-}G\text{-}F}$ or C57BL/6J mice receiving AVV-VHH-B9 were used as control cohorts. Data points represent serum samples from individual animals. Positive control; purified recombinant VHH-B9; negative control DPBS.

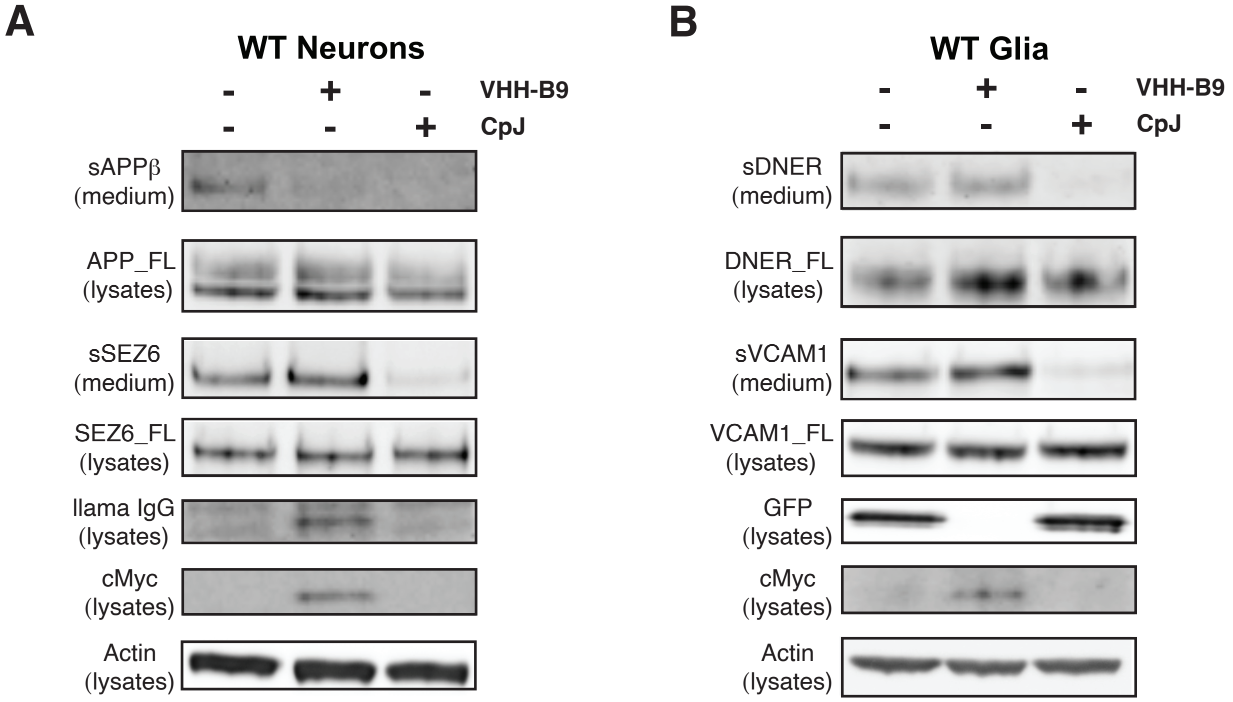

Appendix Figure S1_Marino et al, 2022

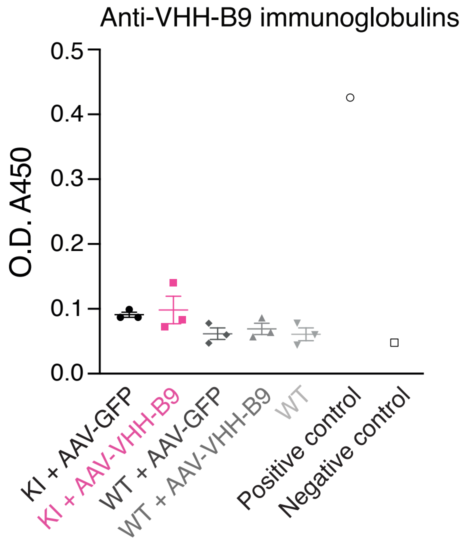

Appendix Figure S2_Marino et al, 2022

Animal cohorts of short-term study (EV3)

Genotype	Sex	Birth (dd/mm/yyyy)	Injection (dd/mm/yyyy)	Route	AAV-PHP.B	Dose (vg/mouse)	Euthanasia (dd/mm/yyyy)	Observations	Experiment
App $^{NL-G-F}$	F	1/9/2018	15/10/2018	Systemic (Tail vein)	GFP	$1x10^{12}$	7/1/2019	/	Aβ ELISA
App $^{NL-G-F}$	F	1/9/2018	15/10/2018	Systemic (Tail vein)	GFP	$1x10^{12}$	7/1/2019	/	Aβ ELISA
App $^{NL-G-F}$	F	1/9/2018	15/10/2018	Systemic (Tail vein)	GFP	$1x10^{12}$	7/1/2019	/	Aβ ELISA
App $^{NL-G-F}$	F	1/9/2018	15/10/2018	Systemic (Tail vein)	GFP	$1x10^{12}$	7/1/2019	/	Aβ ELISA
App $^{NL-G-F}$	M	10/9/2018	22/10/2018	Systemic (Tail vein)	GFP	$1x10^{12}$	14/1/2019	/	Aβ ELISA
App $^{NL-G-F}$	M	1/9/2018	15/10/2018	Systemic (Tail vein)	VHH-B9	$1x10^{12}$	7/1/2019	/	Aβ ELISA
App $^{NL-G-F}$	M	1/9/2018	15/10/2018	Systemic (Tail vein)	VHH-B9	$1x10^{12}$	7/1/2019	/	Aβ ELISA
App $^{NL-G-F}$	M	1/9/2018	15/10/2018	Systemic (Tail vein)	VHH-B9	$1x10^{12}$	7/1/2019	/	Aβ ELISA
App $^{NL-G-F}$	M	1/9/2018	15/10/2018	Systemic (Tail vein)	VHH-B9	$1x10^{12}$	7/1/2019	/	Aβ ELISA
App $^{NL-G-F}$	F	10/9/2018	22/10/2018	Systemic (Tail vein)	VHH-B9	$1x10^{12}$	14/1/2019	/	Aβ ELISA
App $^{NL-G-F}$	M	3/11/2018	19/12/2018	Systemic (Tail vein)	VHH-B9	$1x10^{12}$	11/3/2019	/	Aβ ELISA
App $^{NL-G-F}$	M	3/11/2018	19/12/2018	Systemic (Tail vein)	VHH-B9	$1x10^{12}$	11/3/2019	/	Aβ ELISA
App $^{NL-G-F}$	M	3/11/2018	19/12/2018	Systemic (Tail vein)	VHH-B9	$1x10^{12}$	11/3/2019	/	Aβ ELISA

Animal cohorts of longitudinal study

Genotype	Sex	Birth (dd/mm/yyyy)	Injection (dd/mm/yyyy)	Route	AAV-PHP.B	Dose (vg/mouse)	Euthanasia (dd/mm/yyyy)	Observations	Experiment
App $^{NL-G-F}$	M	2/12/2020	14/01/2020	Systemic (Tail vein)	GFP	$1x10^{12}$	18/1/2021	/	IHC/ELISA*
App $^{NL-G-F}$	M	2/12/2020	14/01/2020	Systemic (Tail vein)	GFP	$1x10^{12}$	18/1/2021	/	IHC/ELISA*
App $^{NL-G-F}$	M	2/12/2020	14/01/2020	Systemic (Tail vein)	GFP	$1x10^{12}$	18/1/2021	/	IHC/ELISA*
App $^{NL-G-F}$	M	2/12/2020	14/01/2020	Systemic (Tail vein)	GFP	$1x10^{12}$	18/1/2021	/	Aβ ELISA
App $^{NL-G-F}$	M	2/12/2020	14/01/2020	Systemic (Tail vein)	GFP	$1x10^{12}$	18/1/2021	/	Aβ ELISA
App $^{NL-G-F}$	M	2/12/2020	14/01/2020	Systemic (Tail vein)	GFP	$1x10^{12}$	18/1/2021	/	Aβ ELISA
App $^{NL-G-F}$	M	2/12/2020	14/01/2020	Systemic (Tail vein)	GFP	$1x10^{12}$	18/1/2021	/	Aβ ELISA
App $^{NL-G-F}$	M	2/12/2020	14/01/2020	Systemic (Tail vein)	GFP	$1x10^{12}$	18/1/2021	/	Aβ ELISA
App $^{NL-G-F}$	M	2/12/2020	14/01/2020	Systemic (Tail vein)	GFP	$1x10^{12}$	18/1/2021	/	Aβ ELISA
App $^{NL-G-F}$	M	2/12/2020	14/01/2020	Systemic (Tail vein)	GFP	$1x10^{12}$	18/1/2021	/	Aβ ELISA
App $^{NL-G-F}$	M	2/12/2020	14/01/2020	Systemic (Tail vein)	GFP	$1x10^{12}$	18/1/2021	/	Aβ ELISA
App $^{NL-G-F}$	M	2/12/2020	14/01/2020	Systemic (Tail vein)	GFP	$1x10^{12}$	16/02/2021	/	Electrophysiology
App $^{NL-G-F}$	M	2/12/2020	14/01/2020	Systemic (Tail vein)	GFP	$1x10^{12}$	10/03/2021	/	Electrophysiology
App $^{NL-G-F}$	M	2/12/2020	14/01/2020	Systemic (Tail vein)	GFP	$1x10^{12}$	18/03/2021	/	Electrophysiology
App $^{NL-G-F}$	M	2/12/2020	14/01/2020	Systemic (Tail vein)	GFP	$1x10^{12}$	01/04/2021	/	Electrophysiology
App $^{NL-G-F}$	M	2/12/2020	14/01/2020	Systemic (Tail vein)	GFP	$1x10^{12}$	09/04/2021	/	Electrophysiology
App $^{NL-G-F}$	M	2/12/2020	14/01/2020	Systemic (Tail vein)	VHH-B9	$1x10^{12}$	18/1/2021	/	IHC/ELISA*
App $^{NL-G-F}$	M	2/12/2020	14/01/2020	Systemic (Tail vein)	VHH-B9	$1x10^{12}$	18/1/2021	/	IHC/ELISA*
App $^{NL-G-F}$	M	2/12/2020	14/01/2020	Systemic (Tail vein)	VHH-B9	$1x10^{12}$	18/1/2021	/	IHC/ELISA*
App $^{NL-G-F}$	M	2/12/2020	14/01/2020	Systemic (Tail vein)	VHH-B9	$1x10^{12}$	18/1/2021	/	Aβ ELISA
App $^{NL-G-F}$	M	2/12/2020	14/01/2020	Systemic (Tail vein)	VHH-B9	$1x10^{12}$	18/1/2021	/	Aβ ELISA
App $^{NL-G-F}$	M	2/12/2020	14/01/2020	Systemic (Tail vein)	VHH-B9	$1x10^{12}$	18/1/2021	/	Aβ ELISA
App $^{NL-G-F}$	M	2/12/2020	14/01/2020	Systemic (Tail vein)	VHH-B9	$1x10^{12}$	18/1/2021	/	Aβ ELISA
App $^{NL-G-F}$	M	2/12/2020	14/01/2020	Systemic (Tail vein)	VHH-B9	$1x10^{12}$	18/1/2021	/	Aβ ELISA
App $^{NL-G-F}$	M	2/12/2020	14/01/2020	Systemic (Tail vein)	VHH-B9	$1x10^{12}$	18/1/2021	/	Aβ ELISA
App $^{NL-G-F}$	M	2/12/2020	14/01/2020	Systemic (Tail vein)	VHH-B9	$1x10^{12}$	18/1/2021	/	Aβ ELISA
App $^{NL-G-F}$	M	2/12/2020	14/01/2020	Systemic (Tail vein)	VHH-B9	$1x10^{12}$	18/1/2021	/	Aβ ELISA
App $^{NL-G-F}$	M	2/12/2020	14/01/2020	Systemic (Tail vein)	VHH-B9	$1x10^{12}$	18/1/2021	/	Aβ ELISA
App $^{NL-G-F}$	M	2/12/2020	14/01/2020	Systemic (Tail vein)	VHH-B9	$1x10^{12}$	17/02/2021	/	Electrophysiology
App $^{NL-G-F}$	M	2/12/2020	14/01/2020	Systemic (Tail vein)	VHH-B9	$1x10^{12}$	11/03/2021	/	Electrophysiology
App $^{NL-G-F}$	M	2/12/2020	14/01/2020	Systemic (Tail vein)	VHH-B9	$1x10^{12}$	19/03/2021	/	Electrophysiology
App $^{NL-G-F}$	M	2/12/2020	14/01/2020	Systemic (Tail vein)	VHH-B9	$1x10^{12}$	02/04/2021	/	Electrophysiology
App $^{NL-G-F}$	M	2/12/2020	14/01/2020	Systemic (Tail vein)	VHH-B9	$1x10^{12}$	12/04/2021	/	Electrophysiology
C57Bl/6J	M	5/12/2020	14/01/2020	Systemic (Tail vein)	GFP	$1x10^{12}$	19/1/2021	Sick	/
C57Bl/6J	M	5/12/2020	14/01/2020	Systemic (Tail vein)	GFP	$1x10^{12}$	19/1/2021	/	IHC/ELISA*
C57Bl/6J	M	5/12/2020	14/01/2020	Systemic (Tail vein)	GFP	$1x10^{12}$	19/1/2021	/	IHC/ELISA*
C57Bl/6J	M	5/12/2020	14/01/2020	Systemic (Tail vein)	GFP	$1x10^{12}$	19/1/2021	/	IHC/ELISA*
C57Bl/6J	M	5/12/2020	14/01/2020	Systemic (Tail vein)	GFP	$1x10^{12}$	19/1/2021	/	Aβ ELISA
C57Bl/6J	M	5/12/2020	14/01/2020	Systemic (Tail vein)	GFP	$1x10^{12}$	19/1/2021	/	Aβ ELISA
C57Bl/6J	M	5/12/2020	14/01/2020	Systemic (Tail vein)	GFP	$1x10^{12}$	19/1/2021	/	Aβ ELISA
C57Bl/6J	M	5/12/2020	14/01/2020	Systemic (Tail vein)	GFP	$1x10^{12}$	19/1/2021	/	Aβ ELISA
C57Bl/6J	M	5/12/2020	14/01/2020	Systemic (Tail vein)	GFP	$1x10^{12}$	19/1/2021	/	Aβ ELISA
C57Bl/6J	M	5/12/2020	14/01/2020	Systemic (Tail vein)	GFP	$1x10^{12}$	19/1/2021	/	Aβ ELISA
C57Bl/6J	M	5/12/2020	14/01/2020	Systemic (Tail vein)	GFP	$1x10^{12}$	19/1/2021	/	Aβ ELISA
C57Bl/6J	M	5/12/2020	14/01/2020	Systemic (Tail vein)	GFP	$1x10^{12}$	19/1/2021	/	Aβ ELISA
C57Bl/6J	M	5/12/2020	14/01/2020	Systemic (Tail vein)	GFP	$1x10^{12}$	19/1/2021	/	Aβ ELISA
C57Bl/6J	M	5/12/2020	14/01/2020	Systemic (Tail vein)	GFP	$1x10^{12}$	29/01/2021	/	Electrophysiology
C57Bl/6J	M	5/12/2020	14/01/2020	Systemic (Tail vein)	GFP	$1x10^{12}$	04/03/2021	/	Electrophysiology
C57Bl/6J	M	5/12/2020	14/01/2020	Systemic (Tail vein)	GFP	$1x10^{12}$	15/03/2021	/	Electrophysiology
C57Bl/6J	M	5/12/2020	14/01/2020	Systemic (Tail vein)	GFP	$1x10^{12}$	30/03/2021	/	Electrophysiology
C57Bl/6J	M	5/12/2020	14/01/2020	Systemic (Tail vein)	GFP	$1x10^{12}$	07/04/2021	/	Electrophysiology
C57Bl/6J	M	5/12/2020	14/01/2020	Systemic (Tail vein)	VHH-B9	$1x10^{12}$	20/1/2021	/	IHC/ELISA*
C57Bl/6J	M	5/12/2020	14/01/2020	Systemic (Tail vein)	VHH-B9	$1x10^{12}$	20/1/2021	/	IHC/ELISA*
C57Bl/6J	M	5/12/2020	14/01/2020	Systemic (Tail vein)	VHH-B9	$1x10^{12}$	20/1/2021	/	IHC/ELISA*
C57Bl/6J	M	5/12/2020	14/01/2020	Systemic (Tail vein)	VHH-B9	$1x10^{12}$	20/1/2021	/	Aβ ELISA
C57Bl/6J	M	5/12/2020	14/01/2020	Systemic (Tail vein)	VHH-B9	$1x10^{12}$	20/1/2021	/	Aβ ELISA
C57Bl/6J	M	5/12/2020	14/01/2020	Systemic (Tail vein)	VHH-B9	$1x10^{12}$	20/1/2021	/	Aβ ELISA
C57Bl/6J	M	5/12/2020	14/01/2020	Systemic (Tail vein)	VHH-B9	$1x10^{12}$	20/1/2021	/	Aβ ELISA
C57Bl/6J	M	5/12/2020	14/01/2020	Systemic (Tail vein)	VHH-B9	$1x10^{12}$	20/1/2021	/	Aβ ELISA
C57Bl/6J	M	5/12/2020	14/01/2020	Systemic (Tail vein)	VHH-B9	$1x10^{12}$	20/1/2021	/	Aβ ELISA

C57Bl/6J	M	5/12/2020	14/01/2020	Systemic (Tail vein)	VHH-B9	$1x10^{12}$	20/1/2021	/	Aβ ELISA
C57Bl/6J	M	5/12/2020	14/01/2020	Systemic (Tail vein)	VHH-B9	$1x10^{12}$	20/1/2021	/	Aβ ELISA
C57Bl/6J	M	5/12/2020	14/01/2020	Systemic (Tail vein)	VHH-B9	$1x10^{12}$	20/1/2021	/	Aβ ELISA
C57Bl/6J	M	5/12/2020	14/01/2020	Systemic (Tail vein)	VHH-B9	$1x10^{12}$	20/1/2021	/	Aβ ELISA
C57Bl/6J	M	5/12/2020	14/01/2020	Systemic (Tail vein)	VHH-B9	$1x10^{12}$	15/02/2021	/	Electrophysiology
C57Bl/6J	M	5/12/2020	14/01/2020	Systemic (Tail vein)	VHH-B9	$1x10^{12}$	05/03/2021	/	Electrophysiology
C57Bl/6J	M	5/12/2020	14/01/2020	Systemic (Tail vein)	VHH-B9	$1x10^{12}$	16/03/2021	/	Electrophysiology
C57Bl/6J	M	5/12/2020	14/01/2020	Systemic (Tail vein)	VHH-B9	$1x10^{12}$	31/03/2021	/	Electrophysiology
C57Bl/6J	M	5/12/2020	15/01/2020	Systemic (Tail vein)	VHH-B9	$1x10^{12}$	08/04/2021	/	Electrophysiology
C57Bl/6J	M	5/12/2020	16/01/2020	Not injected	/	/	25/1/2021	Sick	/
C57Bl/6J	M	5/12/2020	17/01/2020	Not injected	/	/	25/1/2021	/	IHC/ELISA*
C57Bl/6J	M	5/12/2020	18/01/2020	Not injected	/	/	25/1/2021	/	IHC/ELISA*
C57Bl/6J	M	5/12/2020	19/01/2020	Not injected	/	/	25/1/2021	/	IHC/ELISA*
C57Bl/6J	M	5/12/2020	20/01/2020	Not injected	/	/	25/1/2021	/	Aβ ELISA
C57Bl/6J	M	5/12/2020	21/01/2020	Not injected	/	/	25/1/2021	/	Aβ ELISA
C57Bl/6J	M	5/12/2020	22/01/2020	Not injected	/	/	25/1/2021	/	Aβ ELISA
C57Bl/6J	M	5/12/2020	23/01/2020	Not injected	/	/	25/1/2021	/	Aβ ELISA
C57Bl/6J	M	5/12/2020	24/01/2020	Not injected	/	/	25/1/2021	/	Aβ ELISA
C57Bl/6J	M	5/12/2020	25/01/2020	Not injected	/	/	25/1/2021	/	Aβ ELISA
C57Bl/6J	M	5/12/2020	26/01/2020	Not injected	/	/	25/1/2021	/	Aβ ELISA
C57Bl/6J	M	5/12/2020	27/01/2020	Not injected	/	/	25/1/2021	/	Aβ ELISA
C57Bl/6J	M	5/12/2020	28/01/2020	Not injected	/	/	25/1/2021	/	Aβ ELISA
C57Bl/6J	M	5/12/2020	29/01/2020	Not injected	/	/	28/01/2021	/	Electrophysiology
C57Bl/6J	M	5/12/2020	30/01/2020	Not injected	/	/	09/03/2021	/	Electrophysiology
C57Bl/6J	M	5/12/2020	31/01/2020	Not injected	/	/	17/03/2021	/	Electrophysiology
C57Bl/6J	M	5/12/2020	01/02/2020	Not injected	/	/	29/03/2021	/	Electrophysiology
C57Bl/6J	M	5/12/2020	02/02/2020	Not injected	/	/	06/04/2021	/	Electrophysiology

* = ELISA for Aβ quantification and detection of anti-VHH-B9 IgG in serum

Appendix Table S1. Description of animals used in short-term (EV3) and long-term studies, including information on animal genotype, date of birth, AAV type used, route of administration, AAV cargo (VHH or GFP), vector dose, date of euthanasia, follow up experiments performed (IHC, ELISA, electrophysiology) and any relevant observations.
