# Supplementary material for: AAV‐mediated delivery of an anti‐BACE1 VHH alleviates pathology in an Alzheimer's disease model
Source: EMBO Mol Med. 2022 Mar 30;14(4):e09824. doi: 10.15252/emmm.201809824 (PMC8988209; doi:10.15252/emmm.201809824)
Supplement: Supplementary file 2 — Expanded View Figures PDF [file EMMM-14-e09824-s003.pdf]

Expanded View Figures

A

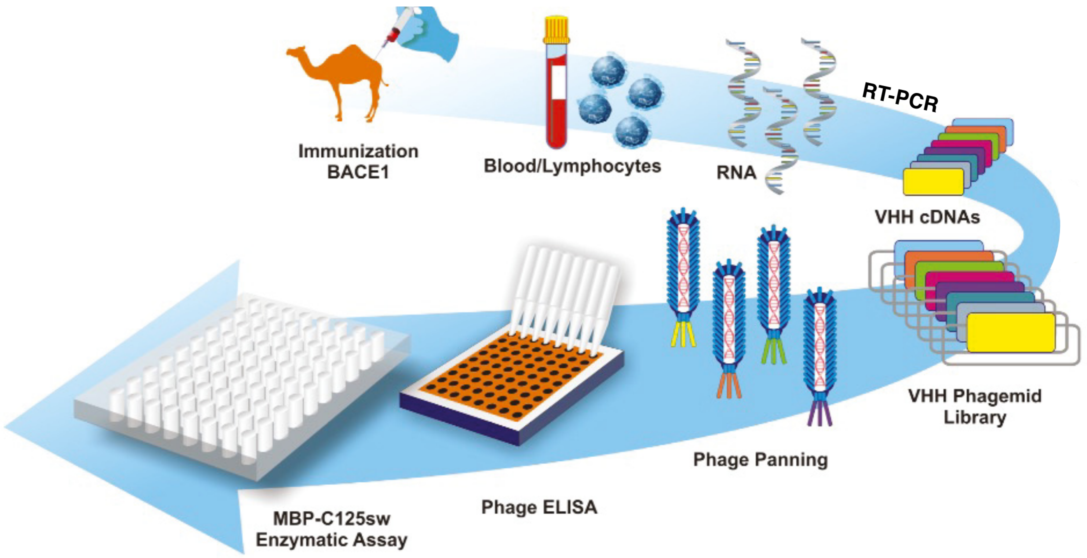

B

```
           <  - - Framework 1  - - > <  H1  > <  Framework 2 >
VHH-B9     DVQLQESGGGSVQAGGSLRLSCAAS EYTYGYCS MGWYRQAPGKERELVS 49
VHH-10C4   QVQLQESGGGSVQAGGFLRLSCAAS GYTYSTCS MAWYRQAPGKERELVS 49
VHH-4A2    QVQLQESGGGLVQPGGSLRLSCAAS GFTFETQY MTWVRQAPGKGPEYVS 49

           <- H2 -> < - - - -  Framework 3 - - - - - - - - >
VHH-B9     TIT-SDGS TSYVD.SVKGRFTISQDNAKNTVYLQMNSLKPEDTAKYYC  95
VHH-10C4   SIR-NDGS TAYAD.SVKGRFTISQDNAKNTVYLQMNSLKPEDTAMYYC  95
VHH-4A2    SINSGGTI KYYANSSVKGRFTISRDNAKNTLYLQMNNLRPEDTAIYYC  95

           < - H3 loop -  > <Framework4>
VHH-B9     YTKTCANK..LGAKFIS WGQGTQVTVSS  121
VHH-10C4   NIRIVGPGGTCSIYAPY WGEGTQVTVSS  124
VHH-4A2    QLGQWA....GVGAASS RGQGTQVTVSS  121
```

Figure EV1. Anti-BACE1 VHH production and selection.

A Schematic summarizing the production procedure. A dromedary and a llama were immunized with recombinant human BACE1 ectodomain (amino acids 46–460). Blood lymphocytes from the immunized animals were collected for RNA extraction. cDNA was prepared and the variable fragments of heavy chain only IgGs were amplified by RT-PCR and purified using agarose gel electrophoresis. cDNAs encoding VHH were cloned into the pHEN4 phagemid vector and phage libraries were prepared. Three rounds of consecutive phage panning were performed to enrich phage particles that bound recombinant BACE1 in an ELISA assay. Binding to BACE1 was confirmed in a phage ELISA. Finally, BACE1 binding VHHs were purified with initial testing of inhibitory activity using an *in vitro* APP cleavage assay (MBP-C125sw enzymatic assay).

B Protein sequences for anti-BACE1 VHH identified as having inhibitory activity in the *in vitro* APP cleavage assay. Sequences are aligned, with framework and CDR regions indicated.

 © 2022 The Authors

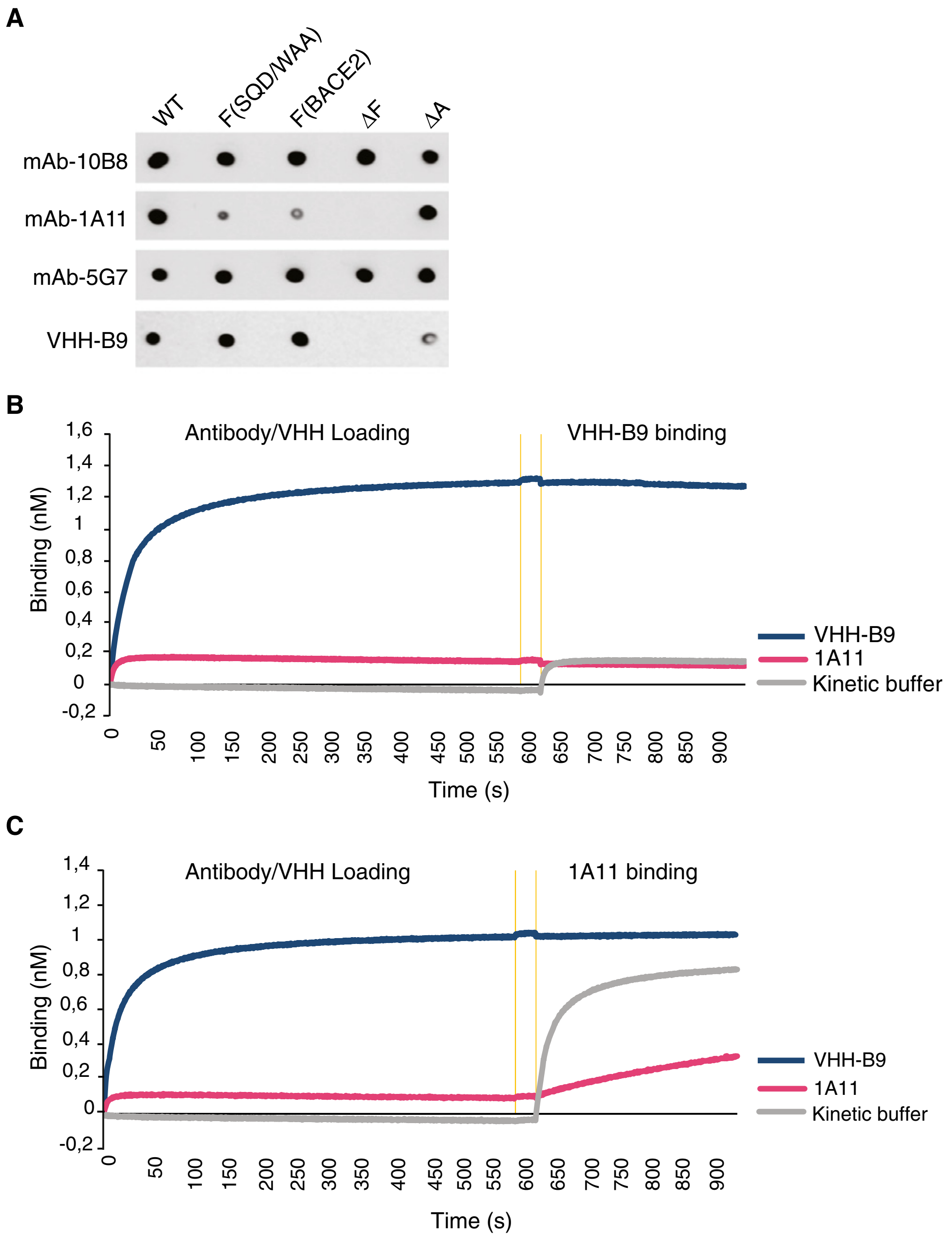

Figure EV2. VHH-B9 binding is specific to BACE1 with an epitope identical or overlapping the one targeted by the 1A11 anti-BACE1 monoclonal antibody.

A VHH-B9 binds to a unique exosite on BACE1. Wild-type (WT) BACE1 ectodomain (1–460) or various mutants, including S376Q377D378/WAA (F(SQD/WAAA)), EDVATSQDD371-379/MGAGLNYE (F(BACE2)), EVATSQD371-378/EGS (ΔF), and GFPLNQSEVLASVG219-232/GAG (ΔA), were purified from cultures of HEK293 cells. 200 ng purified protein was dotted onto a nitrocellulose membrane and probed with the indicated antibodies. Monoclonal antibodies 10B8, 5G7, and 1A11 were used as positive controls. 10B8 and 5G7 recognize all forms of BACE1 tested, indicating that the mutants were properly folded. 1A11 does not recognize BACE1 with mutations in loop F but recognizes Helix A mutants, as well as WT BACE1, as previously reported (Zhou *et al*, 2011). VHH-B9 only weakly recognized ΔLoop F and ΔHelix A mutants, but recognized other Loop F mutants, S376Q377D378/WAA and EDVATSQDD371-379/MGAGLNYE. Helix A is a structure adjacent to Loop F, flanking the active-site cleft of BACE1. This suggests that VHH-B9 binds via a conformational epitope engaging Helix A and Loop F, although direct binding to Loop F may not necessarily be needed. Binding to these structural elements, unique to BACE1, likely explains the specific inhibition seen with VHH-B9.

B, C VHH-B9 and monoclonal antibody 1A11 compete for binding to BACE1. Epitope binning was done by biolayer interferometry (Octet RED96; Molecular Devices). Biotinylated BACE1 was bound to streptavidin sensor tips. Afterwards, BACE1-loaded sensors were dipped in 1A11, VHH-B9, or kinetic buffer for 600 s to allow binding. After a 30-s equilibration in kinetic buffer (orange vertical lines), tips were dipped in VHH-B9 or 1A11. (B) No additional binding of VHH-B9 was observed. (C) A low level of 1A11 binding was observed. This residual binding is most probably due to the displacement of VHH-B9 by 1A11, suggesting that these two antibodies bind to the same or adjacent epitopes.

© 2022 The Authors

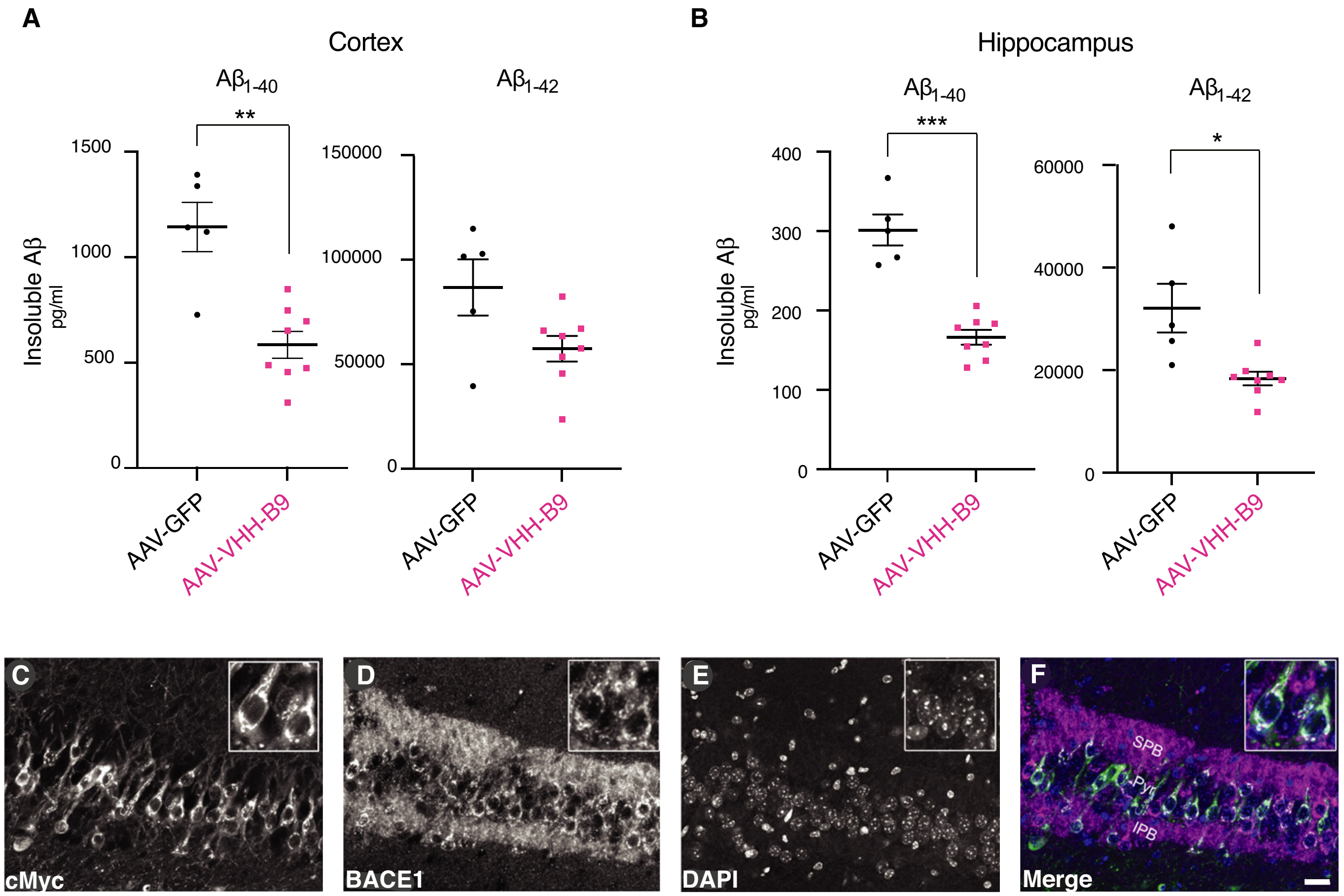

Figure EV3. Proof-of-concept experiment for AAV-mediated VHH-B9 production and activity *in vivo*.

A, B *App*$^{NL\text{-}G\text{-}F}$ mice were systemically injected with 1×10^{12} vg of AAV-VHH-B9 ($n = 8$) or the control vector AAV-GFP ($n = 5$). Cortical samples (A) and hippocampi (B) were collected 12-week post-injection for analysis. Protein was extracted and used for quantification of insoluble Aβ_{1-40} and Aβ_{1-42} levels by ELISA. VHH-B9 expression led to a significant decrease in both insoluble Aβ_{1-40} and Aβ_{1-42} levels. Each dot represents the mean between duplicate Aβ measurements in each individual mouse. Statistical analyses were performed with an unpaired *t*-test with Welch's correction. (A) $^{**}P = 0.004$; (B) $^{*}P = 0.04$, $^{***}P = 0.0008$.

C–F VHH-B9 co-localized with BACE1 in hippocampal neurons following AAV-mediated delivery. Representative images of coronal brain sections are shown for staining against BACE1 (magenta) and cMyc (green). Images show colocalization of VHH-B9 with BACE1 in the pyramidal cell layer (Pyr), as well as in the suprapyramidal blade (SPB) and infrapyramidal blade (IPB) of the mossy fibers. The diffuse staining patterns suggest that VHH-B9 is engaged with BACE1 in internal structures, such as early endosomes. Scale bar, 25 µm.

© 2022 The Authors

Figure EV4. Long-term BACE1 inhibition does not influence sociability and social recognition memory in C57Bl/6J cohorts and spatial learning in the Morris Water Maze in *App*$^{NL-G-F}$ mice.

A Sociability and social recognition memory in C57Bl/6J cohorts. In a standard test for sociability (see also Fig 2), C57Bl/6J cohorts show normal sociability, preferring to interact with a novel mouse [S1] rather than an empty chamber [E]. Social recognition memory (preference for a novel mouse in S2 over S1) was observed in controls. Data are presented as violin plots. Median and quartiles (thick and thin white lines, respectively) indicate spread of data ($n = 18$ per group). Sociability ***$P = 0.0005$, ****$P < 0.0001$; Preference for social novelty *$P = 0.004$, **$P = 0.006$ (two-way ANOVA with post-hoc multiple comparison using Šídák correction).

B, C Spatial learning in the Morris water maze in control groups. Mice were trained in a Morris Water Maze apparatus for 10 days (one trial per day) over two working weeks; the break in plots between days 5 and 6 indicates a pause in testing over a weekend. (B) C57BL/6J animals learned to rapidly locate a submerged platform in the pool over successive trials as indicated by a reduction in the total distance swum by the animal (path length) and directionality of the swim pattern (distance to platform from initial start point). No difference was observed between non-injected (wild type: WT) animals and those receiving 1×10^{12} vg of either AAV-VHH-B9 or AAV-GFP (C) Reference memory was assessed in 'probe' trials, by removing the submerged platform from the tank. No quadrant preference was observed after the first 5 days of training. However, all animals showed a robust preference for the quadrant previously containing the platform (T: target) during a second 'probe' trial, conducted after 10 days training. (A1/A2: adjacent quadrants; O: opposite quadrant). Data are presented as means ± SEM (WT+AAV-B9 $n = 18$; WT+AAV-GFP $n = 17$; WT $n = 17$). Statistical analyses were performed using a one sample t-test. As all C57BL/6J cohorts learned equally well, only AAV-injected wild types were used in comparisons with *App*$^{NL-G-F}$ (KI) mice. *$P = 0.01$–0.02; **$P = 0.006$ versus chance (One sample T-Test).

D Spatial learning in the Morris Water Maze for *App*$^{NL-G-F}$ (colored symbols) and C57BL/6J control mice (gray symbols). *App*$^{NL-G-F}$ performed similar to their respective C57Bl/6J controls. No difference was observed between genotypes after AAV-VHH-B9 (left) or AAV-GFP (right) administration. Data are presented as means ± SEMs (KI+AAV-VHH B9 $n = 13$; KI+AAV-GFP $n = 12$; WT+AAV-VHH-B9 $n = 16$; WT+AAV-GFP $n = 14$).

E–G Reference memory in *App*$^{NL-G-F}$ mice was assessed in interspersed probe trials. Time spent in the various pool quadrants was recorded as a measure for reference memory. (E) During the first probe trial (after 5 days trainings), both genotypes showed a similar quadrant preference, irrespective of AAV treatment paradigm. Preference for "T" was not above that dictated by chance (25%) (dotted line in the graph). (F) During the second probe trial (day 11), both C57Bl/6J cohorts (AAV-VHH-B9 or AAV-GFP) showed a statistically significant preference for "T," which was not detected in *App*$^{NL-G-F}$ mice. (G) *App*$^{NL-G-F}$ mice (injected with either AAV-VHH-B9 or AAV-GFP) showed a clear preference for "T" following an additional week of swim training, indicating that long-term expression of VHH-B9 does not significantly impact spatial learning and reference memory. Data are presented as means ± SEMs (Probe 1 and 2: KI+AAV-VHH-B9 $n = 17$; KI+AAV-GFP $n = 16$; WT+AAV-VHH-B9 $n = 18$; WT+AAV-GFP $n = 17$. Probe 3: KI+AAV-VHH-B9 $n = 16$; KI+AAV-GFP $n = 12$). ***$P = 0.0004$–0.0008 versus chance (One sample T-Test)

© 2022 The Authors

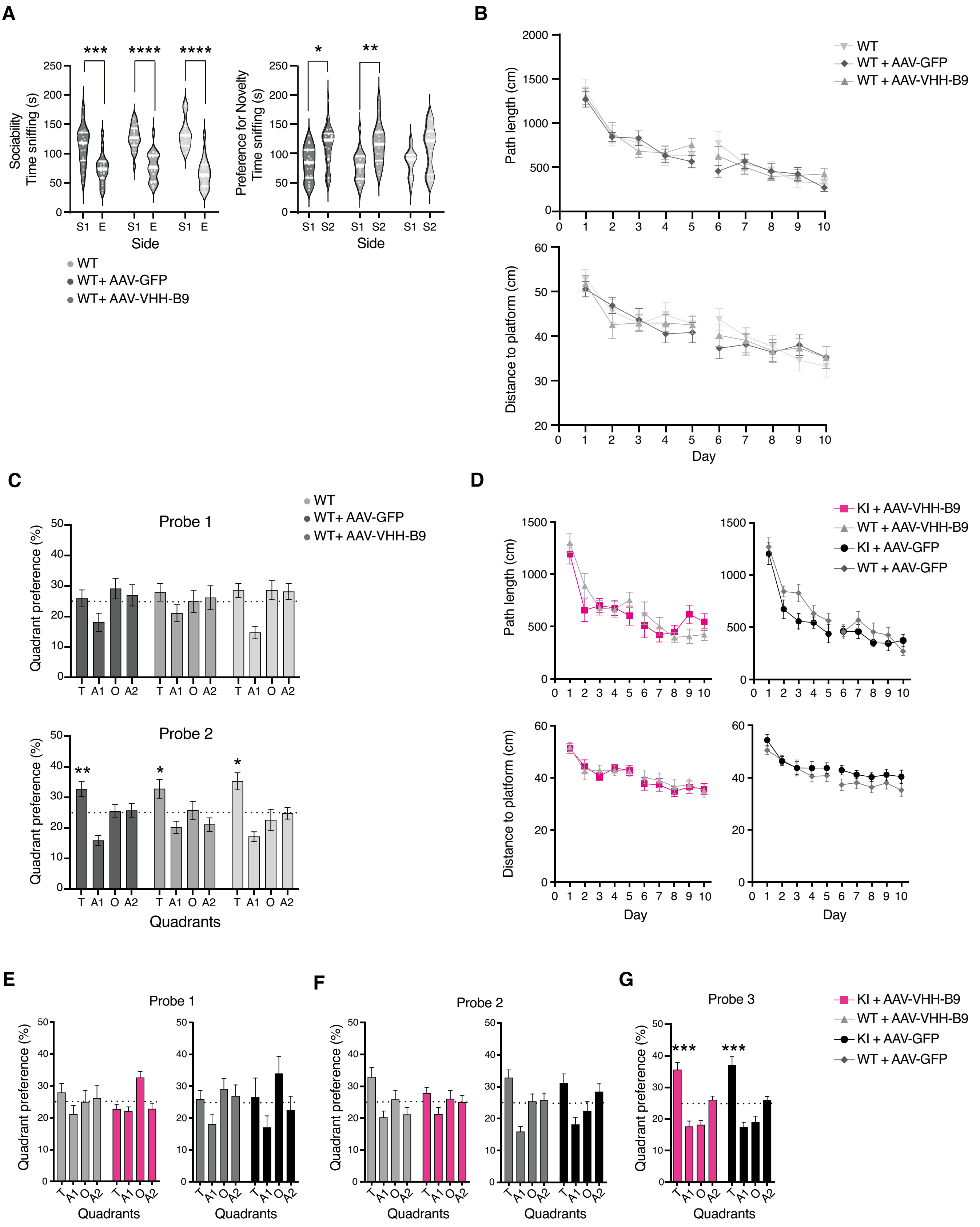

Figure EV4.

© 2022 The Authors

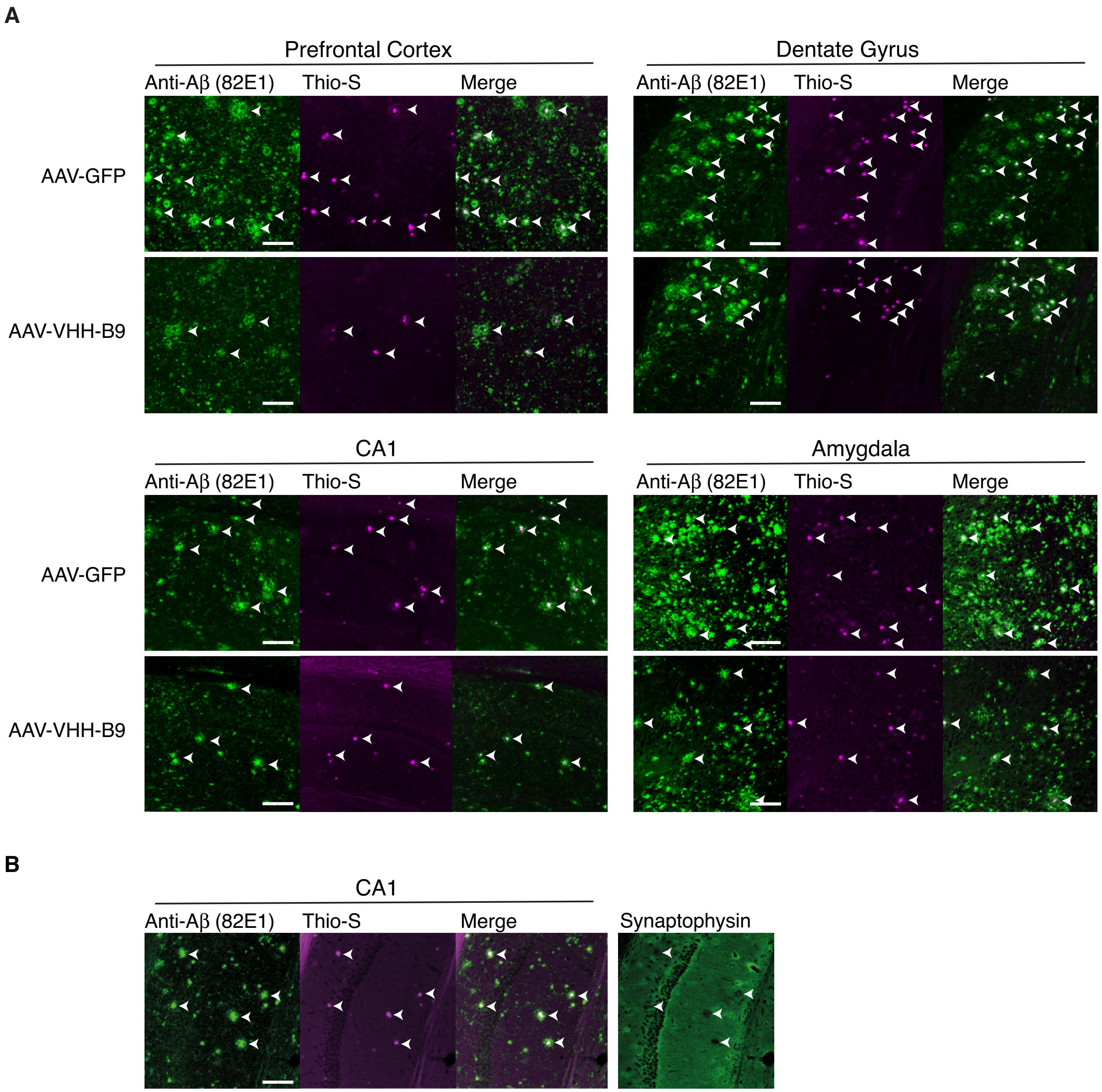

Figure EV5. Amyloid plaque deposition in *App*$^{NL-G-F}$ mice correlates with synaptic loss and is reduced by AAV-VHH-B9.

A AAV-VHH-B9 or AAV-GFP were systemically injected into *App*$^{NL-G-F}$ mice at a dose of 1×10^{12} vg, when mice were 6 weeks of age. Brains were recovered approximately 12-month post-injection, following behavioral testing. Immunostaining with anti-N-Terminal Aβ (82E1) (green) and co-staining with Thioflavin-S (magenta) reveal Aβ plaques (arrow heads) in the regions indicated. Individual channels were used to produce the merge images, which also appear in Fig 3A.

B Antibody staining against the synaptic vesicle marker synaptophysin (green) reveals loss of synaptic terminals in the immediate proximity of amyloid plaques (arrow heads, marked by 82E1 and Thioflavin S staining) in the hippocampal CA1 region.

Data information: Scale bars, 100 μm.

© 2022 The Authors
