# Supplementary material for: AAV‐mediated delivery of an anti‐BACE1 VHH alleviates pathology in an Alzheimer's disease model
Source: EMBO Mol Med. 2022 Mar 30;14(4):e09824. doi: 10.15252/emmm.201809824 (PMC8988209; doi:10.15252/emmm.201809824)
Supplement: Supplementary file 3 [file EMMM-14-e09824-s002.pdf]

EMBO Molecular Medicine

AAV-mediated delivery of an anti-BACE1 VHH alleviates pathology in an Alzheimer's disease model

Marika Marino, Lujia Zhou, Melvin Rincon, Zsuzsanna Callaerts-Vegh, Jens Verhaert, Jérôme Wahis, Eline Creemers, Lidia Yshii, Keimpe Wierda, Takashi Saito, Catherine Marneffe, Iryna Voytyuk, Yessica Wouters, Maarten Dewilde, Sandra Duqué, Cécile Vincke, Yona Levites, Todd Golde, Takaomi Saido, Serge Muyldermans, Adrian Liston, Bart De Strooper, and Matthew Holt
DOI: 10.15252/emmm.201809824

Corresponding author(s): Matthew Holt (Matthew.Holt@kuleuven.be)

Review Timeline:

Submission Date:	15th Jul 19
Editorial Decision:	6th Sep 19
Revision Received:	6th Nov 21
Editorial Decision:	20th Dec 21
Revision Received:	11th Feb 22
Accepted:	14th Feb 22

Editor: Celine Carret , Jingyi Hou

Transaction Report:

(Note: With the exception of the correction of typographical or spelling errors that could be a source of ambiguity, letters and reports are not edited. Depending on transfer agreements, referee reports obtained elsewhere may or may not be included in this compilation. Referee reports are anonymous unless the Referee chooses to sign their reports.)

6th Sep 2019

Dear Dr. Holt,

Thank you for the submission of your manuscript to EMBO Molecular Medicine. We have now heard back from the three referees whom we asked to evaluate your manuscript.

As you will see, the referees find the data to be of interest. Referee 1 is the most critical and suggests that behaviour and pathology should be measured to strengthen the findings (an assessment further supported by referee 3). Referee 2 requested an extended discussion, some clarifications and together with referee 1, to add data to improve conclusiveness.

Given the balance of these evaluations, we feel that we can consider a revision of your manuscript if you can address the issues that have been raised in a manner that will satisfy the referees. Please note that it is EMBO Molecular Medicine policy to allow only a single round of revision and that, as acceptance or rejection of the manuscript will depend on another round of review, your responses should be as complete as possible.

EMBO Molecular Medicine has a "scooping protection" policy, whereby similar findings that are published by others during review or revision are not a criterion for rejection. Should you decide to submit a revised version, I do ask that you get in touch after three months if you have not completed it, to update us on the status.

Please also contact us as soon as possible if similar work is published elsewhere. If other work is published we may not be able to extend the revision period beyond three months.

Please read below for important editorial formatting and consult our author's guidelines for proper formatting of your revised article for EMBO Molecular Medicine.

I look forward to receiving your revised manuscript.

Yours sincerely,

Celine Carret

Celine Carret, PhD
Senior Editor
EMBO Molecular Medicine

*** Instructions to submit your revised manuscript ***

** PLEASE NOTE ** As part of the EMBO Publications transparent editorial process initiative (see our Editorial at https://www.embopress.org/doi/pdf/10.1002/emmm.201000094), EMBO Molecular Medicine will publish online a Review Process File to accompany accepted manuscripts.

In the event of acceptance, this file will be published in conjunction with your paper and will include the anonymous referee reports, your point-by-point response and all pertinent correspondence relating to the manuscript. If you do NOT want this file to be published, please inform the editorial office at contact@embomolmed.org.

To submit your manuscript, please follow this link:

Link Not Available

Please do not share this URL as it will give anyone who clicks it access to your account.

When submitting your revised manuscript, please include:

1) a .doc formatted version of the manuscript text (including Figure legends and tables). Please make sure that the changes are highlighted to be clearly visible to referees and editors alike.

2) separate figure files*

3) supplemental information as Expanded View and/or Appendix. Please carefully check the authors guidelines for formatting Expanded view and Appendix figures and tables at https://www.embopress.org/page/journal/17574684/authorguide#expandedview

4) a letter INCLUDING the reviewers' reports and your detailed responses to their comments (as Word file)

Also, and to save some time should your paper be accepted, please read below for additional information regarding some features of our research articles:

5) The paper explained: EMBO Molecular Medicine articles are accompanied by a summary of the articles to emphasize the major findings in the paper and their medical implications for the non-specialist reader. Please provide a draft summary of your article highlighting
- the medical issue you are addressing,
- the results obtained and
- their clinical impact.

This may be edited to ensure that readers understand the significance and context of the research. Please refer to any of our published articles for an example.

6) For more information: There is space at the end of each article to list relevant web links for further consultation by our readers. Could you identify some relevant ones and provide such information as well? Some examples are patient associations, relevant databases, OMIM/proteins/genes links, author's websites, etc...

7) Author contributions: the contribution of every author must be detailed in a separate section (before the acknowledgments).

8) EMBO Molecular Medicine now requires a complete author checklist (https://www.embopress.org/page/journal/17574684/authorguide) to be submitted with all revised manuscripts. Please use the checklist as a guideline for the sort of information we need WITHIN the manuscript as well as in the checklist. This is particularly important for animal reporting, antibody dilutions (missing) and exact p-values and n that should be indicated instead of a range.

9) Every published paper now includes a 'Synopsis' to further enhance discoverability. Synopses are displayed on the journal webpage and are freely accessible to all readers. They include a short stand first (maximum of 300 characters, including space) as well as 2-5 one sentence bullet points that summarise the paper. Please write the bullet points to summarise the key NEW findings. They should be designed to be complementary to the abstract - i.e. not repeat the same text. We encourage inclusion of key acronyms and quantitative information (maximum of 30 words / bullet point). Please use the passive voice. Please attach these in a separate file or send them by email, we will incorporate them accordingly.

You are also welcome to suggest a striking image or visual abstract to illustrate your article. If you do please provide a jpeg file 550 px-wide x 400-px high.

10) A Conflict of Interest statement should be provided in the main text

11) Please note that we now mandate that all corresponding authors list an ORCID digital identifier. This takes <90 seconds to complete. We encourage all authors to supply an ORCID identifier, which will be linked to their name for unambiguous name identification.

Currently, our records indicate that there is no ORCID associated with your account.

Please click the link below to provide an ORCID:
Link Not Available

12) The system will prompt you to fill in your funding and payment information. This will allow Wiley to send you a quote for the article processing charge (APC) in case of acceptance. This quote takes into account any reduction or fee waivers that you may be eligible for. Authors do not need to pay any fees before their manuscript is accepted and transferred to our publisher.

*Additional important information regarding Figures

Each figure should be given in a separate file and should have the following resolution:
Graphs 800-1,200 DPI
Photos 400-800 DPI
Colour (only CMYK) 300-400 DPI"

Figures are not edited by the production team. All lettering should be the same size and style; figure panels should be indicated by capital letters (A, B, C etc). Gridlines are not allowed except for log plots. Figures should be numbered in the order of their appearance in the text with Arabic numerals. Each Figure must have a separate legend and a caption is needed for each panel.

*Additional important information regarding figures and illustrations can be found at http://bit.ly/EMBOPressFigurePreparationGuideline

***** Reviewer's comments *****

Referee #1 (Remarks for Author):

In this manuscript titled "AAV mediated delivery of a novel anti-BACE1 VHH reduces Abeta in an Alzheimer's disease mouse model", Melvin Y. Rincon et al develop a novel VHH (VHH-B9) to inhibit BACE1 activity in vitro and propose a gene transfer strategy to deliver this VHH directly into CNS by means of using AAV as a vector. In vivo injections of AAV encoded VHH-B9 demonstrated reductions in amyloid beta levels in mice model of cerebral amyloidosis. Similar approach using AAV delivered nanobody in different murine model has been tested (Verhelle A., et al., Human Molecular Genetics, 2017). The main weakness of this study is the lack of evidences for therapeutic benefits in the treated animals. More comprehensive analyses will strengthen the story.

Major points:
1. Lack of data to show the improvements in pathology and cognitive function in treated animals.
2. The authors did not check for long term persistence of VHH-B9 expression in mice brain. Mice were sacked 3 weeks post injection. For therapeutic purposes, considering the route of administration is intracranial, the timelines are much longer, and repeated administration is inconvenient and at times not feasible, monitoring VHH-B9 levels at later time points is important to conclude that there is lack of clearance.
3. Did the authors observe any physical manifestations of the reduction in A 1-40 and A 1-42 in mice brain like reduction in neuroinflammation or hemorrhages which are typically seen in APP Dutch mice?

Minor points:
1. Why did the authors select AAV1 as the preferred serotype for in vivo administration over some other serotypes like AAV9 or PHP.B that have been demonstrated in the past to have higher transduction efficiency for brain than AAV1?
2. The author should examine for any immune responses against either the AAV or VHH-B9. They can conduct simple ELISAs to look for antibodies against the transgene.
3. The most important data presented in this manuscript is Figure 3E and 3F. However, the authors claimed for a significant reduction of A 1-40 and A 1-42 in animals without any statistical analysis shown in the data figure. This is totally unacceptable.

Referee #2 (Comments on Novelty/Model System for Author):

This is an interesting study with important therapeutic implications for neurodegenerative disease including Alzheimer's disease. The authors do a thorough job characterizing VHH-B9 and testing its efficacy in vitro and in vivo, and the manuscript is well written.

Referee #2 (Remarks for Author):

In this study, the authors engineer single domain antibodies from camel to recognize the beta-secretase BACE1 as a proof of concept for the development of CNS therapeutics. One single domain antibody, VHH-B9, binds to an exosite of BACE1 with nanomolar affinity, does not bind the close homolog BACE2, and inhibits processing of APP and Abeta generation in cell-free assays and in primary neuron cultures. Interestingly, VHH-B9 inhibits BACE1 cleavage of APP but not another BACE1 substrate Sez6. The authors then generate an AAV-VHH-B9 vector and show that following hippocampal injection APP-dutch mice exhibit co-localization of VHH-B9 and BACE1 in intracellular compartments and show significantly reduced Abeta 40 and Abeta42 production in the brain. The authors conclude that AAV delivery of engineered single domain antibodies constitute a novel therapeutic approach for targeting a wide range of CNS targets for neurodegenerative diseases.

This is an interesting study with important therapeutic implications for neurodegenerative disease including Alzheimer's disease.

The authors do a thorough job characterizing VHH-B9 and testing its efficacy in vitro and in vivo, and the manuscript is well written. I have only three minor comments:

1. The authors explanation as to why VHH-B9 inhibits BACE1 cleavage of APP but not Sez6 should be elaborated in the discussion. The statement that the substrate selectivity "is most simply explained by the relative amounts of APP and SEZ6 available for cleavage in a mass-action model (Wilhelm et al, 2014)" is rather vague. In addition, it is not immediately clear why compound J would not behave in a similar fashion in such a mass-action model. Other explanations may exist and should be explored, as an APP-selective BACE1 inhibitor would be enormously valuable for avoiding substrate-specific side effects.
2. It is unclear why APP-dutch mice were used instead of a more commonly used APP transgenic model. The authors should explain their rationale for this mouse.
3. Did the authors explore amyloid pathology by immunostainng? If so, please include some Abeta stained sections.

Referee #3 (Comments on Novelty/Model System for Author):

The model systems used so far in this manuscript are adequate. However, I think the authors would need to go a bit further to identify potential functional impact of their promising approach. This could be achieved in a disease mouse model with clear behavioral and neuropathological defects.

Referee #3 (Remarks for Author):

This is an interesting piece of work aimed at generating single domain antibodies (VHH) for therapeutic purposes. The team identified an efficient VHH-B9 following immunization of dromedary and a llama. The VHH generated is targeting the beta-site amyloid precursor protein cleavage enzym 1 (BACE1). The VHH-B9 cDNA was packaged in a AAV vector system for potential gene transfer in vitro and in vivo. They nicely demonstrated that VHH-B9 mediated selective lowering of BACE1 activity in vitro, including neuronal cells. They also report that VHH-B9 led to reduced levels of amyloid beta (Ab) following AAV-mediated delivery of VHH-B9 into CNS of a mouse model of cerebral amyloidosis.

This is an exciting approach with some evidence of efficacy in vitro and in vivo. However, it would be very compelling if the strategy is leading to functional recovery in a mouse model of disease. This could be achieved by measuring impact on behavioral and neuropathological defects. Including such data could take this manuscript to a high and convincing level.

Minor comments:
1) what dose of AAV was delivered in mice?
2) Page 6: line 1: please refer to the correct figure 2 instead to Figure 1.
3) Last paragraph of result section claim no toxicity was observed following delivery in brain of mice. How measurement of toxicity was achieved. Please elaborate further
4) Figure 3F: Y axis description is missing

EMM-2018-09824: Marino et al.: Point-by-point response to the reviewers' comments:
We thank all of the reviewers for highlighting the importance of our findings and the clinical potential. On the advice of the reviewers, we have added substantial experimental data, which can be summarized as follows:

1) Use of an alternative blood brain barrier crossing AAV capsid, PHP.B, allowing intravenous delivery in a next generation Alzheimer's model, *App*$^{NL\text{-}G\text{-}F}$.

2) A long-term, integrated study combining behavioral analysis, to assess possible cognitive improvements in *App*$^{NL\text{-}G\text{-}F}$ mice following AAV-VHH-B9 administration, with subsequent post-mortem analysis of Aβ levels, associated neuroinflammation and neuronal circuit performance.

We greatly appreciated the reviewers' critiques. We believe that by addressing these, we have substantially improved the manuscript. As a result, we have completely redrafted the text using the 'Research Article' format, which we believe is more suited to allow clear presentation and discussion of the additional data generated during the revision period. Below, we provide answers to all the individual concerns raised during the initial review.

Referee #1 (Remarks for Author):
In this manuscript titled "AAV mediated delivery of a novel anti-BACE1 VHH reduces Abeta in an Alzheimer's disease mouse model", Melvin Y. Rincon et al develop a novel VHH (VHH-B9) to inhibit BACE1 activity in vitro and propose a gene transfer strategy to deliver this VHH directly into CNS by means of using AAV as a vector. In vivo injections of AAV encoded VHH-B9 demonstrated reductions in amyloid beta levels in mice model of cerebral amyloidosis. Similar approach using AAV delivered nanobody in different murine model has been tested (Verhelle A., et al., Human Molecular Genetics, 2017). The main weakness of this study is the lack of evidences for therapeutic benefits in the treated animals. More comprehensive analyses will strengthen the story.

We thank the reviewer for his/her insightful comments and believe they have helped us significantly improve the manuscript.

Major points:
1. Lack of data to show the improvements in pathology and cognitive function in treated animals.
We agree with the reviewer that lack of data showing long-term cognitive improvement was a weakness in the original submission. Therefore, we have undertaken a comprehensive set of experiments which show that long-term VHH-B9 expression, following AAV-based delivery, preserves aspects of neuronal circuit function (LTP) (Figure 5) and preserves cognitive function in a 'Sociability and Preference for

Social Novelty' paradigm (Figure 2B). These improvements are associated with significant reductions in $A\beta_{1-40}$ and $A\beta_{1-42}$ levels (Figure 3), reduced astro- and microgliosis (Figure 4) and reduced synaptic loss (Figure EV5B).

2. The authors did not check for long term persistence of VHH-B9 expression in mice brain. Mice were sacked 3 weeks post injection. For therapeutic purposes, considering the route of administration is intracranial, the timelines are much longer, and repeated administration is inconvenient and at times not feasible, monitoring VHH-B9 levels at later time points is important to conclude that there is lack of clearance.

We thank the reviewer for this comment. Amyloidosis develops comparatively slowly in the *App*$^{NL\text{-}G\text{-}F}$ mouse line we used in our experiments. The major increase in insoluble $A\beta_{1-42}$ (the predominant species in these mice) occurs at approximately 4 months of age (Saito *et al*, Nat Neurosci, 2014), with robust behavioral impairments generally only detected after 6 months of age (Saito *et al*, Nat Neurosci, 2014; Mehla *et al*, Neurobiol Aging, 2019; Latif-Hernandez *et al*, Alzheimer Res Ther, 2020). In order to assess the ability of VHH-B9 to efficiently inhibit Aβ accumulation and prevent cognitive decline in these mice, we injected our experimental cohorts at 6 weeks of age and performed behavioral experiments between 10-12 months of age. This was followed by euthanasia for post-mortem electrophysiology, biochemical analysis and histology one year post-injection. Staining for VHH-B9 (cMyc-tag) (Figure 2) confirmed expression at this time, with comparatively high levels in brain regions implicated in sociability and social novelty (Prefrontal Cortex, Dentate Gyrus, Hippocampal CA1, Amygdala) (Bicks *et al*, Front Psychol, 2015; Okuyama *et al,* Science, 2016; Ko, Front Neural Circuits, 2017; Huang *et al*, Cell Rep, 2020; Hainmueller & Bartos, Nat Rev Neurosci, 2020).

3. Did the authors observe any physical manifestations of the reduction in $A\beta_{1-40}$ and $A\beta_{1-42}$ in mice brain like reduction in neuroinflammation or hemorrhages which are typically seen in APP Dutch mice?

We changed our mouse model to the *App*$^{NL\text{-}G\text{-}F}$ line, in response to a comment from Reviewer 2. We chose the *App*$^{NL\text{-}G\text{-}F}$ line as it is a knock-in model (Saito *et al*, Nat Neurosci, 2014) and more likely recapitulates endogenous patterns of APP expression and Aβ production. In this model, we observed that reduction of $A\beta_{1-40}$ and $A\beta_{1-42}$, following VHH-B9 treatment, was associated with reduced neuroinflammation, reduced synaptic loss and improved plasticity of neuronal circuits.

Minor points:

1. Why did the authors select AAV1 as the preferred serotype for in vivo administration over some other serotypes like AAV9 or PHP.B that have been demonstrated in the past to have higher transduction efficiency for brain than AAV1?

The choice of AAV1 as the preferred serotype for direct intraparenchymal injections was largely based on our previous successful use of AAV1 in such protocols. However, in this revised manuscript, we have switched to PHP.B, as this serotype has been shown to give high levels of CNS transduction following intravenous administration, which we believe is a more therapeutically relevant delivery method.

2. The author should examine for any immune responses against either the AAV or VHH-B9. They can conduct simple ELISAs to look for antibodies against the transgene.

We thank the reviewer for this suggestion.

We performed an ELISA to investigate the possible immune response against VHH-B9, and this new data is given in Appendix Figure 2. Under the experimental conditions used, we could not detect a significant immune (humoral) response against VHH-B9, consistent with the known low immunogenicity of VHH. We believe this explains the fact that we see long-term VHH expression in our experiments, in the absence of overt toxicity (Figure 2C), as well as reduced pathology in $App^{NL\text{-}G\text{-}F}$ mice.

We did not perform experiments designed to detect immune response to AAV, as anti-capsid antibodies are known to be rapidly generated following systemic administration (Verdera *et al*, Mol Ther, 2020).

3. The most important data presented in this manuscript is Figure 3E and 3F. However, the authors claimed for a significant reduction of $A\beta_{1\text{-}40}$ and $A\beta_{1\text{-}42}$ in animals without any statistical analysis shown in the data figure. This is totally unacceptable.

We apologize for this oversight. As it stands, we have now replaced the offending figure with new data on $A\beta_{1\text{-}40}$ and $A\beta_{1\text{-}42}$ reduction following intravenous delivery of AAV.PHP.B-VHH-B9, with appropriate statistical analysis (Figure 3). We have also double-checked other figures in the revised manuscript to ensure that statistical analysis is included where appropriate. We hope this satisfies the reviewer.

Referee #2 (Comments on Novelty/Model System for Author):

This is an interesting study with important therapeutic implications for neurodegenerative disease including Alzheimer's disease. The authors do a thorough job characterizing VHH-B9 and testing its efficacy in vitro and in vivo, and the manuscript is well written.

We thank the reviewer for their positive assessment of our work and recognition of its importance.

Referee #2 (Remarks for Author):

In this study, the authors engineer single domain antibodies from camel to recognize the beta-secretase BACE1 as a proof of concept for the development of CNS therapeutics. One single domain antibody, VHH-B9, binds to an exosite of BACE1 with nanomolar affinity, does not bind the close homolog BACE2, and inhibits processing of APP and Abeta generation in cell-free assays and in primary neuron cultures. Interestingly, VHH-B9 inhibits BACE1 cleavage of APP but not another BACE1 substrate Sez6. The authors then generate an AAV-VHH-B9 vector and show that following hippocampal injection APP-dutch mice exhibit co-localization of VHH-B9 and BACE1 in intracellular compartments and show significantly reduced Abeta 40 and Abeta42 production in the brain. The authors conclude that AAV delivery of engineered single domain antibodies constitute a novel therapeutic approach for targeting a wide range of CNS targets for neurodegenerative diseases.

This is an interesting study with important therapeutic implications for neurodegenerative disease including Alzheimer's disease. The authors do a thorough job characterizing VHH-B9 and testing its efficacy in vitro and in vivo, and the manuscript is well written. I have only three minor comments:

1. The authors explanation as to why VHH-B9 inhibits BACE1 cleavage of APP but not Sez6 should be elaborated in the discussion. The statement that the substrate selectivity "is most simply explained by the relative amounts of APP and SEZ6 available for cleavage in a mass-action model (Wilhelm et al, 2014)" is rather vague. In addition, it is not immediately clear why compound J would not behave in a similar fashion in such a mass-action model. Other explanations may exist and should be explored, as an APP-selective BACE1 inhibitor would be enormously valuable for avoiding substrate-specific side effects.

We thank the reviewer for raising this issue.

We agree that an APP-selective BACE1 inhibitor would be of enormous value for avoiding substrate-specific side effects. We respectfully point out, however, that the main focus of the paper is on providing proof-of-concept for AAV-mediated nanobody delivery for long-term treatment of CNS disease. In this respect, we choose BACE1 as a target for nanobody production, as it has proven to be an ideal target for exploring the potential of antibody-based therapeutics in the CNS (Atwal *et al.*, Sci Transl Med, 2011). BACE1 is constitutively active, accounting for the majority of Aβ production *in vivo*. Therefore, measuring Aβ concentrations after anti-BACE1 administration provides a direct measure of antibody-mediated (or nanobody-mediated) target neutralization.

As such, we decided not to emphasize the issue of substrate specificity in the current paper, particularly as we only test one known BACE1 substrate, Sez6. Hence, we have moved these blots to the ‘Appendix’ and restrict mention of the therapeutic potential of such specific inhibitors to the ‘Discussion’.

Our intention is to follow up this (important) observation in a separate project, in which we can explore potential APP specificity by assessing cleavage of a wide range of alternate BACE1 substrates (Barão *et al*, Trends Neurosci, 2016; Hampel *et al*, Biol Psychiatry, 2021).

2. It is unclear why APP-dutch mice were used instead of a more commonly used APP transgenic model. The authors should explain their rationale for this mouse.

Our reason for using the APP-Dutch mouse line was that we had previously used it to show that the anti-BACE1 monoclonal antibody 1A11 inhibits Aβ production *in vivo*, following direct injection into the hippocampus (Zhou *et al*, J Biol Chem, 2011). Hence, we regarded it as an ideal control platform to test the activity of our newly generated anti-BACE1 nanobodies. At the time this particular set of experiments was performed, we also had animals of the right age immediately available for injection.

However, we acknowledge the concerns of the reviewer. As both Reviewers 1 and 3 asked to see a functional impact of VHH-B9 delivery in an Alzheimer's model, we needed to run a set of long-term behavior experiments during the review period. Therefore, we took this opportunity to switch to the commonly used, and well characterized, $App^{NL\text{-}G\text{-}F}$ knock in line (Saito *et al*, Nat Neurosci, 2014).

3. Did the authors explore amyloid pathology by immunostaining? If so, please include some Abeta stained sections.

The requested stainings are now included (Figure 3 and Figure EV5A).

Referee #3 (Comments on Novelty/Model System for Author):

The model systems used so far in this manuscript are adequate. However, I think the authors would need to go a bit further to identify potential functional impact of their promising approach. This could be achieved in a disease mouse model with clear behavioral and neuropathological defects.

We thank the reviewer for their constructive critique of our work. In our revised manuscript, we have acted on these comments and show a clear functional impact of our technology in the $App^{NL\text{-}G\text{-}F}$ mouse line, at both neuropathological and behavioral levels.

Referee #3 (Remarks for Author):

This is an interesting piece of work aimed at generating single domain antibodies (VHH) for therapeutic purposes. The team identified an efficient VHH-B9 following immunization of dromedary and a llama. The VHH generated is targeting the beta-site amyloid precursor protein cleavage enzyme 1 (BACE1). The VHH-B9 cDNA was packaged in a AAV vector system for potential gene transfer in vitro and in vivo. They nicely demonstrated that VHH-B9 mediated selective lowering of BACE1 activity in vitro,

including neuronal cells. They also report that VHH-B9 led to reduced levels of amyloid beta (Ab) following AAV-mediated delivery of VHH-B9 into CNS of a mouse model of cerebral amyloidosis.

This is an exciting approach with some evidence of efficacy in vitro and in vivo. However, it would be very compelling if the strategy is leading to functional recovery in a mouse model of disease. This could be achieved by measuring impact on behavioral and neuropathological defects. Including such data could take this manuscript to a high and convincing level.

As indicated above, during revision, we have added the neuropathological and behavioral data requested.

Minor comments:

1) what dose of AAV was delivered in mice?

We have now switched to intravenous delivery of AAV, rather than using direct parenchymal injection. Using tail vein injection, we routinely administer $1x10^{12}$ vector genomes (vg) in a total solution volume of 100 µl per mouse. We have included this information in the main text and figure legend describing the behavioral experiments performed (Figure 2). Follow up experiments measuring Aβ levels, associated neuroinflammation and neuronal circuit performance (Figures 3-5), were all performed post-mortem on tissue recovered from these mouse cohorts (a detail highlighted in the text).

2) Page 6: line 1: please refer to the correct figure 2 instead to Figure 1.

During the revision period, we have added substantial new data to the manuscript to satisfy the reviewers' comments. This has led to a restructuring of the text. We have taken great care to ensure that all figures are correctly referenced in this resubmitted version.

3) Last paragraph of result section claims no toxicity was observed following delivery in brain of mice. How measurement of toxicity was achieved. Please elaborate further

Here, we were referring to the fact that vector administration and nanobody administration did not lead to abnormal behavior, nor premature death, in the injected mice.

In our revised manuscript, we have performed long-term experiments and shown that this lack of overt toxicity holds 12 months post-injection. We have added this information to the text as Appendix Table 1, containing full details of the mouse cohorts used. To avoid any confusion as to what we mean by 'toxicity' we now refer specifically to 'behavioral abnormalities' and 'mortality rate'. We hope this satisfactorily addresses the issue.

4) Figure 3F: Y axis description is missing

The measurement of $A\beta_{1-42}$ has been repeated following intravenous administration of AAV.PHP.B-VHH-B9 (Figure 3D). We have made sure that the graph axes are correctly labeled.

20th Dec 2021

Thank you for the submission of your revised manuscript to EMBO Molecular Medicine. We have now received the enclosed report from two of the three referees who agreed to re-assess it. Unfortunately, after a series of reminders, we did not obtain a report from Referee #3. In the interest of time, and since the other two referees' recommendations are similar, I prefer to make a decision now rather than further delay the process. As you will see, the referees are now overall supportive, and I am pleased to inform you that we will be able to accept your manuscript pending the following amendments:

https://www.embopress.org/doi/pdf/10.1002/emmm.201000094), EMBO Molecular Medicine will publish online a Review Process File to accompany accepted manuscripts.

In the event of acceptance, this file will be published in conjunction with your paper and will include the anonymous referee reports, your point-by-point response and all pertinent correspondence relating to the manuscript. If you do NOT want this file to be published, please inform the editorial office at contact@embomolmed.org.

To submit your manuscript, please follow this link:

https://embomolmed.msubmit.net/cgi-bin/main.plex

When submitting your revised manuscript, please include:

1) a .docx formatted version of the manuscript text (including Figure legends and tables)

2) Separate figure files*

3) supplemental information as Expanded View and/or Appendix. Please carefully check the authors guidelines for formatting Expanded view and Appendix figures and tables at
https://www.embopress.org/page/journal/17574684/authorguide#expandedview

4) a letter INCLUDING the reviewer's reports and your detailed responses to their comments (as Word file).

5) The paper explained: EMBO Molecular Medicine articles are accompanied by a summary of the articles to emphasize the major findings in the paper and their medical implications for the non-specialist reader. Please provide a draft summary of your article highlighting
- the medical issue you are addressing,
- the results obtained and
- their clinical impact.
This may be edited to ensure that readers understand the significance and context of the research.
Please refer to any of our published articles for an example.

6) For more information: There is space at the end of each article to list relevant web links for further consultation by our readers. Could you identify some relevant ones and provide such information as well? Some examples are patient associations, relevant databases, OMIM/proteins/genes links, author's websites, etc...

7) Author contributions: the contribution of every author must be detailed in a separate section.

8) EMBO Molecular Medicine now requires a complete author checklist (https://www.embopress.org/page/journal/17574684/authorguide) to be submitted with all revised manuscripts. Please use the checklist as guideline for the sort of information we need WITHIN the manuscript. The checklist should only be filled with page numbers were the information can be found. This is particularly important for animal reporting, antibody dilutions (missing) and exact values and n that should be indicted instead of a range.

9) Every published paper now includes a 'Synopsis' to further enhance discoverability. Synopses are displayed on the journal webpage and are freely accessible to all readers. They include a short stand first (maximum of 300 characters, including space) as well as 2-5 one sentence bullet points that summarise the paper. Please write the bullet points to summarise the key NEW findings. They should be designed to be complementary to the abstract - i.e. not repeat the same text. We encourage inclusion of key acronyms and quantitative information (maximum of 30 words / bullet point). Please use the passive voice. Please attach these in a separate file or send them by email, we will incorporate them accordingly.

You are also welcome to suggest a striking image or visual abstract to illustrate your article. If you do please provide a jpeg file 550 px-wide x 400-px high.

10) A Conflict of Interest statement should be provided in the main text

11) Please note that we now mandate that all corresponding authors list an ORCID digital identifier. This takes <90 seconds to complete. We encourage all authors to supply an ORCID identifier, which will be linked to their name for unambiguous name identification.

Currently, our records indicate that the ORCID for your account is 0000-0002-8958-4027.

Please click the link below to modify this ORCID:
Link Not Available

12) The system will prompt you to fill in your funding and payment information. This will allow Wiley to send you a quote for the article processing charge (APC) in case of acceptance. This quote takes into account any reduction or fee waivers that you may be eligible for. Authors do not need to pay any fees before their manuscript is accepted and transferred to our publisher.

*Additional important information regarding Figures

Each figure should be given in a separate file and should have the following resolution:
Graphs 800-1,200 DPI
Photos 400-800 DPI
Colour (only CMYK) 300-400 DPI"

Figures are not edited by the production team. All lettering should be the same size and style; figure panels should be indicated by capital letters (A, B, C etc). Gridlines are not allowed except for log plots. Figures should be numbered in the order of their appearance in the text with Arabic numerals. Each Figure must have a separate legend and a caption is needed for each panel.

*Additional important information regarding figures and illustrations can be found at https://bit.ly/EMBOPressFigurePreparationGuideline. See also figure legend preparation guidelines: https://www.embopress.org/page/journal/17574684/authorguide#figureformat

The system will prompt you to fill in your funding and payment information. This will allow Wiley to send you a quote for the article processing charge (APC) in case of acceptance. This quote takes into account any reduction or fee waivers that you may be eligible for. Authors do not need to pay any fees before their manuscript is accepted and transferred to our publisher.

***** Reviewer's comments *****

Referee #1 (Remarks for Author):

The authors have addressed this reviewer's concerns adequately. The revised manuscript is significantly improved.

Referee #2 (Comments on Novelty/Model System for Author):

The authors have adequately addressed the comments of the previous review.

Referee #2 (Remarks for Author):

The authors have adequately addressed the comments of the previous review.

EMM-2018-09824: Marino et al.: Response to reviewer's comments

Referee #1 (Remarks for Author):

The authors have addressed this reviewer's concerns adequately. The revised manuscript is significantly improved.

Referee #2 (Comments on Novelty/Model System for Author):

The authors have adequately addressed the comments of the previous review.

Referee #2 (Remarks for Author):

The authors have adequately addressed the comments of the previous review.

We thank the reviewers for their constructive critiques. The additional experimental data they requested significantly improved the quality of the manuscript, highlighting the importance of our findings and their clinical potential. We were pleased to learn that they now consider the current version of the manuscript suitable for publication in EMBO Molecular Medicine.

14th Feb 2022

We are pleased to inform you that your manuscript is accepted for publication and is now being sent to our publisher to be included in the next available issue of EMBO Molecular Medicine.

We would like to remind you that as part of the EMBO Publications transparent editorial process initiative, EMBO Molecular Medicine will publish a Review Process File online to accompany accepted manuscripts. If you do NOT want the file to be published or would like to exclude figures, please immediately inform the editorial office via e-mail.

Please read below for additional IMPORTANT information regarding your article, its publication and the production process.

Congratulations on your interesting work,

Jingyi

Jingyi Hou
Editor
EMBO Molecular Medicine

Follow us on Twitter @EmboMolMed
Sign up for eTOCs at embopress.org/alertsfeeds

*** *** *** IMPORTANT INFORMATION *** *** ***

SPEED OF PUBLICATION
The journal aims for rapid publication of papers, using the advance online publication "Early View" to expedite the process: A properly copy-edited and formatted version will be published as "Early View" after the proofs have been corrected. Please help the Editors and publisher avoid delays by providing e-mail address(es), telephone and fax numbers at which author(s) can be contacted.

Should you be planning a Press Release on your article, please get in contact with embomolmed@wiley.com as early as possible, in order to coordinate publication and release dates.

LICENSE AND PAYMENT:

All articles published in EMBO Molecular Medicine are fully open access: immediately and freely available to read, download and share.

EMBO Molecular Medicine charges an article processing charge (APC) to cover the publication costs. You, as the corresponding author for this manuscript, should have already received a quote with the article processing fee separately. Please let us know in case this quote has not been received.

Once your article is at Wiley for editorial production you will receive an email from Wiley's Author Services system, which will ask you to log in and will present you with the publication license form for completion. Within the same system the publication fee can be paid by credit card, an invoice, pro forma invoice or purchase order can be requested.

Payment of the publication charge and the signed Open Access Agreement form must be received before the article can be published online.

PROOFS

You will receive the proofs by e-mail approximately 2 weeks after all relevant files have been sent o our Production Office. Please return them within 48 hours and if there should be any problems, please contact the production office at embopressproduction@wiley.com.

Please inform us if there is likely to be any difficulty in reaching you at the above address at that time. Failure to meet our deadlines may result in a delay of publication.

All further communications concerning your paper proofs should quote reference number EMM-2018-09824-V3 and be directed to the production office at embopressproduction@wiley.com.

Thank you,

Jingyi Hou
Editor
EMBO Molecular Medicine

EMBO PRESS

YOU MUST COMPLETE ALL CELLS WITH A PINK BACKGROUND ↓

PLEASE NOTE THAT THIS CHECKLIST WILL BE PUBLISHED ALONGSIDE YOUR PAPER

Corresponding Author Name: Matthew G. Holt

Journal Submitted to: EMBO Molecular Medicine
Manuscript Number: EMM-2018-09824

USEFUL LINKS FOR COMPLETING THIS FORM

http://www.antibodypedia.com
http://1degreebio.org
http://www.equator-network.org/reporting-guidelines/improving-bioscience-research-repor
http://grants.nih.gov/grants/olaw/olaw.htm
http://www.mrc.ac.uk/Ourresearch/Ethicsresearchguidance/Useofanimals/index.htm
http://ClinicalTrials.gov
http://www.consort-statement.org
http://www.consort-statement.org/checklists/view/32-consort/66-title
http://www.equator-network.org/reporting-guidelines/reporting-recommendations-for-tumo
http://datadryad.org
http://figshare.com
http://www.ncbi.nlm.nih.gov/gap
http://www.ebi.ac.uk/ega
http://biomodels.net/
http://biomodels.net/miriam/
http://jjj.biochem.sun.ac.za
https://osp.od.nih.gov/biosafety-biosecurity-and-emerging-biotechnology/
http://www.selectagents.gov/

Reporting Checklist For Life Sciences Articles (Rev. June 2017)

This checklist is used to ensure good reporting standards and to improve the reproducibility of published results. These guidelines are consistent with the Principles and Guidelines for Reporting Preclinical Research issued by the NIH in 2014. Please follow the journal's authorship guidelines in preparing your manuscript.

A- Figures
1. Data
The data shown in figures should satisfy the following conditions:

- the data were obtained and processed according to the field's best practice and are presented to reflect the results of the experiments in an accurate and unbiased manner.
- figure panels include only data points, measurements or observations that can be compared to each other in a scientifically meaningful way.
- graphs include clearly labeled error bars for independent experiments and sample sizes. Unless justified, error bars should not be shown for technical replicates.
- if n< 5, the individual data points from each experiment should be plotted and any statistical test employed should be justified
- Source Data should be included to report the data underlying graphs. Please follow the guidelines set out in the author ship guidelines on Data Presentation.

2. Captions
Each figure caption should contain the following information, for each panel where they are relevant:

- a specification of the experimental system investigated (eg cell line, species name).
- the assay(s) and method(s) used to carry out the reported observations and measurements
- an explicit mention of the biological and chemical entity(ies) that are being measured.
- an explicit mention of the biological and chemical entity(ies) that are altered/varied/perturbed in a controlled manner.

- the exact sample size (n) for each experimental group/condition, given as a number, not a range;
- a description of the sample collection allowing the reader to understand whether the samples represent technical or biological replicates (including how many animals, litters, cultures, etc.).
- a statement of how many times the experiment shown was independently replicated in the laboratory.
- definitions of statistical methods and measures:
  - common tests, such as t-test (please specify whether paired vs. unpaired), simple χ2 tests, Wilcoxon and Mann-Whitney tests, can be unambiguously identified by name only, but more complex techniques should be described in the methods section;
  - are tests one-sided or two-sided?
  - are there adjustments for multiple comparisons?
  - exact statistical test results, e.g., P values = x but not P values < x;
  - definition of 'center values' as median or average;
  - definition of error bars as s.d. or s.e.m.

Any descriptions too long for the figure legend should be included in the methods section and/or with the source data.

In the pink boxes below, please ensure that the answers to the following questions are reported in the manuscript itself. Every question should be answered. If the question is not relevant to your research, please write NA (non applicable). We encourage you to include a specific subsection in the methods section for statistics, reagents, animal models and human subjects.

B- Statistics and general methods

Please fill out these boxes ↓ (Do not worry if you cannot see all your text once you press return)

1.a. How was the sample size chosen to ensure adequate power to detect a pre-specified effect size?	Two sets of experiments were performed, an initial proof-of-concept (PoC) to test VHH production and target engagement following systemic (tail vein) AAV injection, and a longitudinal study (BEHAVIOR) to assess the possible beneficial effects of extended VHH expression/activity. Both sets of experiments were performed in the AppNL-G-F mouse line. PoC: Experiments using cultured neurons (Figure 1D) showed a significant reduction in amyloid beta production following VHH-B9 administration. Based on this data, we designed PoC experiments assuming an effect size of 2 and assuming 80% power and an alpha of 0.05, giving a minimum of six animals in each treatment cohort. BEHAVIOR: Based on previous experiments with the AppNL-G-F line, an effect size of 1.36 was expected (Latif-Hernandez et al. (2019) Subtle behavioral changes and increased prefrontal-hippocampal synchronicity in AppNL-G-F mice before dominant plaque deposition. Behav Brain Res, 17: 431-441). Assuming 90% power and an alpha of 0.05, a minimal sample size of n=13 per group was calculated. However, to offset possible loss of animals due to aging-related issues (including potential long-term effects of vector injections and/or onset of AD-type pathology), we added n=3-5 to the starting groups, to account for potential losses. Similar sample size was adopted for the C57Bl/6J controls (Figure Legends; Appendix Table S1)
1.b. For animal studies, include a statement about sample size estimate even if no statistical methods were used.	PoC: Sample size estimates were made based on reductions in amyloid beta production seen after testing VHH-B9 efficacy in tissue culture experiments (Figure 1D). BEHAVIOR: The sample size for our long-term animal study was estimated based on previous observations with the AppNL-G-F line, taking into account the necessity to perform behavioural studies in aged animals, with overt AD pathology and viral vector treatment both potentially contributing to premature death (relative to BL6 controls).
2. Describe inclusion/exclusion criteria if samples or animals were excluded from the analysis. Were the criteria pre-established?	PoC: No animals were excluded from the analysis. BEHAVIOR: Animals were excluded in the case of technical problems (i.e. incorrect tracking). In addition, non-performers were removed from analysis of data obtained using the Morris Water Maze, based on pre-established criteria (swim velocity < 5cm/s for longer than 35% of total time).
3. Were any steps taken to minimize the effects of subjective bias when allocating animals/samples to treatment (e.g. randomization procedure)? If yes, please describe.	PoC and BEHAVIOR: Particular care was put into avoiding any potential subjective bias during allocation of the animals into treatment groups; Assignment to AAV treatment groups was performed randomly. PoC: Both male and female animals were used (Appendix Table S1). No blinding was performed in respect of the vector treatments given to each experimental cohort. BEHAVIOR: To avoid any potential gender-dependent effects, all the animals included in the long-term study were males. During vector administration, the scientist performing injections was blinded to the AAV-type being administered. The C57Bl/6J non-injected group was simply established using the n=18 animals that were left over following AAV vector administration, generating a comparable control group.
For animal studies, include a statement about randomization even if no randomization was used.	PoC and BEHAVIOR: Allocation of the animals into each treatment group was performed randomly. BEHAVIOR: the order the animals were used in testing was randomised and the tester was blinded to treatment group.

4.a. Were any steps taken to minimize the effects of subjective bias during group allocation or/and when assessing results (e.g. blinding of the investigator)? If yes please describe.	PoC: The identity of treatment groups was known to the investigator performing the amyloid beta ELISAs and the IHCs for VHH expression. BEHAVIOR: To avoid observation bias, behavioural studies were performed blinded and group association was coded. Identification of the animals during the test was ensured by a digital signature in the tracking software, based on the color of different non-toxic paints used to color the tail. The colors were randomly assigned to the animals inside each cage. Collection of data was based on automated tracking.
4.b. For animal studies, include a statement about blinding even if no blinding was done	POC: No blinding was performed. BEHAVIOR: Tests were performed blinded and experimental group was coded.
5. For every figure, are statistical tests justified as appropriate?	Appropriate statistical tests were chosen based on the scope of the analysis, type of data to be analysed (paired vs. unpaired) and its distribution. The specific test used is reported in the figure legend of each graph.
Do the data meet the assumptions of the tests (e.g., normal distribution)? Describe any methods used to assess it.	The data met the assumptions of each statistical test used, specifically normal distribution (Shapiro-Wilk test) and/or variance (F test). These criteria were verified using Graphpad Prism 9.2.0 software.
Is there an estimate of variation within each group of data?	Yes, variation within each data set is expected due to biological factors (e.g. different rates of amyloid beta accumulation). The coefficient of variation (Cv) was calculated with the aid of Graphpad Prism 9.2.0 software.
Is the variance similar between the groups that are being statistically compared?	Similar variance between groups was taken into account in our analysis, and the ANOVA test model assumed sphericity. Additionally, to avoid errors dependent on unequal variance, t tests were always performed using Welch's Correction, which does not assume equal standard deviations (SD) between two populations [Ruxton GD (2006) The unequal variance t-test is an underused alternative to Student's t-test and the Mann-Whitney U test. Behavioral Ecology, 17: 688-690; Delacre M et al. (2017) Why Psychologists Should by Default Use Welch's t-test Instead of Student's t-test. International Review of Social Psychology 30: 92–101].

C- Reagents

6. To show that antibodies were profiled for use in the system under study (assay and species), provide a citation, catalog number and/or clone number, supplementary information or reference to an antibody validation profile. e.g., Antibodypedia (see link list at top right), 1DegreeBio (see link list at top right).	The catalog number and/or clone number of each antibody used in the experiments was provided in the manuscript text (Materials and Methods section)
7. Identify the source of cell lines and report if they were recently authenticated (e.g., by STR profiling) and tested for mycoplasma contamination.	NA

* for all hyperlinks, please see the table at the top right of the document

D- Animal Models

8. Report species, strain, gender, age of animals and genetic modification status where applicable. Please detail housing and husbandry conditions and the source of animals.	Related to Materials and Methods section. In this study, C57Bl/6J and AppNL-G-F (strain name: Apptm3.1Tcs/Apptm3.1Tcs) mice were used. The animals involved in the PoC were of mixed sex, while the animals involved in the long-term experiment were males (Appendix Table S1). All animals, with the exception of a non-injected control cohort (C57Bl/6J) in the long-term study, were injected with AAV at 6 weeks of age. Animals were aged for short-term (PoC) and long-term (BEHAVIOR) evaluation, being euthanized 3 months or 12 months post-vector administration, respectively. Prior to AAV vector administration, the animals were kept in a specific pathogen free (SPF) facility. Following AAV injection, the animals were moved to a conventional facility with controlled humidity, temperature and light conditions. All the animals were housed in groups (maximum 5 animals/cage) within individually ventilated (IVC) cages. Following transfer to the behavioural core facility, the animals involved in the long-term study were housed in groups (max 4 animals/cage) within filter top cages. All the cages were provided with wood chip bedding and enriched with materials allowing nest building or concealment. Food and water were provided ad libitum.
9. For experiments involving live vertebrates, include a statement of compliance with ethical regulations and identify the committee(s) approving the experiments.	Releated to Materials and Methods section. All animal procedures were performed in accordance with the regulations of the Institutional Animal Care and Use Committee of KU Leuven (ethical permission document P209/2015).
10. We recommend consulting the ARRIVE guidelines (see link list at top right) (PLoS Biol. 8(6), e1000412, 2010) to ensure that other relevant aspects of animal studies are adequately reported. See author guidelines, under 'Reporting Guidelines'. See also: NIH (see link list at top right) and MRC (see link list at top right) recommendations. Please confirm compliance.	We confirm that animals experiments were planned responsibly. Animals were handled with care throughout experimental procedures and the reporting was conducted in compliance with the ARRIVE guidelines.

E- Human Subjects

11. Identify the committee(s) approving the study protocol.	NA
12. Include a statement confirming that informed consent was obtained from all subjects and that the experiments conformed to the principles set out in the WMA Declaration of Helsinki and the Department of Health and Human Services Belmont Report.	NA
13. For publication of patient photos, include a statement confirming that consent to publish was obtained.	NA
14. Report any restrictions on the availability (and/or on the use) of human data or samples.	NA
15. Report the clinical trial registration number (at ClinicalTrials.gov or equivalent), where applicable.	NA
16. For phase II and III randomized controlled trials, please refer to the CONSORT flow diagram (see link list at top right) and submit the CONSORT checklist (see link list at top right) with your submission. See author guidelines, under 'Reporting Guidelines'. Please confirm you have submitted this list.	NA
17. For tumor marker prognostic studies, we recommend that you follow the REMARK reporting guidelines (see link list at top right). See author guidelines, under 'Reporting Guidelines'. Please confirm you have followed these guidelines.	NA

F- Data Accessibility

18: Provide a "Data Availability" section at the end of the Materials & Methods, listing the accession codes for data generated in this study and deposited in a public database (e.g. RNA-Seq data: Gene Expression Omnibus GSE39462, Proteomics data: PRIDE PXD000208 etc.) Please refer to our author guidelines for 'Data Deposition'.<br><br>Data deposition in a public repository is mandatory for:<br>a. Protein, DNA and RNA sequences<br>b. Macromolecular structures<br>c. Crystallographic data for small molecules<br>d. Functional genomics data<br>e. Proteomics and molecular interactions	NA
19. Deposition is strongly recommended for any datasets that are central and integral to the study; please consider the journal's data policy. If no structured public repository exists for a given data type, we encourage the provision of datasets in the manuscript as a Supplementary Document (see author guidelines under 'Expanded View' or in unstructured repositories such as Dryad (see link list at top right) or Figshare (see link list at top right).	NA
20. Access to human clinical and genomic datasets should be provided with as few restrictions as possible while respecting ethical obligations to the patients and relevant medical and legal issues. If practically possible and compatible with the individual consent agreement used in the study, such data should be deposited in one of the major public access-controlled repositories such as dbGAP (see link list at top right) or EGA (see link list at top right).	NA
21. Computational models that are central and integral to a study should be shared without restrictions and provided in a machine-readable form. The relevant accession numbers or links should be provided. When possible, standardized format (SBML, CellML) should be used instead of scripts (e.g. MATLAB). Authors are strongly encouraged to follow the MIRIAM guidelines (see link list at top right) and deposit their model in a public database such as Biomodels (see link list at top right) or JWS Online (see link list at top right). If computer source code is provided with the paper, it should be deposited in a public repository or included in supplementary information.	NA

G- Dual use research of concern

22. Could your study fall under dual use research restrictions? Please check biosecurity documents (see link list at top right) and list of select agents and toxins (APHIS/CDC) (see link list at top right). According to our biosecurity guidelines, provide a statement only if it could.	NA
